# Covalency control of photomagnetic relaxation in a manganese(II) photoswitch

**DOI:** 10.1038/s41557-026-02193-8

**Published:** 2026-06-29

**Authors:** Sandra Kronenberger, Robert Naumann, Christoph Förster, Jan Klett, Dieter Schollmeyer, Luca M. Carrella, Eva Rentschler, Katja Heinze

**Affiliations:** Department of Chemistry, Johannes Gutenberg University, Mainz, Germany

**Keywords:** Photochemistry, Magnetic materials

## Abstract

Switching the electronic spin state in molecules by light is achieved via chemical bond reshuffling or light-induced excited spin state trapping. These procedures either require large-amplitude nuclear motions or cryogenic temperatures limiting their application in data storage or quantum computing devices. Here we show that a carbene manganese(II) complex undergoes light-induced excited spin state trapping at 141 K in solution and 112.5 K in the solid state. Irradiation quantitatively switches from low- to high-spin in solution and in the solid. The lifetime of the metastable high-spin state extrapolated from experimental data amounts to over 1 million years at 77 K. Key to success for the very long lifetime is the high reorganization energy due to high metal–ligand bond covalency, and hence, a large barrier height and width for the spin relaxation, paving the way for photo-switchable magnetic materials operating at ambient temperature.

**Figure:**
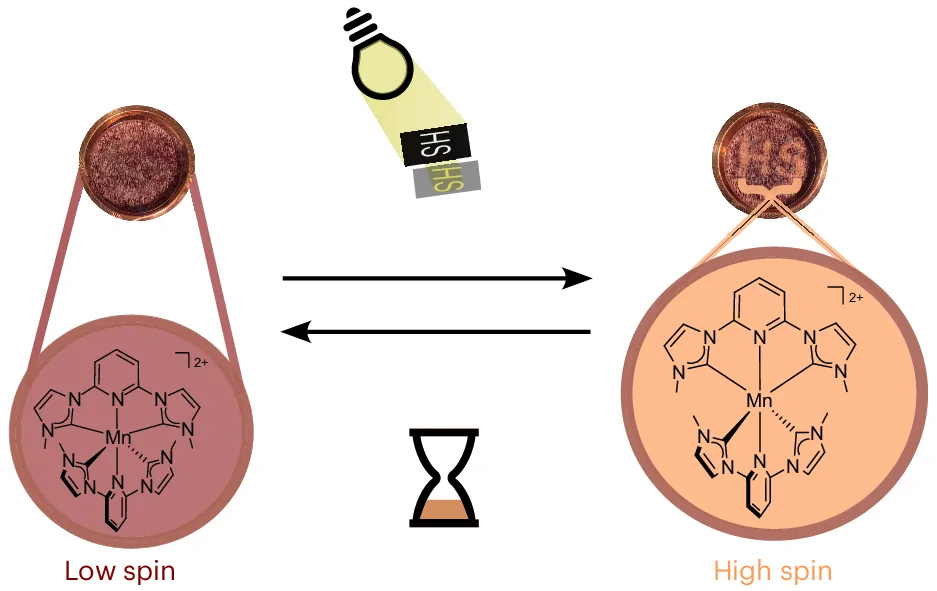

## Abstract (web summary)

Light-induced excited spin state trapping (LIESST) has largely been limited to iron complexes and typically requires cryogenic temperatures or large nuclear rearrangements, limiting practical photomagnetic switching. Now a carbene manganese(II) complex achieves LIESST at 141 K in solution, demonstrating that increased metal–ligand covalency and rigidity can stabilize long-lived high-spin states and extend photomagnetic switching towards more practical temperature regimes.

## Main

Modern information technology depends on controlling physical properties (electrical, magnetic and optical) to store and process data. To meet demands for device miniaturization, researchers are exploring single molecules as information units. Photoswitches, which can reversibly change properties when exposed to light, are a key focus. A promising direction is controlling the spin state of molecules with magnetic bistability by light. Spin states of organic molecules have been switched by light from singlet closed shell (*S* = 0) to diradicaloid states (*S* = 1) with a change of spin of Δ*S* = 1 using configurational isomerization (*cis*-*trans* isomerization) or a chemical reaction (bond homolysis or pericyclic reactions)^1,2^. Both processes typically require major nuclear reorganization. However, the diverse spin states of transition metal complexes open an even richer portfolio of potential photoinduced spin-switches, including materials with larger spin state changes of Δ*S* = 2. A combination of photoinduced *trans*–*cis* isomerization in the ligand with a change of coordination number^3^ (coordination-induced spin state change)^4^ or ligand substitution at nickel(II), iron(III) or molybdenum(III) centres led to bistable systems with Δ*S* = 1 (refs. ^3,5,6^). Again, these spin state switching processes are associated with large-amplitude nuclear motions.

**Fig. 1:**
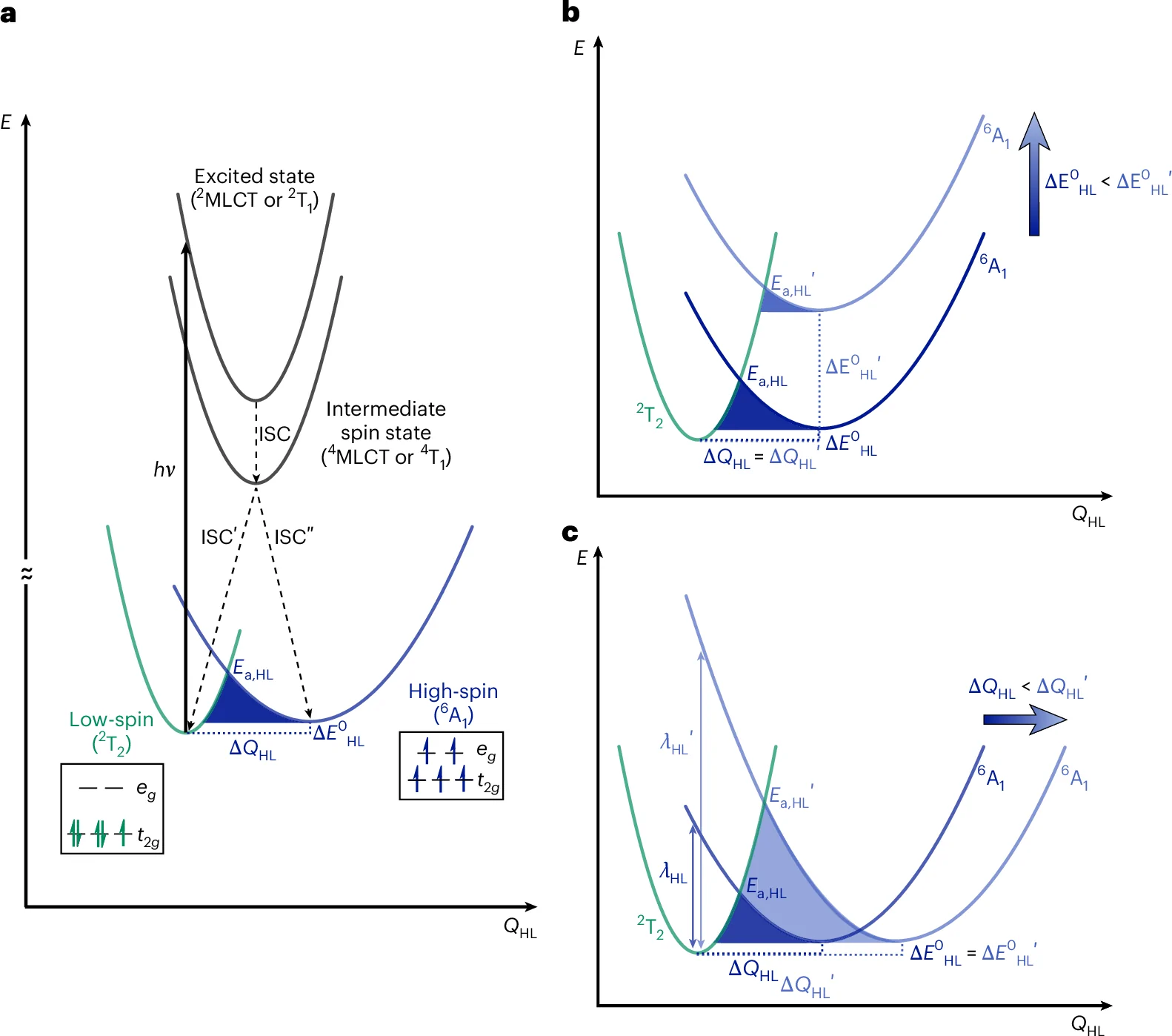
Mechanism of the LIESST effect and concepts to increase the lifetime of the metastable HS state by enlarging the HS → LS barrier. **a**, A general scheme for LIESST using potential wells for the LS (green) and HS (blue) states displaced along the totally symmetric normal coordinate *Q*_HL_(a_1_) by Δ*Q*_HL_ as well as of excited quartet states at higher energy. The term symbols in parentheses refer to a *d*^5^ electron configuration in octahedral symmetry with the respective *t*_2*g*_ and *e*_*g*_ orbital occupation given in the boxes. ISC, ISC′ and ISC′′ denote (horizontal) intersystem crossing followed by (vertical) vibrational relaxation. Δ*E*^0^_HL_ and *E*_a,HL_ refer to the zero-point energy difference between the HS and LS states and the HS → LS energy barrier, respectively. **b**, An illustration of the inverse energy gap law for the HS → LS relaxation^23,26,27^: displacing the HS potential well (blue) vertically to larger Δ*E*^0^_HL_ values (light blue) reduces barrier height (*E*_a,HL_) and width. **c**, An illustration of the present covalency concept: more covalent, that is, stronger M–L bonds, shift the HS potential well (blue) horizontally to larger displacements Δ*Q*_HL_ (light blue), which leads to larger reorganization energies *λ*_HL _→ *λ*_HL_′ and higher HS → LS barriers *E*_a,HL_ → *E*_a,HL_′.

By contrast, octahedral metal complexes with electron configurations *d*^4^–*d*^7^ and a suitable ligand field strength can undergo thermal spin crossover (SCO)^7,8^ from the low-spin (LS; maximum number of paired *d* electrons) to the high-spin (HS; maximum number of unpaired *d* electrons) state without bond breaking/bond formation or large-amplitude nuclear motions. The pre-requisite is a carefully designed ligand field strength so that the zero-point energy difference between the HS and LS states Δ*E*^0^_HL_ = *E*_HS_ – *E*_LS_ (Fig. 1a) is positive and of the order of the thermal energy *k*_B_*T*. The thermal LS → HS transition is an entropy-driven process with ‘softer’ degrees of freedom in the HS state owing to its increased intramolecular vibrations being thermally accessible. Transition temperatures *T*_1/2_, where LS and HS fractions are equal, have been reported between *T*_1/2_ = 100 and *T*_1/2_ = 350 K. The largest body of work on SCO focused on iron-based systems, while only a few examples exist for other metal ions, such as chromium(II)^9,10^, manganese(II)^11,12^, manganese(III)^13,14^ and cobalt(II)^15–17^.

SCO with LS to HS transitions comprising Δ*S* = 2 can also be induced by light. This phenomenon was first observed in 1982, when laser excitation of LS iron(II) complexes transiently populates the HS state at room temperature^18^. Only 2 years later, the photogenerated HS state was trapped at cryogenic temperatures, establishing the phenomenon as light-induced excited spin state trapping (LIESST)^19^. So far, this phenomenon has been restricted to *d*^6^-iron(II) and *d*^5^-iron(III) complexes^20–22^. With the LS → HS transition, the Fe–L bond lengths expand, with Δ*d*_FeL_ ≈ 0.21 and 0.13 Å for iron(II) and iron(III) complexes, respectively^20,21^. This nuclear rearrangement is much less pronounced than for isomerization reactions or bond dissociations, but is sufficiently large to induce an appreciable activation barrier *E*_a,HL_ to retard the relaxation of the metastable HS state to the LS ground state (Fig. 1a).

Figure 1a illustrates a minimalistic description of the LIESST effect for an octahedral *d*^5^ metal complex system, for example iron(III) or manganese(II): excitation of the LS ground state (^2^T_2_, *S* = 1/2) with light populates an excited doublet charge transfer or ligand field state (^2^CT or ^2^T_1_, *S* = ½) with the same multiplicity as the ground state. Intersystem crossing (ISC) furnishes an intermediate spin state (^4^CT or ^4^T_2_, *S* = 3/2), which can either relax back to the ground state (ISC′) or undergo another ISC′′ to the final HS state (^6^A_1_, *S* = 5/2). In addition to the double ISC yielding Δ*S* = 2, internal conversions (ICs) can transiently populate further intermediate excited states on the way to the HS state. Such potential additional intermediate excited states are omitted in Fig. 1b for clarity reasons. In most cases, observation of the LIESST effect requires cryogenic temperatures to trap the HS state^20–22^. Polypyridine iron(II) complexes possess HS → LS barriers of a few hundred wavenumbers (*E*_a,HL_ ≤ 0.09 eV) as determined by variable-temperature transient absorption (TA) spectroscopy in solution^23^. This small barrier does not trap the HS state despite the weak electronic coupling between the HS and LS potentials of a few wavenumbers only (*H*_AB_ = 4–6 cm^−1^, estimated according to a semi-classical Marcus theory approach)^23^.

The characteristic temperature *T*_LIESST_ denotes the limit temperature above which a photomagnetic effect in a material is erased by warming the material from very low temperatures at a rate of 0.3 K min^−1^ (ref. ^20^). LIESST has been observed only for iron(II) and iron(III) complexes, which show thermal SCO in the solid state. The highest *T*_LIESST_ value has been reported for a macrocyclic dicyanido *d*^6^ iron(II) complex with *T*_LIESST_ = 132 K in the solid state^24,25^. As *T*_LIESST_ cannot exceed *T*_1/2_, high *T*_LIESST_ values require high *T*_1/2_ values, preferably above room temperature. High *T*_1/2_ values in turn can only be achieved by stabilizing the LS state (destabilizing the HS state), that is, by increasing the zero-point energy difference Δ*E*^0^_HL_.

However, larger energy differences Δ*E*^0^_HL_′ concomitantly increase the HS → LS relaxation rates owing to the resulting smaller and narrower barrier *E*_a,HL_′, according to the so-called inverse energy gap law^26,27^ as illustrated in Fig. 1b, mitigating the observation of LIESST. This dilemma can be circumvented by simultaneously increasing the distortion (Δ*Q*_HL_′), that is, the bond length changes associated with the spin transition (but without large-amplitude nuclear motions such as bond breaking), in addition to the large zero-point energy difference Δ*E*^0^_HL_ (Fig. 1c). However, a limit for bond length changes of Δ*d*_ML_ ≈ 0.21 Å seems to be reached with iron(II) SCO systems^20,21^.

## Results and discussion

### The covalency concept

**Fig. 2:**
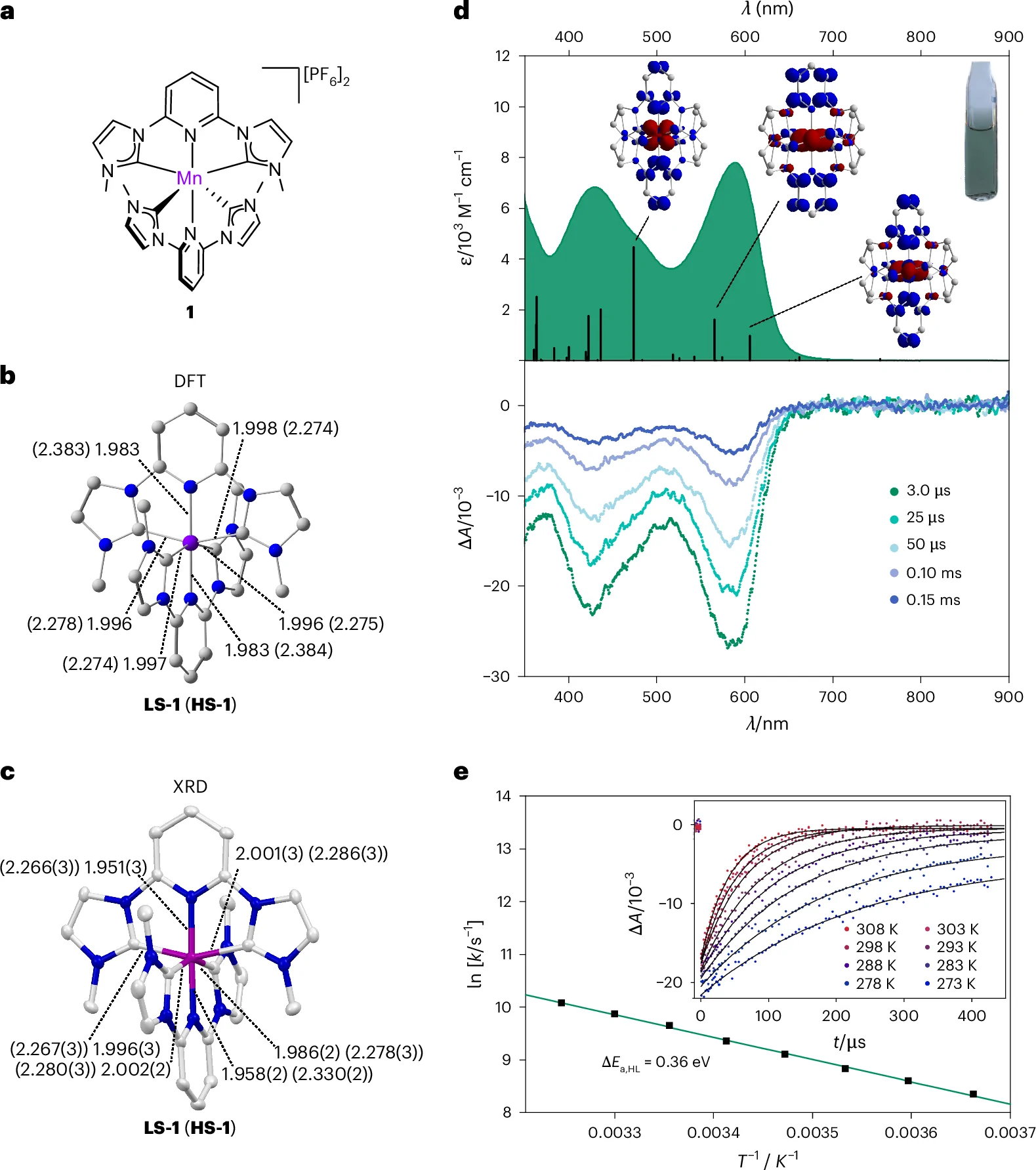
Structural and spectroscopic data of **1**. **a**, The molecular structure of **1**. **b**, The DFT-optimized geometry of **LS-1**, with selected computed bond lengths of **LS-1** and **HS-1** in parentheses (in angstroms). **c**, The molecular structure of one of the two independent cations of **LS-1** in the asymmetric unit shown with thermal ellipsoids at the 50% probability level determined by SCXRD at 91 K and selected weighted average bond lengths of the two independent cations in the unit cell (in angstroms). Counter ions and hydrogen atoms are omitted. The weighted average bond lengths of the light-induced **HS-1** structure obtained from SCXRD at 91 K under irradiation with 560 nm are shown in parentheses. **d**. UV–visible absorption spectrum of **1** in MeCN at 293 K (filled green spectrum) with TDDFT-calculated oscillator strengths (black, shifted by 0.40 eV to lower energies^42,43^. B3LYP/Def2-TZVP/CPCM(acetonitrile)/D3BJ) and electron density difference maps of the bright ^2^MLCT(8), ^2^MLCT(11) and ^2^MLCT(16) Franck–Condon states showing electron density gain (blue) and depletion (red). An isosurface value of 0.003 a.u., hydrogen atoms omitted. A photograph of a solution of **1** in MeCN at 293 K and TA spectra of **1** in MeCN after excitation with 590 nm at 293 K. **e**, Kinetic traces obtained by TA spectroscopy at various temperatures (inset: *λ*_obs_(averaged) = 380–510 nm) and Arrhenius plot of the HS → LS recovery of **1** obtained from the variable-temperature TA spectra using excitation with *λ*_exc_ = 590 nm in MeCN between 273 and 308 K.

Our design strategy for a strongly distorted HS state at high energy, that is, large Δ*E*^0^_HL_ and large Δ*Q*_HL_ values (Fig. 1b,c), is based on strongly *σ*-donating carbene ligands combined with a manganese(II) central ion forming highly covalent Mn–C bonds. This contrasts the commonly employed more ionic Fe^II^–L and Fe^III^–L bonds. The higher Mn–L covalency increases the *σ*-bonding strength, resulting in substantial destabilization of the *e*_g_* orbitals relative to the *t*_2*g*_ orbitals and consequently leads to a higher-energy HS state. With the *d*^6^ manganese(I) complex [Mn(pbmi)_2_]^+^, we already succeeded in destabilizing higher-energy metal-centred states by increased Mn–L covalency to such an extent that a very long-lived luminescent excited state was achieved ((pyridine-2,6-diyl)bis(3-methylimidazol-2-ylidene), pbmi)^28^. On the basis of these results, we now hypothesized that *d*^5^ manganese(II) ions could also form sufficiently strong covalent bonds with carbenes that destabilize the HS state (large Δ*E*^0^_HL_′) and concomitantly impose large Mn–C bond lengths in the HS state (large Δ*Q*_HL_′) by populating high-energy Mn–L *σ*-antibonding orbitals in the HS state. In an extreme case, Mn–L bonds might even break. Yet, Mn–L bond dissociation will be prohibited by installing rigid tridentate ligands with five-membered chelate rings. These rigid ligands should also lead to steeper potential wells, that is, larger force constants in the LS and HS states. The proposed sufficiently large nuclear reorganization energy *λ*_HL_′ (Fig. 1c) without ligand dissociation should increase and broaden the HS → LS relaxation barrier, that is, slow down thermally activated relaxation and tunnelling processes. The pseudo-octahedral carbene pyridine manganese(II) complex [Mn(pbmi)_2_][PF_6_]_2_, **1** (Fig. 2a), has been designed to possess strong *σ*-donation by the carbenes, covalent Mn–L bonding, a rarely observed LS ground state for manganese(II)^29–34^ and high rigidity combined with the *d*^5^ electron configuration giving the favourable Δ*S* = 2 spin-state change for the HS–LS transitions.

Computational modelling of **1** in its LS and HS states (**LS-1** and **HS-1**) using density functional theory (DFT; B3LYP functional) suggests that **LS-1** represents the ground state as expected (Fig. 2b and Supplementary Tables 1–4). Although energy differences of states with different multiplicity are highly functional-dependent^35–38^, the employed B3LYP functional typically favours the HS state^39^. Hence, the LS state is confidently assigned as the ground state. In **LS-1**, the *d*_*x*2–*y*2_-derived *e*_g_* orbital lying in the MnC_4_ plane is even higher in energy than the *d*_*z*2_ orbital pointing along the N–Mn–N axis by 0.83 eV, so that population of this orbital in the HS state would imply a large energy penalty. Moreover, geometries of LS and HS states are generally well reproduced by DFT calculations. Importantly, the Mn–C and Mn–N bond lengths in the **LS-1** and **HS-1** states increase from 1.996 and 1.983 Å to 2.275 and 2.384 Å, respectively (Fig. 2b and Supplementary Table 1). The calculated differences thus amount to Δ*d*_MnC,DFT_ = 0.279 Å and Δ*d*_MnN,DFT_ = 0.401 Å, respectively. In particular, the elongation of the Mn–N bond’s Δ*d*_MnN_ substantially surpasses the analogous expansion of iron(II) and iron(III) SCO complexes^20,21^. Hence, our molecular design based on metal–ligand covalency, as seen in the energies of the *d*_*x*2–*y*2_ and *d*_*z*2_ molecular orbitals (Supplementary Tables 3 and 4), should horizontally shift the HS potential surface to higher displacements of *Q*_HL_, thus increasing Δ*Q*_HL_ (Fig. 1c).

### Synthesis and characterization

The manganese(II) complex **1** is obtained by oxidation of the known parent manganese(I) complex cation^28^ with ferrocenium hexafluorophosphate^40^ as a green solid at 293 K (Supplementary Fig. 1). Magnetic susceptibility (*χ*_m_) measurements of the powder (2–300 K, *χ*_m_*T* = 0.50 cm^3^ K mol^−1^) (Supplementary Fig. 2) confirms the LS state of **1** over this temperature range. The high-temperature *χ*_m_*T* value exceeds the spin-only value of *χ*_m,so_*T* = 0.37 cm^3^ K mol^−1^, as expected for a LS *d*^5^ electron configuration and found previously for the LS *d*^5^ complexes K_4_[Mn(CN)_6_]·3H_2_O and K_3_[Fe(CN)_6_]^41^. The resulting *g* = 2.3 value fits to *g* values observed by electron paramagnetic resonance spectroscopy for substituted LS manganocenes at cryogenic temperatures^34^. ^1^H and ^13^C nuclear magnetic resonance (NMR) spectroscopy in solution (298 K) (Supplementary Figs. 3–10) and single-crystal X-ray diffraction (SCXRD; 91 K, without irradiation) (Fig. 2c and Supplementary Table 1) of **1** further confirm that the LS state of **1** is stabilized over the HS state as predicted from the strong *σ*-donating character of the carbenes. The experimentally determined Mn–L bond lengths agree well with those calculated by DFT for **LS-1** (Supplementary Table 1). The infrared (IR) and Raman vibrational frequencies calculated for **LS-1** also fit very well with the experimental data (Supplementary Fig. 11 and Supplementary Tables 5 and 6), for example, the totally symmetric breathing mode (a_1_, compared with Fig. 1, *Q*_HL_(a_1_)) of **LS-1** is found at 118 cm^−1^ in the Raman spectrum and calculated at 112 cm^−1^ by DFT (scaled by 0.98). Consequently, the calculations describe the structure and flexibility of **LS-1** reasonably well. The green colour of **LS-1** arises from intense absorption bands at 431 and 590 nm, which are composed of spin-allowed metal-to-ligand charge transfer (^2^MLCT; manganese-to-pyridine) transitions according to time-dependent DFT (TD-DFT)^42,43^ calculations (Fig. 2d and Supplementary Table 7). Heating of solid **LS-1** to 515.5 K does not change its colour (Supplementary Fig. 12), confirming that thermal SCO to the HS state is absent up to this temperature, as the HS state is expected to show different absorption bands (Supplementary Table 8). This further underlines the strong destabilization of the HS state as predicted.

### Photoswitching in solution

Time-resolved absorption spectroscopy of **LS-1** was performed at 293 K in MeCN solution (Fig. 2d) to explore the excited state dynamics and the ground state recovery after light excitation. After excitation of **1** with 590-nm light, the characteristic absorption bands of the LS state diminish (ground state bleach, GSB). No excited state absorption (ESA) bands are observed on this timescale. The LS ground state recovery observed at the GSB wavelengths follows a monoexponential rate law with an unusually long lifetime *τ*_HL_(293 K) = 87 µs. For comparison, the lifetime for the HS → LS ground state recovery of LS polypyridine iron(II) complexes after light excitation is in the lower nanosecond range, between 292 and 235 K (ref. ^23^). The absence of ESA bands and the long lifetime suggest population of the HS state. TD-DFT calculations^42,43^ on geometry-optimized **HS-1** indeed predict weaker transitions in the visible spectral region (Supplementary Table 8). Consequently, an appreciable activation barrier exists that decelerates the HS → LS recovery.

This HS → LS activation barrier *E*_a,HL_ was experimentally determined by variable-temperature TA spectroscopy with *λ*_exc_ = 590 nm in MeCN between 273 and 308 K. The slope of the corresponding Arrhenius plot (Fig. 2e and equation(1)) gives *E*_a,HL_ = 0.36 eV (2,900 cm^−1^). A comparably high barrier of 0.33 eV was observed for a trigonal prismatic iron(II) complex showing a HS → LS transition associated with a coupled breathing and trigonal twisting reaction coordinate^44^.

However, the 0.36 eV barrier surpasses that of classical LS polypyridine iron(II) complexes by factors of 3.8–9.4, while the pre-exponential factor *A*_HL_ = 2.2 × 10^10^ s^−1^ is very similar to the *A*_HL_ of polypyridine iron(II) complexes^23^. The activation barriers of typical iron(II) complexes showing thermal SCO in solution are also smaller (350–1,500 cm^−1^), while the pre-exponential factors are in a similar range^27^. Extrapolation of the **HS-1** lifetime to 77 K from the Arrhenius plot gives *τ*_HL_(77 K) > 10^6^ a (Supplementary Fig. 13), which suggests potential applications in data storage using molecular **LS-1**/**HS-1** at lower temperatures.

The Arrhenius equation (equation (1)) can be related to semi-classical Marcus theory (equations (2) and (3))^23^. Assuming reasonable limiting values for the reorganization energy *λ*_HL_ (Fig. 1c), the electronic coupling constant *H*_AB_, which defines the electronic communication between the HS and LS states, can be estimated. As *H*_AB_ is proportional to *λ*_HL_^1/4^ (equation (3)), varying the reorganization energy *λ*_HL_ produces only small effects on *H*_AB_. Using *λ*_HL,lower_ = 14,100 cm^−1^ reported for the structural analogue [Fe(tpy)_2_]^2+^ (2,2′:6′,2′-terpyridine, tpy)^23^ as the lower estimate and a tripled value of *λ*_HL,upper_ = 42,300 cm^−1^ as the upper limit (based on the larger bond length changes of **1**) yields *H*_AB_ = 11–14 cm^−1^ in a narrow range. Although this electronic coupling is somewhat larger than that determined for iron(II) complexes (4.2–6.2 cm^−1^)^23^, it is still small compared with the overall barrier height *E*_a,HL_ = 2,900 cm^−1^. Considering the required double spin change Δ*S* = 2, the small electronic coupling of the HS and LS potentials is reasonable.

1 $$k=A{{\rm{e}}}^{\left(-\frac{{E}_{{\rm{a}}}}{{k}_{{\rm{B}}}T}\right)}{.}$$

2 $${E}_{{\rm{a}}}=\frac{{\left({\Delta G}_{0}+\lambda \right)}^{2}}{4\lambda }.$$

3 $$A=\frac{2{\rm{\pi }}}{\hbar }\frac{|{H}_{\mathrm{AB}}^{2}|}{\sqrt{4{\rm{\pi }}\lambda {k}_{{\rm{B}}}T}}.$$

**Fig. 3:**
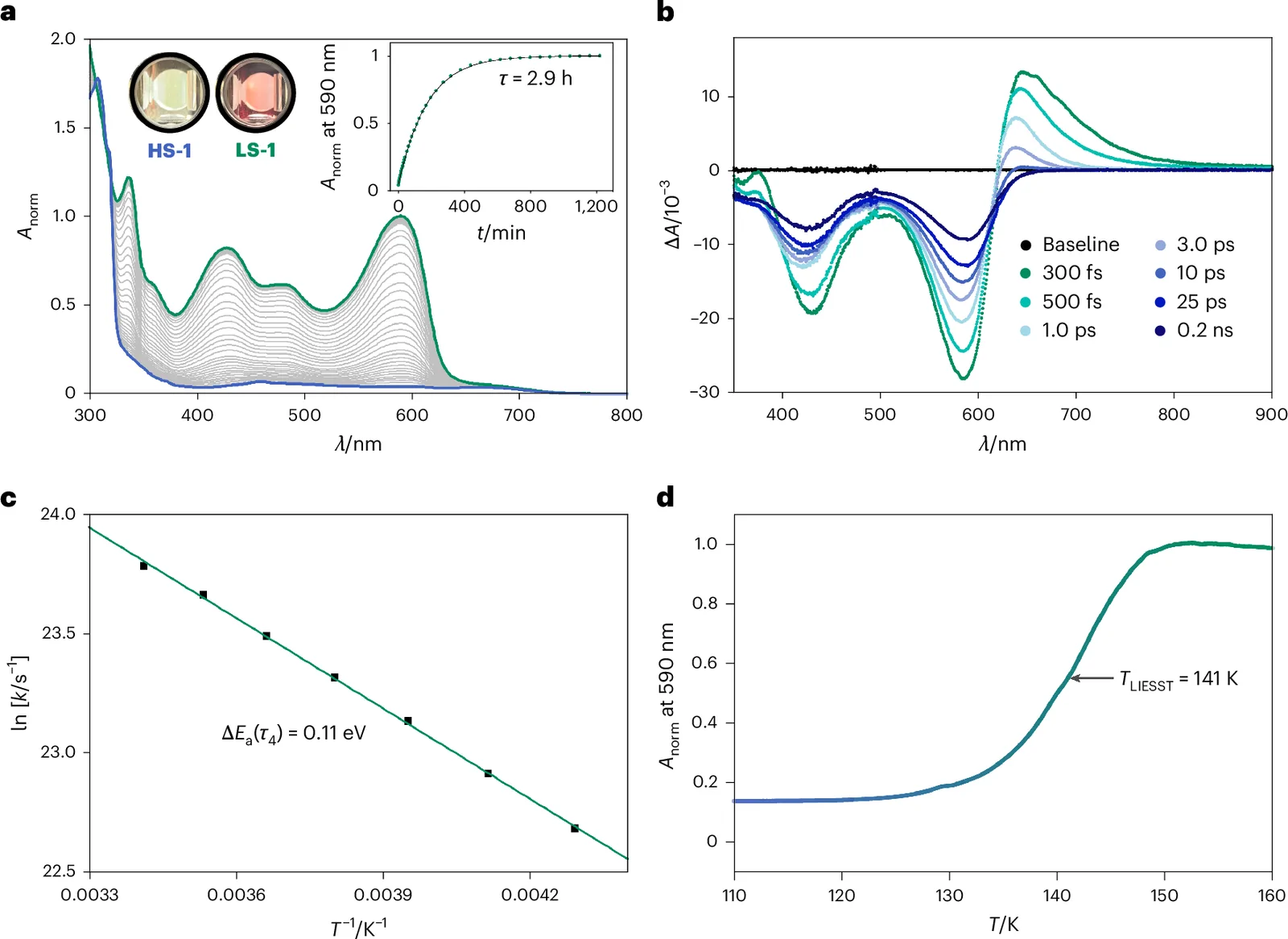
Solution state photoswitching. **a**. UV–visibile absorption spectra of a solution of **1** at 130 K in 2-MeTHF/MeCN (29:1, v/v) obtained before and after steady-state irradiation with a 560-nm LED (2 min, blue) and absorption spectra obtained at various times after switching off the light source. The final spectrum is shown in green. The inset shows the recovery of the LS state observed at *λ*_obs_ = 590 nm. The photographs show frozen solutions of **1** in 2-MeTHF/MeCN (29:1,v/v) after (**HS-1**, pale-orange–yellow) and before (**LS-1**, red) steady-state irradiation (2 min) with a 560-nm LED at 130 K. **b**, fs-TA spectra of **1** after excitation with 590 nm in MeCN at 293 K. **c**, An Arrhenius plot of **1** obtained from fs-TA spectra using excitation with 590 nm in MeCN between 233 and 293 K from temperature-dependent lifetimes *τ*_4_(*T*). **d**, The determination of *T*_LIESST_ of **HS-1** by UV–visibile–NIR absorption spectroscopy in 2-MeTHF/MeCN (29:1, v/v) solution. **HS-1** was prepared from **LS-1** at 95 K by irradiating at *λ*_exc_ = 560 nm for 2 min, the temperature was increased with a rate of 0.3 K min^−1^ and re-formation of **LS-1** was observed at *λ*_obs_ = 590 nm.

The exceptionally favourable HS lifetimes of **1** suggest continuous irradiation experiments at lower temperatures to trap the HS state (LIESST effect). Cooling a solution of **1** in 2-MeTHF/MeCN (29:1 v/v) to 95 K sharpens the UV–visible absorption bands, resulting in a noticeable colour change from green to red, but does not change the overall band pattern (Supplementary Fig. 14). Thus, **1** remains in its LS state. Irradiating a frozen solution of **LS-1** at 130 K with a 560-nm LED (Supplementary Fig. 15) for 2 min produces a colour change from red to faint orange (Fig. 3a) and decreases the ^2^MLCT absorption bands. This faint orange colour of the HS state persists for many hours at temperatures below 95 K. From the parameters derived by TA spectroscopy (Fig. 2d,e), the extrapolated HS state lifetime amounts to *τ*_HL_(77 K) > 10^6^ a (Supplementary Fig. 13). Such a huge lifetime at this temperature cannot be reliably determined experimentally within a reasonable timeframe. Performing an analogous experiment at 130 K (560-nm excitation for 2 min) also forms the orange-coloured HS complex according to UV–visible absorption spectroscopy (Fig. 3a). At this temperature, the LS complex re-forms quantitatively from the HS complex without any side reactions, as seen from the isosbestic points at 306 and 320 nm in the spectra series (Fig. 3a and Supplementary Fig. 16). The process follows an exponential rate law with *τ*_HL_(130 K, solution) of 2.9 h (Fig. 3a, inset). The combined pulsed and steady-state irradiation experiments in solution confirm ultralong lifetimes of **HS-1** prepared by LIESST of microseconds, hours and over a million years at 293 K (fluid solution), 130 K (frozen solution) and 77 K, respectively.

The ultrafast dynamics from the photoexcited LS to the HS state was probed by femtosecond (fs)-TA spectroscopy of **1** in MeCN with 430- and 590-nm light excitation (Fig. 3b and Supplementary Fig. 17). The observed dynamics are essentially independent of the excitation energy (Fig. 3b and Supplementary Figs. 18 and 19), suggesting that the initial decay after high-energy excitation is faster than the temporal resolution of our instrument. In the following, we refer to the data from excitation with 590 nm. In addition to the slow LS recovery at 293 K (*τ*_HL_ set as infinite in this ultrafast experiment), rich dynamics were observed on the (sub-)picosecond timescale with *τ*_1,2,3,4_(293 K) = 0.25, 1.0, 9.0 and 44 ps, respectively, obtained by global analysis (Supplementary Fig. 19). Clearly, a simple kinetic model involving only two excited states in addition to the LS and HS states as depicted in Fig. 1b is insufficient in this case. At 233 K, the time constants were determined as *τ*_1,2,3,4_(233 K) = 0.22, 1.1, 8.5 and 141 ps (Supplementary Fig. 20). The fitted lifetimes *τ*_1_–*τ*_3_ are independent of temperature within experimental uncertainty, while the lifetime *τ*_4_ shows a strong temperature dependence.

Variable-temperature fs-TA spectra obtained between 233 and 293 K (Supplementary Fig. 20) reveal that the state-to-state process associated with *τ*_4_ is thermally activated with a barrier of *E*_a_(*τ*_4_) = 0.11 eV (885 cm^−1^) and a pre-exponential factor *A*(*τ*_4_) = 1.60 × 10^12^ s^−1^ according to an Arrhenius analysis (equation (1) and Fig. 3c). This pre-exponential factor *A*(*τ*_4_) surpasses *A*_HL_ associated with the HS → LS relaxation with double spin change Δ*S* = 2 by two orders of magnitude, suggesting a much stronger electronic coupling. Using very large and small estimates of the reorganization energy *λ*(*τ*_4_) = 14,100–42,300 cm^−1^ yields *H*_AB_(*τ*_4_) = 90–120 cm^−1^ in a relatively narrow range (equation (3)). Hence, this thermally activated state-to-state transition associated with *τ*_4_ is an adiabatic process, for example, an ISC with Δ*S* = 1 and sufficiently strong spin–orbit coupling or an IC without spin change (Δ*S* = 0) (Supplementary Fig. 21). Using DFT (excited state) optimizations and TD-DFT methods, we were able to identify and optimize several excited states with doublet, quartet and sextet multiplicities (Supplementary Tables 1 and 2). However, their individual roles in the excited state dynamics cannot be inferred from the current experimental and computational data alone. A qualitative scheme of the excited state dynamics accounting for the identified processes and barriers is depicted in Supplementary Fig. 21. Future experiments on the ultrafast timescale using X-ray spectroscopies in solution or in the solid state to infer spin state, electronic nature and bond lengths of the various involved intermediate excited states will allow a more detailed assignment of the observed individual processes^45–47^. The quantum yield of the overall light-induced LS → HS process was estimated as approximately 33% using a spectrum of the HS state extracted from the fs-TA data (Supplementary Figs. 22 and 23). Hence, losses occur during the picosecond dynamics from the initially excited ^2^MLCT states along the way to the final HS state (Supplementary Fig. 21).

The *T*_LIESST_ value for **1** in solution was determined by absorption spectroscopy at 590 nm after conversion to the HS state by 560 nm irradiation for 2 min at 95 K. Warming to 100 K with a rate of 0.5 K min^−1^ was followed by warming at a rate of 0.3 K min^−1^ to 180 K. This procedure^17^ gave a *T*_LIESST_(solution) of 141 K (Fig. 3d). This key figure-of-merit of the manganese(II) complex **1** surpasses that of iron complexes previously reported^24,25^. The present complex class based on manganese and carbenes capable of LIESST pave the way towards switchable materials at ambient temperatures, which is challenging to achieve with iron-based complexes, which were the focus of intense SCO/LIESST research for over four decades.

### Photoswitching in the solid state

**Fig. 4:**
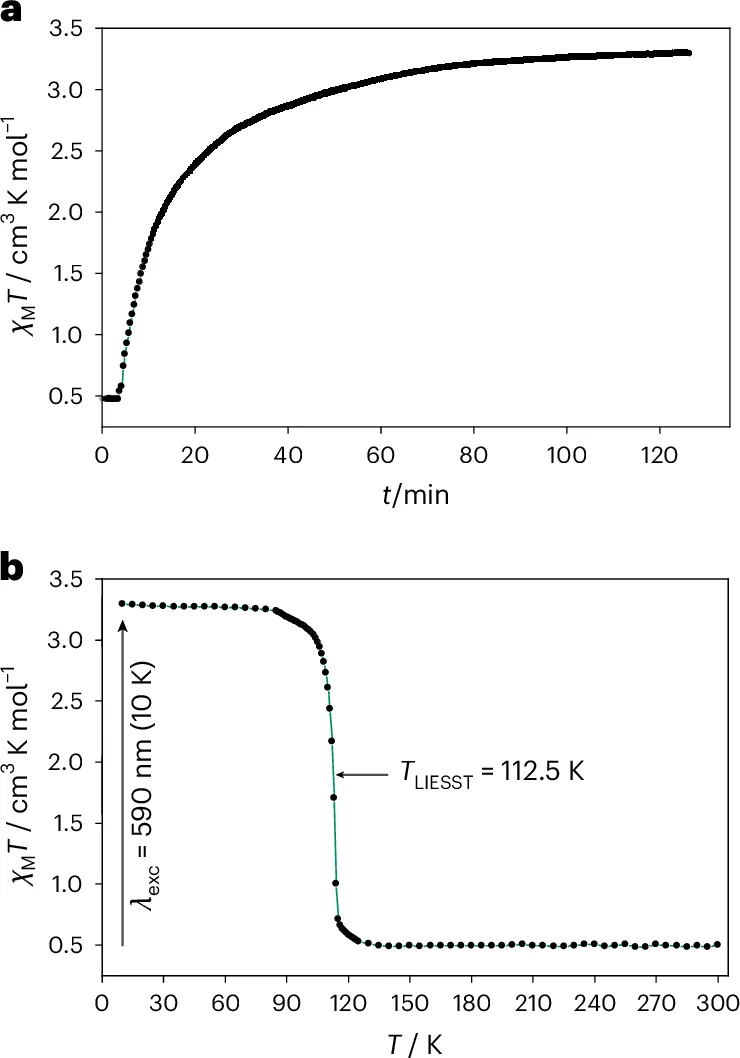
Photomagnetism of **1** in the solid state. **a**, *χ*_m_*T* versus *t* plot of solid **1** at 10 K during irradiation with 590 nm (black circles, green line as guide to the eye). **b**, *χ*_m_*T* versus *T* plot (green) of solid **1** after irradiation at 10 K for 2 h, switching off the light source and warming with 0.4 K min^−1^, giving a *T*_LIESST_ of 112.5 K.

Photoswitching of **1** via LIESST was also investigated in the solid state with microcrystalline solid material. A solid sample of **1** was cooled to 10 K in the SQUID (superconducting quantum interference device) magnetometer giving *χ*_m_*T* < 0.50 cm^3^ K mol^−1^ (Fig. 4a) confirming the presence of **LS-1**. This sample was irradiated with 590-nm light (Supplementary Fig. 15) at 10 K in the SQUID magnetometer until the magnetization was saturated (compared with 2 h in our SQUID setup) (Fig. 4a). The susceptibility temperature product reached a plateau at *χ*_m_*T* = 3.29 cm^3^ K mol^−1^, confirming the light-induced spin-state change with approximately 57% conversion (based on *g* = 2.3) in our setup. The temperature *T*_LIESST_(solid) of 112.5 K (determined with a heating rate of 0.4 K min^−1^) obtained for the solid (Fig. 4b) is lower than the value determined in solution: a *T*_LIESST_(solution) of 141 K, probably because the solid state favours the compact LS state over the expanded HS state. With a higher warming rate of 2.5 K min^−1^, the value increases to a *T*_LIESST_(solid) of 118 K (Supplementary Figs. 24 and 25). The steep relaxation curves (Fig. 4b and Supplementary Fig. 25) substantiate cooperative effects for the HS → LS relaxation in the solid state. Relaxation curves obtained after illumination at 10 K and warming to selected temperatures (95, 100, 105 and 110 K) gave the sigmoidal decays (Supplementary Fig. 26), confirming a self-accelerating process in the solid state^25^. The measured decays further substantiate that no photodamage occurs under these conditions. Indeed, several cooling–irradiation–warming cycles indicate only weak fatigue of the material (Supplementary Fig. 27).

Structural reports on the metastable HS state prepared by LIESST are restricted to iron(II) SCO complexes irradiated at *T* ≤ 40 K (refs. ^48–56^). To investigate the molecular structure of the HS state **HS-1** in the solid state, which cannot be prepared by thermal SCO (Supplementary Fig. 12), photochemical experiments were conducted in crystallo. A single crystal of **LS-1** was cooled to 91 K and irradiated with 560 nm, resulting in a colour change of the crystal to pale-orange–yellow (Supplementary Fig. 28). A collection of XRD data at *T* = 91 K confirmed the integrity of the single crystal and the preservation of the space group *P*2_1_/n. Under constant irradiation, satisfactory XRD data could be obtained for **HS-1**. Both independent cations in the asymmetric unit display changes in geometry as compared with those of the LS structure, indicative of the HS state (Supplementary Table 1). The average Mn–C/Mn–N bond lengths of **1** at 91 K increased from 1.995(1)/1.955(2) Å (**LS-1**) to 2.277(2)/2.332(2) Å (**HS-1**), respectively (Fig. 2c). These bond length changes observed in the solid state agree well with the computationally predicted ones modelled in a continuum solvent and confirm that the HS state is strongly expanded (Fig. 2b,c and Supplementary Table 1). The calculated differences of Δ*d*_MnC,DFT_ = 0.279 Å and Δ*d*_MnN,DFT_ = 0.401 Å reproduce the average differences Δ*d*_MnC,exp_ = 0.282 ± 0.002 Å and Δ*d*_MnN,exp_ = 0.377 ± 0.003 Å measured in the solid state very well. This fully supports our design concept that larger zero-point energy differences, Δ*E*^0^_HL_, together with larger distortions, Δ*Q*_HL_, can achieve LIESST behaviour at higher temperatures (Fig. 1c).

**Fig. 5:**
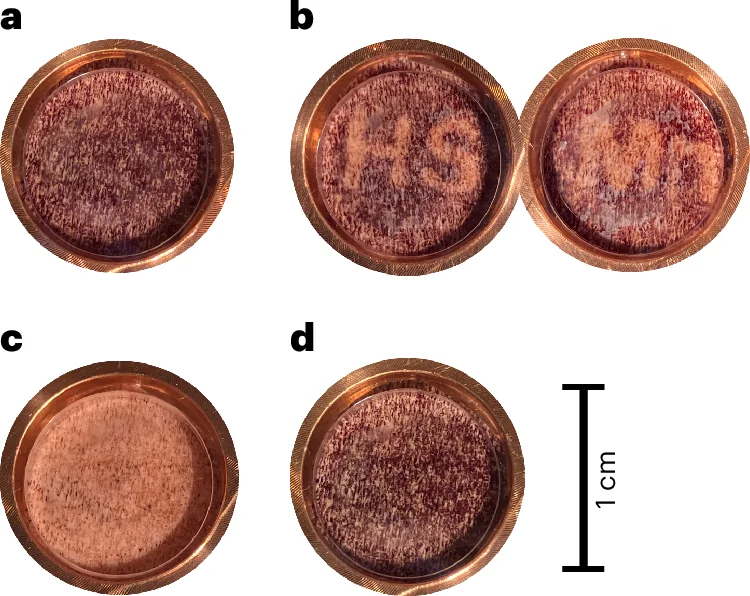
Writing with light. **a**, A photograph of a paper disc with **LS-1** cooled to 77 K in a cryostat. **b**, Photographs of the paper after irradiation with a 560-nm LED through masks with the pattern ‘HS’ and ‘Mn’, respectively, at 77 K for approximately 2 min (spatially controlled **HS-1** formation). **c**, A photograph of the paper after irradiation of the patterned disc with a white light torch to clear the pattern (full conversion to **HS-1**) (Supplementary Video 1). **d**, A photograph of the paper after warming of the patterned disc to clear the pattern (full conversion to **LS-1**). **LS-1** appears brownish at low temperatures, which is a combination of slight band shifts and band narrowing at lower temperature (Supplementary Fig. 14), which leads to this colour perception by the human eye.

To probe the material properties of **1** in a test read/write application, a powdery sample of **LS-1** was sprinkled and pressed on a small piece of paper by a spatula. This paper disc was covered with 2-methylpentane to ensure homogeneous heat transport to the paper disc and cooled to 77 K in a cryostat (Fig. 5a). Irradiating the paper at 77 K with 560-nm light through masks writes the corresponding patterns ‘HS’ and ‘Mn’ onto the disc (Fig. 5b). At this temperature, the pattern is essentially indefinitely stable. The pattern can be erased either by irradiating without the mask to form the HS complex (Fig. 5c and Supplementary Video 1) or by warming to form the LS complex (Fig. 5d). The combined experiments demonstrate that **1** can store binary information (‘0’ and ‘1’ bits) that can be written with visible light.

## Conclusion

LIESST was restricted to iron(II) and iron(III) complexes, with the highest *T*_LIESST_ value reported in 2001 as 132 K. Now, a carefully designed LS carbene manganese(II) complex **1** with covalent metal–ligand bonding and a strongly distorted high-energy HS state exhibits LIESST in solution and in the solid state. In solution, the HS state forms quantitatively under visible light irradiation. The extrapolated lifetime of the HS state at 77 K amounts to over 1 million years. The *T*_LIESST_ value of 141 K exceeds previously reported *T*_LIESST_ values of iron complexes. The photomagnetic effect even persists at room temperature with a microsecond lifetime. The principles uncovered in the present study unlock opportunities for achieving stable and robust spin-state switching without bond dissociation, exploiting the LIESST effect under ambient conditions using metal ions beyond iron, paving the way for device applications^57,58^ and single molecule information storage^59^.

## Methods

### General synthetic procedures

[H_2_pbmi]Br_2_, MnBr_2_ and ferrocenium hexafluorophosphate were purchased from TCI, Sigma-Aldrich and BLDpharm, respectively. CH_3_CN/CD_3_CN, diethylether, toluene and THF were distilled from CaH_2_, sodium and potassium, respectively. Dry CH_2_Cl_2_ and 2-MeTHF were purchased (AcroSealTM) and degassed by bubbling argon through the solvent before use. THF was stored over pre-activated 3 Å molecular sieves. Other reagents were used as received from commercial suppliers (Sigma-Aldrich, Fisher Sci., TCI and Acros). All reactions and measurements were performed under a water-free argon atmosphere if not stated otherwise. A glovebox (UniLab/Mbraun: Ar 5.0, O_2_ < 0.1 ppm, H_2_O < 0.1 ppm) was used to store and weigh sensitive compounds for synthesis, as well as to prepare samples for spectroscopic and analytical measurements.

### Elemental analysis

Elemental analysis was performed by the Mikroanalytisches Labor Kolbe, c/o Fraunhofer Institut UMSICHT, Oberhausen, Germany.

### NMR spectra

**Extended Data Fig. 1:**
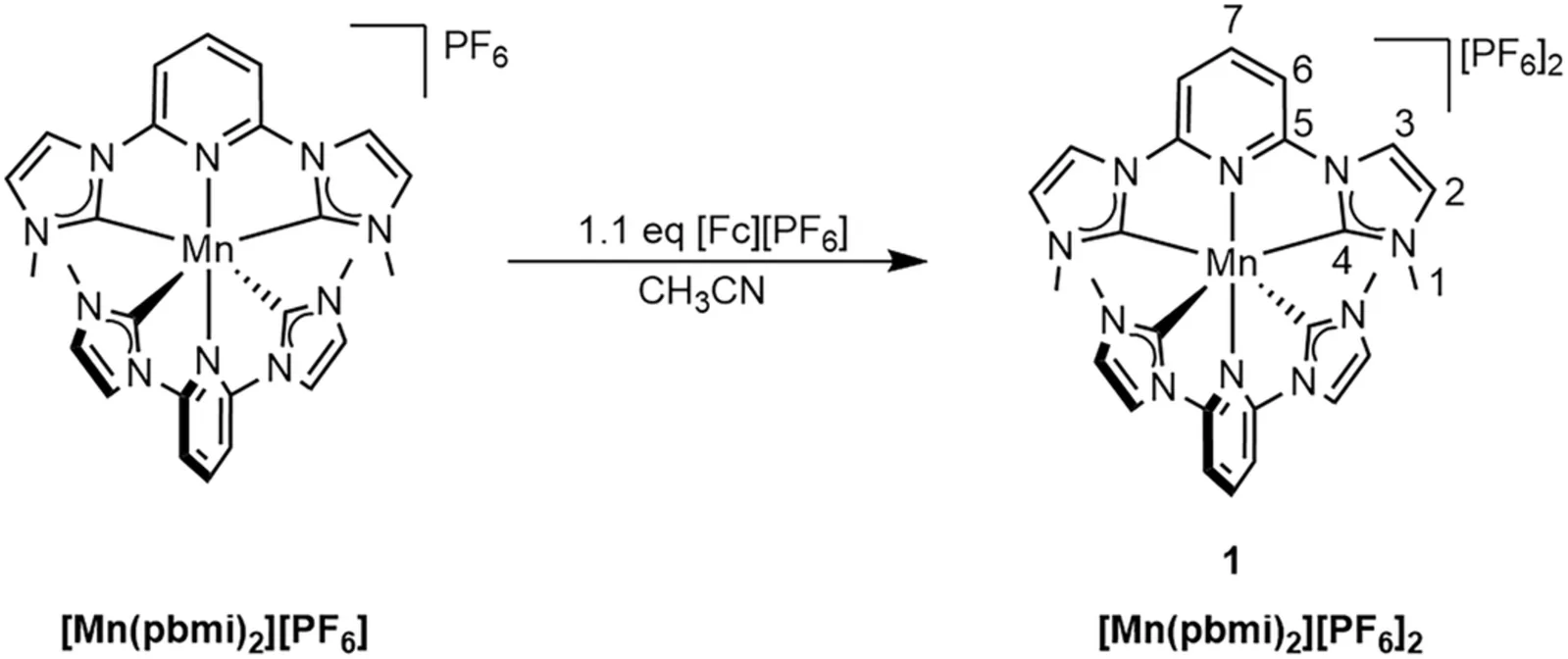
Chemical structure of [Mn(pbmi)_2_][PF_6_] and synthesis of **1**, [Mn(pbmi)_*2*_][PF_*6*_]_2_. The numbers on the right structure are used for the ^1^H and ^13^C-NMR assignments, see methods section.

NMR spectra were recorded on a Bruker Avance NEO or a Bruker Avance DRX 400 spectrometer at 400 MHz (^1^H), 100 MHz (^13^C{^1^H}) and 376.5 MHz (^19^F). Resonances for ^1^H and ^13^C{^1^H} are reported in parts per million versus the solvent signal as an internal standard (CD_3_CN (^1^H *δ* = 1.94 ppm and ^13^C{^1^H} *δ* = 1.3 ppm, *δ* = 118.3 ppm))^60^. Resonances for ^19^F nuclei are reported relative to C(^35^Cl)_2_(^37^Cl)F (*δ* = 0 ppm). Calibration of the spectrometer before the measurement was performed with α,α,α-trifluorotoluene in CDCl_3_ (*δ* = −63.9 ppm). Multiplicities are abbreviated as follows: s, singlet; d, doublet; t, triplet; dd, doublet of doublet. See Extended Data Fig. 1 for the ^1^H and ^13^C-NMR assignment.

### IR spectra

IR spectra were recorded on an Agilent Cary 630 FTIR spectrometer with an ATR (attenuated total reflection) unit containing a diamond crystal inside an argon-filled glovebox. The intensities are qualitatively indicated as weak (w), medium (m), strong (s) and very strong (vs).

### Raman spectra

Raman spectra (*λ*_exc_ = 1064 nm) were measured on a Nicolet 5700 FTIR spectrometer combined with a NXR 9650 FT-Raman module equipped with a 1,064-nm laser (laser power 20–1,500 mW; resolution 2 cm^−1^), a microstage microscope and an NXR Genie Ge-detector using single crystals or crystalline powders in glass capillaries (under inert gas). The intensities are qualitatively indicated as weak (w), medium (m), strong (s) and very strong (vs).

### ESI^+^ mass spectra

ESI^+^ mass spectra were recorded on an Agilent 6545 QTOF-MS spectrometer.

### UV–visible–NIR absorption spectra

UV–visible–near infrared (NIR) absorption spectra were recorded on an Agilent Cary 5000 spectrometer using 1.00-cm quartz cells (Starna) equipped with Schott valves. Measurements at low temperatures were carried out using a liquid nitrogen-cooled cryostat Optistat DN from Oxford Instruments equipped with a low temperature 1-cm quartz cuvette (Hellma). Baseline corrections for low temperature measurements were performed for all temperatures. Shoulders are abbreviated as sh.

### HS-1 to LS-1 decay at 130 K via UV–visible–NIR absorption spectroscopy

A 0.13 mM solution of **1** in 2-MeTHF/MeCN (29:1, v/v) was cooled to 130 K, followed by irradiation at *λ*_exc_ = 560 nm with a high-power LED from Prizmatix (UHP-T-560-DI; output power 2.6 W) (Supplementary Fig. 15) for 2 min to ensure full **LS-1** to **HS-1** conversion (Fig. 3a). UV–visible–NIR absorption spectra were measured over time to observe the conversion from **HS-1** to **LS-1**. The evolution trace (Fig. 3a) at *λ*_obs_ = 590 nm was fitted with a monoexponential function.

### *T*_LIESST_ experiment via UV–visible–NIR absorption spectroscopy

A 0.14 mM solution of **1** in 2-MeTHF/MeCN (29:1, v/v) was cooled to 95 K (Supplementary Fig. 14), followed by irradiation at *λ*_exc_ = 560 nm with a high-power LED from Prizmatix (UHP-T-560-DI; output power 2.6 W) (Supplementary Fig. 15) for 2 min to ensure full **LS-1** to **HS-1** conversion. The temperature was then increased to 100 K (0.5 K min^−1^) and kept at 100 K for 10 min. With a rate of 0.3 K min^−1^, the temperature was increased to 180 K. The conversion from **HS-1** to **LS-1** was observed by following the absorbance at *λ*_obs_ = 590 nm (Fig. 3d). The full UV–visible-NIR absorption spectrum (Supplementary Fig. 14) after the *T*_LIESST_ experiment was measured at 293 K.

### Solid state spin-state switching

A crystalline, powdery sample of **1** was sprinkled over a piece of paper. The powder was pressed with a spatula on the paper to cover the paper with the sample. A round disc of 1-cm diameter was cut out from the paper. The disc was placed in a MicrostatN cryostat from Oxford Instruments. To ensure thermal conductivity, the disc was covered with 2-methylpentane before cooling to 77 K. The disc was irradiated at 77 K with either a *λ*_exc_ = 560 nm high-power LED from Prizmatix (UHP-T-560-DI; output power 2.6 W) (Supplementary Fig. 15) or with a white light Seeker 4 mini torch from Olight, which leads to the formation of the HS state. The HS state was erased by increasing the temperature to *T* > 130 K leading to recovery of the LS state.

### Thermal SCO

Crystalline powder of **1** was filled into a glass capillary under inert atmosphere, followed by an increase in temperature in a melting point apparatus from Gallenkamp until 540.5 K. No colour change was observed upon temperature increase, although the dark solid showed signs of decomposition at *T* > 515 K. Consequently, **1** shows no thermal SCO from LS to HS under these conditions.

### SCXRD

Intensity data for crystal structure determination were collected with a STOE STADIVARI diffractometer from STOE and CIE GmbH with an Oxford Cryostream 700 using Mo-K_α_ radiation (*λ* = 0.71073 Å). The diffraction frames were integrated using the STOE X-Area^61^ software package and were corrected for absorption with STOE LANA^62,63^ of the STOE X-Area software package by scaling of reflection intensities followed by a spherical absorption correction. The structures were solved with SHELXT^64^ and refined by the full-matrix method based on *F*^2^ using SHELXL^65^ of the SHELX^66^ software package and the ShelXle^67^ graphical interface. All non-hydrogen atoms were refined anisotropically, while the positions of all hydrogen atoms were generated with appropriate geometric constraints and allowed to ride on their respective parent atoms with fixed isotropic thermal parameters. Co-crystalized acetonitrile has been constructed with the DSR plugin^68^ in ShelXle^67^_._

The irradiation experiment on the X-ray diffractometer was carried out by exposing a single crystal (which was kept in a Parabar 10312 cryoprotection oil from Hampton research) at 91 K to a *λ*_exc_ = 560 nm LED (Prizmatix UHP-T-560-DI; output power 0.5 W). A colour change from dark red to pale orange was observed within 10 min of irradiation (Supplementary Fig. 28). To ensure complete **LS-1** → **HS-1** conversion before data collection, the XRD measurement was initiated after 2 h of continuous irradiation. The crystal was kept under constant irradiation throughout the data collection. After completion of the XRD experiment, the light source was switched off and a photograph was immediately taken, which still showed the characteristic pale-orange colour of the **HS-1** state. Subsequently, the sample was allowed to warm to 293 K, resulting in an immediate conversion to the dark-coloured **LS-1** complex. After re-cooling to 91 K, a final photograph of the crystal of **LS-1** was recorded (Supplementary Fig. 28).

### SQUID measurements

Sample preparation for magnetic studies was carried out under inert atmosphere in a glovebox under Ar. Magnetic susceptibility measurements were performed with a Quantum Design MPMS3-EC SQUID magnetometer in direct current and settle mode on powdered microcrystalline samples under an applied magnetic field of 1 T.

Variable temperature susceptibility data were collected in a temperature range of 2–300 K. The powdered microcrystalline sample (11 mg) was placed in a gelatine capsule mounted on a plastic straw. The temperature-dependent magnetic contribution of the holder (capsule and straw) was experimentally determined. The obtained raw voltages of the sample and background measurements were subtracted from each other with the help of the SquidLab^69^ routine for MATLAB^70^. Fitting of the corrected raw voltages gave the raw susceptibility data, which were corrected for the underlying diamagnetism using Pascal’s constants^71^.

For the photomagnetic experiments, approximately 0.4 mg of powdered microcrystalline sample was placed into the quartz-assembly of the magneto-optic-holder (Quantum Design) equipped with a Mountain Photonics Hyperchromator light source (Supplementary Fig. 15). Background treatment was done analogously, as described above. To determine precisely the amount of sample used in the photomagnetic setup, variable-temperature susceptibility data were collected from 300 to 10 K without irradiation and compared with the previously obtained susceptibility data of the standard measurement. The actual sample mass was determined to be 0.47 mg and 0.40 mg, respectively, to obtain the susceptibility values of the standard measurements.

Irradiation experiments in the SQUID magnetometer with a photomagnetic setup were performed at *λ*_exc_ = 590 nm (Mountain Photonics Hyperchromator, 6 nm full width at half maximum) (Supplementary Fig. 15) at 10 K for 2 h and monitored by continuous DC measurements. The coupled light causes a small heat input and leads to a small deviation between the actual temperature at the sample and the measured temperature by the thermometers located at the chamber wall. The temperature at the sample was corrected by comparing the susceptibility values after 2 h, while irradiating and immediately after switching off the light source. The temperature deviation between sample and thermometers was determined to be approximately 1.6 K, and the temperature on the sample during irradiation was corrected for each irradiation experiment in the range of 11.5–11.7 K. After the sample (0.47 mg) was irradiated for 2 h, variable-temperature susceptibility data were collected in the temperature range 10–300 K with a heating rate of 2.5 K min^−1^ in DC settle mode. The experiment was repeated with less sample (0.40 mg) and a slower heating rate of 0.4 K min^−1^.

Relaxation studies were conducted with the photomagnetic setup. After irradiating for 2 h at around 10 K (see above) using a *λ*_exc_ = 590 nm excitation, the light source was switched off and the sample was heated with the maximum rate of 30 K min^−1^ to the desired temperature of 90 K, 95 K, 100 K, 105 K and 110 K, respectively. After stabilization of the temperature, continuous DC measurements were done until the magnetic moment reached a plateau. Thereafter, the sample was heated to 300 K before the next experiment to avoid any remanent effects. The temperature at which the relaxation studies was performed was altered in the following order: 100 K, 110 K, 95 K, 105 K and 100 K to avoid any undesired effects. At room temperature, a *χ*_m_*T*-value of 0.50 cm^3^ K mol^−1^ is observed, which slightly exceeds the spin-only value of 0.38 cm^3^ K mol^−1^ for one *S* = 1/2. The value remains nearly unchanged upon cooling. Only at low temperature a small decrease is observed, which can be due to the saturation of magnetization, spin–orbit coupling and/or small intermolecular exchange interactions. The *χ*_M_*T* versus *T* curve can be simulated satisfactorily by applying a *g* value of 2.30.

### fs-TA experiments

fs-TA experiments were conducted using a Helios pump–probe setup from Ultrafast Systems paired with a regeneratively amplified 1,030-nm laser (Pharos, Light Conversion; 1,030 nm, <175 fs, 2 mJ). The effective laser repetition rate of 2 kHz was set via an internal pulse picker. A small portion of the 1,030-nm fundamental was directed to the optical delay line and was subsequently used to generate broadband probe light by focusing the beam onto a sapphire for measurements in the visible-NIR range (450–915 nm). In the UV–visible spectral range (330–500 nm), the second harmonic was focused onto a second sapphire instead of the fundamental. The pump pulse was generated with an optical parametric amplifier (Apollo Y, Ultrafast Systems) and the beam diameter at the sample was adjusted to 100–150 µm at the sample to assure homogeneous excitation of the observation volume, which is defined by the probe diameter (approximately 25 µm). The sample solutions were measured under argon atmosphere in a 1-mm quartz cuvette. To generate spectra that cover the whole spectral region from 350 nm to 915 nm, the UV–visible and visible-NIR part of the TA spectra were recorded separately under identical conditions and were combined by matching the relative band maxima of both spectral regions with the corresponding TA spectra providing spectra at a 1-ns delay time. For the latter, the entire spectral range can be measured simultaneously. Preprocessing of the data, including chirp and baseline correction, has been performed using the Surface Xplorer 4.3.0 software from Ultrafast Systems. The open source Python-based data analysis tool KiMoPack 7.4.9^72^ was employed for global analysis of the TA data. For the global fit, data points before 350 fs were cut-off to avoid additional components due to coherent artefacts.

### LIESST quantum yield

The LIESST quantum yield was obtained from the ratio (Supplementary Fig. 23) of the bleaching (at 570 nm) after full conversion to **HS-1** (300 ps after excitation) and the initial bleaching (200 fs upon excitation). In the investigated system, negative signals are exclusively caused by depopulation of the ground state (GSB). Hence, the loss of the negative signal is directly related to the ground state recovery. However, this value must be considered as an upper limit for the LIESST quantum yield, as the bleaching is superimposed in the TA spectrum with ESA. Although we know from the experiment ‘**HS-1** to **LS-1** decay at 130 K via UV–visible-NIR absorption spectroscopy’ (vide supra) that **HS-1** shows only negligible absorption features in the visible spectral region, we cannot exclude that the initially populated ^2^MLCT state possesses a more pronounced ESA, reducing the observed bleaching, which in turn would lead to an underestimation of the initial depopulation of the ground state.

### TA spectroscopy

TA spectroscopy experiments were carried out using a modified version of the described fs-TA spectroscopy setup. For this purpose, the Eos add-on has been employed, which uses a photonic crystal fibre-based supercontinuum laser as the probe light source. In contrast to the femtosecond measurements, the pump–probe time delay is controlled electronically with a time resolution of <1 ns. The effective laser repetition rate was set to 1 kHz. Fitting and averaging of the nanosecond decay traces and averaging of the TA spectra was performed with Mathematica 14.3.

### Variable temperature TA measurements

Variable temperature TA measurements were carried under nitrogen atmosphere in an Optistat DN cryostat from Oxford Instruments. The TA setup was employed as described for the room temperature measurements (fs- and ns-TA experiments). The effective laser repetition rate was set to 1 kHz for all TA spectroscopy experiments.

### DFT calculations

DFT calculations were carried out using the ORCA program package^73^ (version 5.0.3). Geometry optimizations were performed by using unrestricted Kohn–Sham orbital DFT and tight convergence criteria (keywords ‘tightscf’ and ‘tightopt’). All calculations were performed using the B3LYP functional^74–76^ using Ahlrichs’ polarized valence triple-zeta basis set (def2-TZVP)^77,78^ employing the RIJCOSX approximation (keyword ‘RIJCOSX’)^79,80^ and the auxiliary basis set (SARC/J)^81^. Relativistic effects were calculated at the zeroth order regular approximation (keyword ‘ZORA’) level^77^ using relativistically adjusted basis sets. To account for solvent effects, a conductor-like screening model (keyword ‘CPCM’) modelling MeCN was used in all calculations^82,83^. Atom-pairwise dispersion correction was performed with the Becke–Johnson damping scheme (keyword ‘D3BJ’)^84,85^. Explicit counter ions and/or solvent molecules were not taken into account. The ^4^MLCT state was geometry optimized using def2-SVP^74^ as a basis set and the ^4^MC starting geometry with contracted Mn–N_py(1,2)_ bond lengths of 1.5 Å. A numerical frequency calculation confirmed that the optimized geometry corresponds to a minimum structure on this level of theory. Increasing the basis set to def2-TZVP followed by optimization of the def2-SVP-optimized geometry converged to the ^4^MC state, so that the ^4^MLCT state was not accessible with the larger basis set, even with reduced step sizes. The excited doublet and sextet MLCT states ^2^MLCT(3) and ^6^MLCT(1) (keywords ‘iroot 3’ and ‘iroot 1’, respectively) were geometry optimized using the number of roots keyword ‘nroots 7’ (^2^MLCT(3)) and ‘nroots 5’ (^6^MLCT(1)) respectively, following the root (keyword ‘followiroot true’) in combination with the same configuration as above (keywords ‘tightopt’, ‘tightscf’, ‘RIJCOSX’, ‘CPCM(acetonitrile)’, ‘ZORA’ and ‘D3BJ’). The ^6^MLCT(1) geometry optimization was based on TD-DFT calculations from the **HS-1** geometry, whereas the ^2^MLCT(3) geometry optimization was based on the TD-DFT calculations from the **LS-1** geometry. TD-DFT calculations were performed using the TDA approximation, which is set as a default in ORCA 5.0.3. Numerical frequency calculations using the same keywords (‘iroot n’, ‘nroot n’ and ‘followiroot true’) as in the geometry optimizations confirmed the minimum structures, respectively. The reported calculated IR and Raman frequencies were obtained from a numerical frequency calculation with the keyword ‘elprop Polar 1’ without a solvent model. The resulting frequencies were scaled by a factor of 0.98. The charge transfer number analyses of the fifty TD-DFT-calculated transitions (keyword ‘nroots 50’) were done using TheoDORE 3.2^86–88^. The reported calculated transition energies are shifted by 0.4 eV and 0.1 eV to lower energies to match the experimental absorption spectra for **LS-1** and **HS-1**, respectively (Supplementary Tables 7 and 8). For charge transfer number analysis, the respective complex cation was divided into three fragments: the manganese centre, the two pyridines and the four carbenes.

### Calculation of prediction intervals

The 95% prediction interval (PI) of the HS lifetime extrapolated with the Arrhenius fit to 77 K (Supplementary Fig. 13) was calculated using equation (4) with ${q}_{0.975}^{{t}_{n-2}}$ and *n* being the quantile of the *t*-distribution and the number of data points, respectively.

4 $${\mathrm{PI}}_{0.95}=\hat{\alpha }+\hat{\beta }{x}_{0}\pm {\hat{\sigma } q}_{0.975}^{{t}_{n-2}}\sqrt{1+\frac{1}{n}+\frac{{\left({x}_{0}-\bar{x}\right)}^{2}}{{\sum }_{i=1}^{n}{\left({x}_{i}-\bar{x}\right)}^{2}}}.$$

$\hat{\alpha }$ and $\hat{\beta }$ denote the intercept and the slope of the linear fit function, respectively. ${x}_{i}$, ${x}_{0}$ and $\bar{x}$ are the position of individual data points, the position of the prediction and the mean value of the positions of the data set.

### Counterion exchange from [H_2_pbmi]Br_2_ to [H_2_pbmi][PF_6_]_2_

1,1′-(Pyridine-2,6-diyl)bis(1-methylimidazolium) dibromide (2 g, 5 mmol, 1 eq.) was dissolved in H_2_O (20 ml), followed by addition of KPF_6_ (9.2 g, 50 mmol, 10 eq.) as a solid. The suspension was stirred for 30 min at room temperature giving a colourless precipitate. After filtration, the colourless solid was dried under reduced pressure to yield [H_2_pbmi][PF_6_]_2_ (2.2 g, 4.1 mmol, 83%) as a colourless solid.

^1^H NMR (400 MHz, CD_3_CN, 298 K): *δ*/ppm = 9.42 (dd, 2H, H^4^), 8.43 (t, ^3^*J*_HH_ = 8.2 Hz, 1H, H^7^), 8.19 (dd, 2H, H^2/3^), 7.90 (d, ^3^*J*_HH_ = 8.2 Hz, 2H, H^6^), 7.62 (dd, 2H, H^2/3^), 4.01 (s, 6H, H^1^).

^19^F NMR (376.5 MHz, CD_3_CN, 298 K): *δ*/ppm = −73.71 (s, ^1^*J*_FP_ = 707 Hz, PF_6_).

### Synthesis of [Mn^I^(pbmi)_2_][PF_6_]

A dry and argon-purged 50-ml Schlenk flask equipped with a stirring bar was charged with [H_2_pbmi][PF_6_]_2_ (700 mg, 1.32 mmol, 2.5 eq.). Dry THF (30 ml) was added under argon. The suspension was cooled to 195 K, followed by dropwise addition of sodium bis(trimethylsilyl)amide (1 M in THF, 2.64 ml, 5 eq.). The reaction mixture was stirred at this temperature for 30 min. After addition of a suspension of MnBr_2_ (142 mg, 0.661 mmol, 1 eq.) in THF (10 ml), the cooling bath was removed and the solution was allowed to slowly reach room temperature. The dark-purple reaction mixture was stirred at room temperature for 16 h in the dark, followed by filtration of the solution. After removing the solvent under reduced pressure, the crude product was dissolved in dichloromethane (20 ml), filtered and crystallized by addition of toluene (30 ml) at 255 K to form dark-purple needles of [Mn^I^(pbmi)_2_][PF_6_] (211 mg, 0.311 mmol, 47%).

^1^H NMR (400 MHz, CD_3_CN, 298 K): *δ*/ppm = 7.47 (d, ^3^*J*_HH_ = 7.8 Hz, 4H, H^6^), 7.10 (s, 4H, H^2/3^), 6.96 (t, ^3^*J*_HH_ = 7.8 Hz, 2H, H^7^), 6.59 (d, ^3^*J*_HH_ = 2.1 Hz, 4H, H^2/3^), 2.97 (s, 12H, H^1^).

^19^F NMR (376.5 MHz, CD_3_CN, 298 K): *δ*/ppm = –74.00 (s, ^1^*J*_FP_ = 706 Hz, PF_6_).

### Synthesis of [Mn^II^(pbmi)_2_][PF_6_]_2_ (LS-1)

[Mn^I^(pbmi)_2_][PF_6_] (51.1 mg, 0.0753 mmol, 1 eq.) and ferrocenium hexafluorophosphate (27.3 mg, 0.0825 mmol, 1.1 eq.) were dissolved in 5 ml and 2 ml MeCN, respectively. The ferrocenium hexafluorophosphate solution was added dropwise to the [Mn^I^(pbmi)_2_][PF_6_] solution under constant stirring. This was accompanied by a colour change from dark purple to dark green. The reaction mixture was stirred for 30 min, followed by removal of MeCN under reduced pressure. The crude product was washed with diethylether (3 × 3 ml) and dried under reduced pressure. Dichloromethane (7 ml) was added, the solution was filtered and the dichloromethane was removed under reduced pressure. The product was crystallized by dissolving the crude product in MeCN (4 ml) and addition of toluene (10 ml) by layer diffusion to obtain **1** [Mn^II^(pbmi)_2_][PF_6_]_2_ as dark-green crystals (25 mg, 0.030 mmol, 44%) suitable for SCXRD. See Extended Data Fig. 1 for a scheme of the synthetic procedure.

Elemental analysis of dried **LS-1**: observed C 37.80, H 3.20, N 16.96; calculated for C_26_H_26_F_12_MnN_10_P_2_, C 37.93, H 3.18, N 17.01.

HR-ESI^+^ (MeCN): *m/z* (%) = 266.5855 (100, [Mn(pbmi)_2_]^2+^), 678.1359 (58.5, {[Mn(pbmi)_2_][PF_6_]}^+^).

UV–visible–NIR (MeCN, 293 K): *λ*_max_/nm (*ε*/10^3^ M^−1^ cm^−1^) = 590 (7.8), 483 (4.7, sh), 431 (6.9), 362 (5.2, sh), 338 (9.0), 308 (12.4, sh), 288 (18.7), 241 (32.5).

UV–visible–NIR (2-MeTHF/MeCN (29:1), 293 K): *λ*_max_/nm (*ε*/10^3^ M^−1^ cm^−1^) = 592 (7.1), 484 (4.5, sh), 432 (6.3), 365 (4.9, sh), 338 (8.7).

UV–visible–NIR (2-MeTHF/MeCN (29:1), 95 K): *λ*_max_/nm (*ε*/10^3^ M^−1^ cm^−1^) = 589 (9.2), 480 (5.7), 427 (7.8), 359 (5.4, sh), 337 (11.3).

^1^H NMR (400 MHz, CD_3_CN, 298 K): *δ*/ppm = 10.90 (s, 12H, H^1^), 6.33 (d, ^3^*J*_HH_ = 7.4 Hz, 4H, H^6^), 4.80 (s, 4H, H^2/3^), 2.06 (t, ^3^*J*_HH_ = 7.2 Hz, 2H, H^7^), –1.41 (s, 4H, H^2/3^).

^13^C NMR (100 MHz, CD_3_CN, 298 K): *δ*/ppm = 222.3 (s, C^7^), 108.6 (s, C^2/3^), 38.5 (s, C^2/3^), 27.2 (s, C^6^), 18.7 (s, C^1^).

ATR-IR: $\widetilde{\upsilon }$/cm^−1^ = 3,179 (w), 3,151 (w), 3,113 (w), 2,951 (w), 1,619 (m), 1,576 (m), 1,558 (m), 1,489 (s), 1,472 (s), 1,451 (s), 1,405 (s), 1,340 (m), 1,312 (m), 1,289 (m), 1,265 (s), 1,241 (s), 1,185 (m), 1,148 (m), 1,101 (m), 1,082 (m), 997 (m), 954 (m), 877 (m), 818 (vs, PF_6_), 777 (vs), 726 (s), 710 (s), 676 (vs), 618 (m), 600 (m), 579 (m), 553 (vs, PF_6_), 500 (m), 466 (m), 447 (m).

Raman: $\widetilde{\upsilon }$ */* cm^−1^ = 3,179 (w), 2,954 (w), 1,609 (s), 1,567 (m), 1,476 (m), 1,441 (w), 1,411 (m), 1,392 (m), 1,342 (w), 1,288 (w), 1,271 (w), 1,241 (m), 1,183 (w), 1,097 (vs), 1,017 (vs), 1,000 (s), 739 (m, PF_6_), 649 (s), 378 (s), 363 (w), 333 (w), 167 (m), 118 (m).

Crystallographic data of [Mn(pbmi)_2_][PF_6_]_2_ (**LS-1**) without irradiation. C_26.25_H_26.38_F_12_MnN_10.12_P_2_ (828.58); monoclinic; *P*2_1_/n; *a* = 19.7708(4) Å, *b* = 11.3604(3) Å, *c* = 28.7772(5) Å, *β* = 99.0560(14)°; *V* = 6382.9(2) Å^3^; *Z* = 8; density (calculated) 1.724 g cm^−3^, *T* = 91(2) K, *µ* = 0.624 mm^−1^, F(000) = 3342; crystal size 0.262 × 0.136 × 0.040 mm^3^; *θ* = 2.237–27.000 °; −25 ≤ *h* ≤ 25, −14 ≤ *k* ≤ 14, −36 ≤ *l* ≤ 32; rfln collected 39,296; rfln unique 13,850 [*R*(int) = 0.0315]; completeness to *θ* = 27.000 ° of 99.3%; semi-empirical absorption correction from equivalents; max. and min. transmission 0.9537 and 0.5271; data 13,850; restraints 591, parameters 1120; goodness-of-fit on *F*^2^ = 1.058, final indices [*I* > 2*σ* (*I*)] *R*_1_ = 0.0460, w*R*_2_ = 0.1138; *R* indices (all data) *R*_1_ = 0.0700, w*R*_2_ = 0.1191; largest diff. peak and hole 0.866 and −0.605 e Å^−3^.

Crystallographic data of [Mn(pbmi)_2_][PF_6_]_2_ (**HS-1**) after irradiating **LS-1** at *λ*_exc_ = 560 nm. C_26.25_H_26.38_F_12_MnN_10.12_P_2_ (828.58); monoclinic; *P*2_1_/n; *a* = 20.3239(9) Å, *b* = 11.7142(3) Å, *c* = 28.7749(12) Å, *β* = 100.546(3)°; *V* = 6735.0(4) Å^3^; *Z* = 8; density (calculated) 1.634 g cm^−3^, *T* = 91(2) K, *µ* = 0.591 mm^−1^, F(000) = 3342; crystal size 0.229 × 0.135 × 0.076 mm^3^; *θ* = 2.552 to 24.999 °; −24 ≤ *h* ≤ 23, −13 ≤ *k* ≤ 13, −34 ≤ *l* ≤ 33; rfln collected 36,758; rfln unique 11,787 [*R*(int) = 0.0400]; completeness to *θ* = 24.999 ° of 99.5%; semi-empirical absorption correction from equivalents; max. and min. transmission 0.9561 and 0.6884; data 11,787; restraints 678, parameters 1184; goodness-of-fit on *F*^2^ = 1.032, final indices [*I* > 2*σ* (*I*)] *R*_1_ = 0.0494, w*R*_2_ = 0.1274; *R* indices (all data) *R*_1_ = 0.0807, w*R*_2_ = 0.1383; largest diff. peak and hole 0.508 and −0.300 e Å^−3^.

## Online content

Any methods, additional references, Nature Portfolio reporting summaries, source data, extended data, supplementary information, acknowledgements, peer review information; details of author contributions and competing interests; and statements of data and code availability are available at https://doi.org/10.1038/s41557-026-02193-8.

## Supplementary information

Supplementary Information Supplementary Tables 1–8 and Figs. 1–28.

Peer Review File

Supplementary Video 1 Spin-state switching from **LS-1** to **HS-1** upon light irradiation with a white light torch resulting in a colour change from dark red to yellow/orange. The initial ‘HS’ signature was obtained as in Fig. 5b.

Supplementary Data 1 DFT optimized geometries of the LS, ^2^MLCT, ^4^MC, ^4^MLCT, ^6^MLCT and HS states.

## Data Availability

All pertinent experimental procedures, materials, methods and characterization data, as well as *xyz* coordinates of calculated geometries and a movie, are provided within this Article and its Supplementary Information. Crystallographic data for [Mn(pbmi)_2_][PF_6_]_2_ without and with irradiation have been deposited at the Cambridge Crystallographic Data Centre under deposition numbers 2503500 for **LS-1** [LS-Mn(pbmi)_2_][PF_6_]_2_) and 2503501 for **HS-1** [HS-Mn(pbmi)_2_][PF_6_]_2_). Copies of data are available via the CCDC at www.ccdc.cam.ac.uk/structures/. All raw data from this Article are available via Zenodo at https://doi.org/10.5281/zenodo.20025668 (ref. ^89^).
