# Supplementary material for: Covalency control of photomagnetic relaxation in a manganese(II) photoswitch
Source: Nat Chem. 2026 Jun 29;18(10):1729–36. doi: 10.1038/s41557-026-02193-8 (PMC13623647; doi:10.1038/s41557-026-02193-8)
Supplement: Supplementary file 1 [file 41557_2026_2193_MOESM1_ESM.pdf]

nature chemistry

Supplementary information https://doi.org/10.1038/s41557-026-02193-8

Covalency control of photomagnetic relaxation in a manganese(II) photoswitch

In the format provided by the authors and unedited

Table of Contents

Supplementary Table 1. Bond lengths of **LS-1** and **HS-1** from SCXRD 2
Supplementary Table 2. Spin density plots 3
Supplementary Table 3. DFT-calculated alpha molecular orbitals of **LS-1**. 4
Supplementary Table 4. DFT-calculated alpha molecular orbitals of **HS-1**. 7
Supplementary Figure 1. ESI^+ mass spectrum of **1** 9
Supplementary Figure 2. Temperature dependence of the magnetic susceptibility of **1** 10
Supplementary Figure 3. ^{1}H NMR spectrum of [H_2pbmi)][PF_6]$_2$ 11
Supplementary Figure 4. ^{19}F NMR spectrum of [H_2pbmi)][PF_6]$_2$. 12
Supplementary Figure 5. ^{1}H NMR spectrum of [Mn(pbmi)$_2$][PF_6] 23
Supplementary Figure 6. ^{19}F NMR spectrum of [Mn(pbmi)$_2$][PF_6] 14
Supplementary Figure 7. ^{1}H NMR spectrum of **1** 15
Supplementary Figure 8. ^{1}H, ^{1}H COSY spectrum of **1** 16
Supplementary Figure 9. ^{13}C{^{1}H} NMR spectrum of **1** 17
Supplementary Figure 10. ^{1}H, ^{13}C HSQC NMR spectrum of **1** 18
Supplementary Figure 11. Infrared (top) and Raman (bottom) spectra of **1** 19
Supplementary Table 5. Comparison of experimentally determined and DFT-calculated infrared transitions of **LS-1** 20
Supplementary Table 6. Comparison of experimentally determined and DFT-calculated Raman transitions of **LS-1**. 21
Supplementary Table 7. Charge transfer number analysis, TDDFT and difference electron densities of **LS-1** 22
Supplementary Figure 12. Thermal heating of **1** 27
Supplementary Table 8. Charge transfer number analysis, TDDFT and difference electron densities of **HS-1** 28
Supplementary Figure 13. Arrhenius plot. 31
Supplementary Figure 14. Absorption spectra LIESST experiment 32
Supplementary Figure 15. Emission spectra of the light sources 33
Supplementary Figure 16. Absorption spectra HS to LS conversion. 34
Supplementary Figure 17. fs-TA spectra and decay traces of **1** with λ_{exc} = 430 nm 35
Supplementary Figure 18. Rebinned data and EADS spectra with λ_{exc} = 430 nm 36
Supplementary Figure 19. Rebinned data and EADS spectra with λ_{exc} = 590 nm 37
Supplementary Figure 20. Rebinned data and EADS spectra with λ_{exc} = 590 nm and T = 233-293 K 39
Supplementary Figure 21. Schematic energy diagram of **1** 40
Supplementary Figure 22. Absorption spectra of **HS-1** 41
Supplementary Figure 23. Maximum **HS-1** quantum yield 42
Supplementary Figure 24. Magnetic Data during irradiation of **LS-1** with λ_{exc} = 590 nm
Supplementary Figure 25. Magnetic susceptibility data for T_{LIESST} with 2.5 K min^{-1} 44
Supplementary Figure 26. HS → LS relaxation curves in the solid state at different T 45
Supplementary Figure 27. Stability measurements in the solid state. 46
Supplementary Figure 28. Photographs of a single crystal of **1** before and after SCXRD 47

Supplementary Table 1. Selected Mn–L bond lengths (Å) obtained from SCXRD for **LS-1** (without irradiation) and **HS-1** (both independent molecules Mn1 and Mn2 in the asymmetric unit, as well as additional bond lengths in square brackets to one ligand from Mn2 due to the ligand split positions), which was generated by irradiation of LS-1 with a λ_{exc} = 560 nm light source at 91 K during the experiment, as well as (TD)DFT-optimized geometries for LS-1 and HS-1, as well as the excited states ^{4}MC, ^{2}MLCT, ^{4}MLCT[a] and ^{6}MLCT. Molecular structures obtained by SCXRD given for **LS-1** and **HS-1** (after irradiation of **LS-1** with a 560 nm light source). Ellipsoids are shown at a 50% probability level. Hydrogens, counterions, the solvent molecule and the second molecule in the asymmetric unit are omitted for clarity.

	**Mn-N_{py1}**	**Mn-N_{py2}**	**Mn-C_{NHC1}**	**Mn-C_{NHC2}**	**Mn-C_{NHC3}**	**Mn-C_{NHC4}**
**LS-1 (XRD, Mn1)**	1.944(3)	1.955(3)	1.994(3)	1.993(3)	2.003(3)	1.991(3)
**LS-1 (XRD, Mn2)**	1.999(8) [1.901(13)]	1.961(3)	2.015(8) [1.998(10)]	1.978(3)	1.984(9) [1.951(12)]	2.012(3)
**weighted average**	1.955(2)		1.995(1)			
**HS-1 (XRD, Mn1)**	2.335(3)	2.329(3)	2.264(4)	2.272(4)	2.287(4)	2.264(4)
**HS-1 (XRD, Mn2)**	2.314(5) [2.41(2)]	2.331(3)	2.272(5) [2.38(2)]	2.284(4)	2.285(5) [2.29(2)]	2.296(4)
**weighted average**	2.332(2)		2.277(2)			
**LS-1 (DFT)**	1.983	1.983	1.996	1.996	1.998	1.997
**HS-1 (DFT)**	2.383	2.384	2.278	2.275	2.274	2.274
**^{4}MC (TDDFT)**	2.224	2.226	2.078	2.058	2.058	2.054
**^{2}MLCT (TDDFT)**	2.010	1.952	2.027	2.091	2.026	1.982
**^{4}MLCT (TDDFT)** [a]	2.008	1.957	2.087	2.024	2.024	1.997
**^{6}MLCT (TDDFT)**	2.314	2.073	2.173	2.076	2.076	2.173

[a] DFT calculations based on *def2-SVP* basis set.

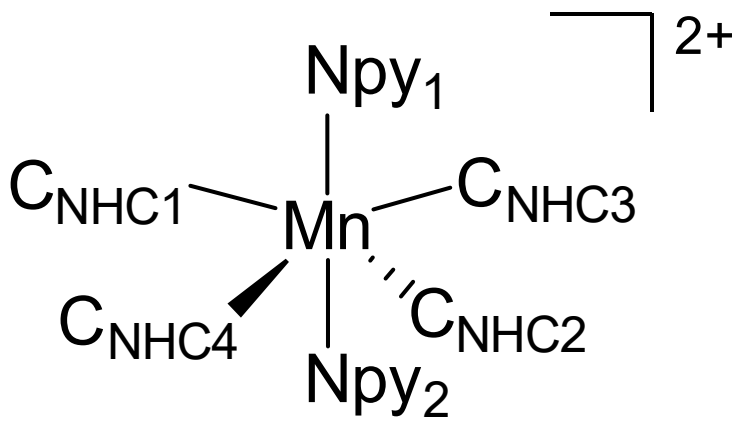

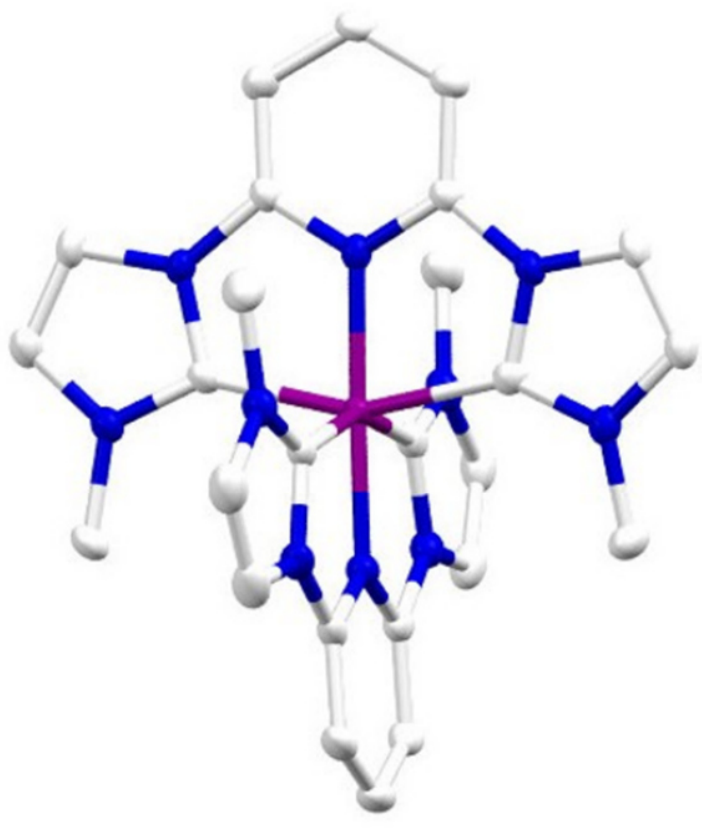

LS-1
CCDC no. 2503500

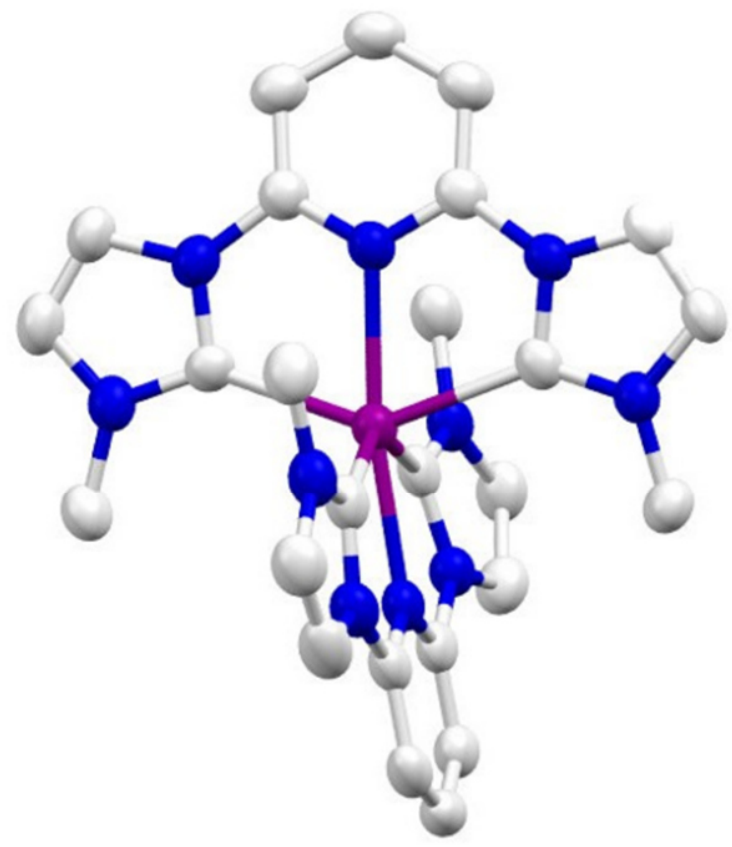

HS-1
CCDC no. 2503501

Supplementary Table 2. Spin density plots (α-spin density = blue; β-spin density = red) of geometry optimized states obtained from (TD)DFT calculations, displayed at an isosurface value of 0.01 a.u.. Hydrogens are omitted for clarity.

	**LS-1**	**^{4}MC**	**HS-1**
	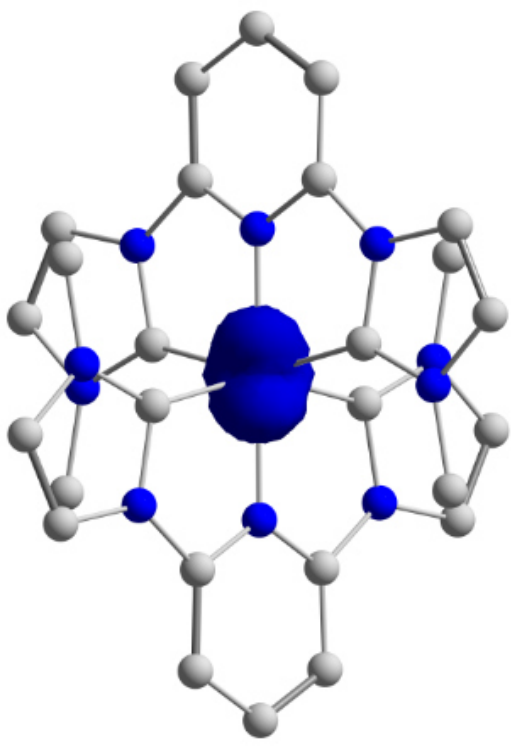	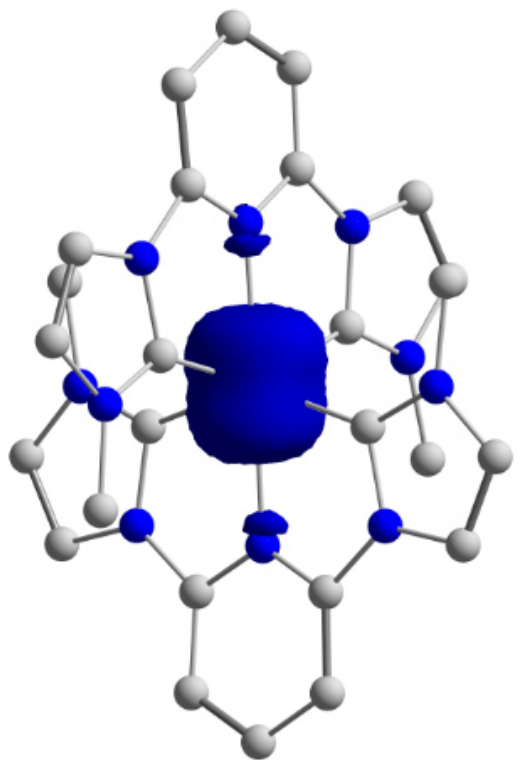	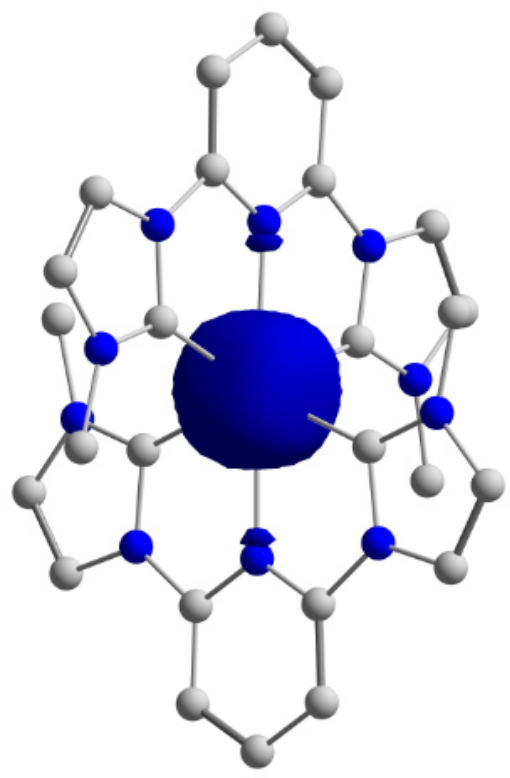
E / eV	−73935.646156	−73935.306788	−73935.532390
<S^2> (expected <S^2>)	0.786723 (0.750000)	3.791005 (3.750000)	8.757201 (8.750000)
	**^{2}MLCT**	**^{4}MLCT[a]**	**^{6}MLCT**
	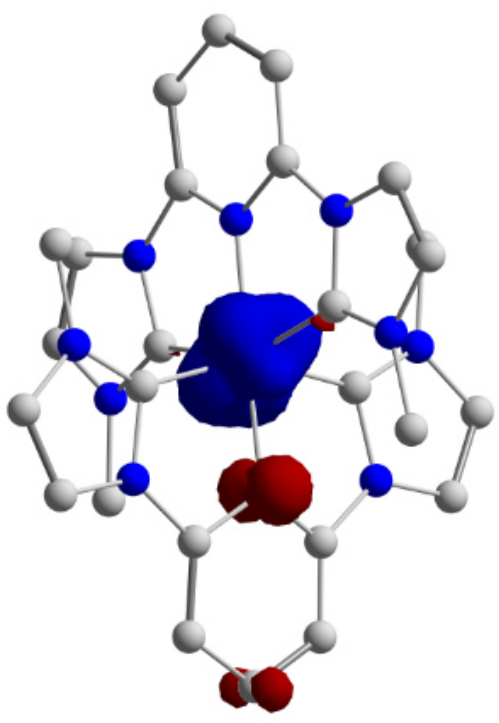	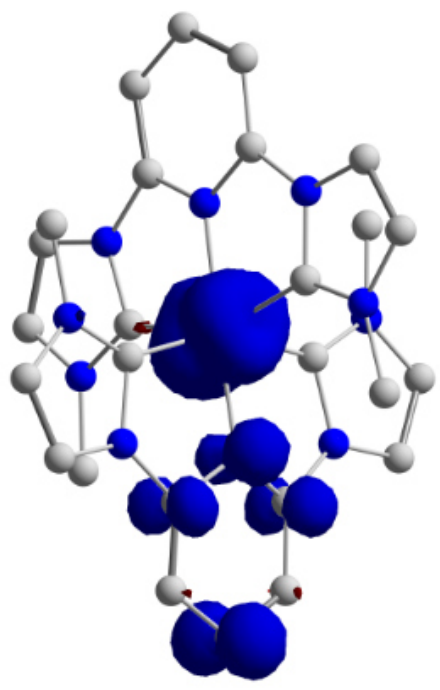	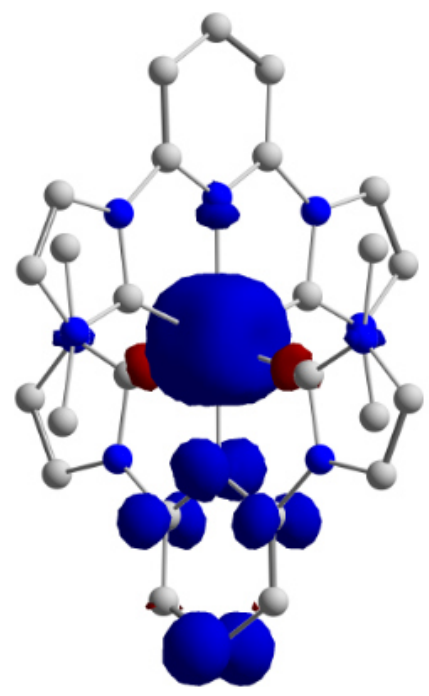
E / eV	−73933.866502	−73884.545940[a]	−73934.283844
<S^2> (expected <S^2>)	0.832228 (0.750000)	3.790978 (3.750000)[a]	8.757246 (8.750000)

[a] DFT calculations based on *def2-SVP* basis set.

Supplementary Table 3. Selected DFT-calculated alpha molecular orbitals of **LS-1** displayed at an isosurface value of 0.05 a.u., energies given in eV. Hydrogens are omitted for clarity.

LUMO+14 / 1.0367	LUMO+13 / 0.8688	LUMO+12 / 0.7957
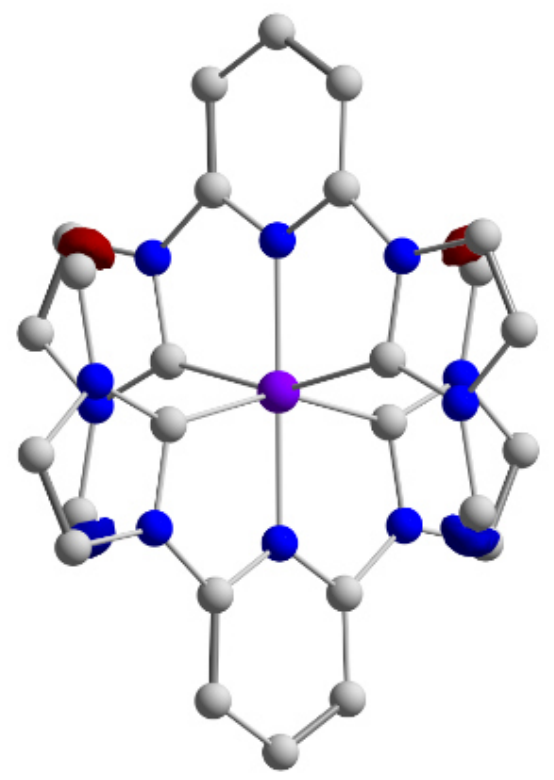	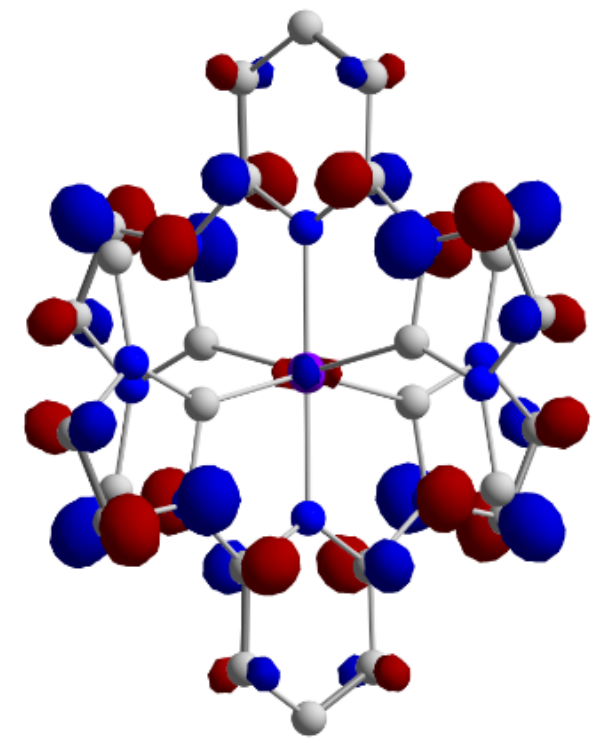	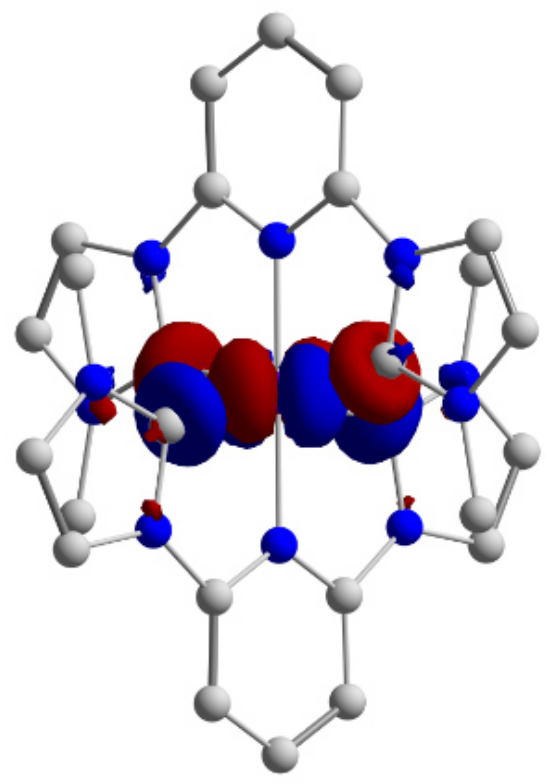
LUMO+11 / 0.5145	LUMO+10 / 0.4147	LUMO+9 / 0.2952
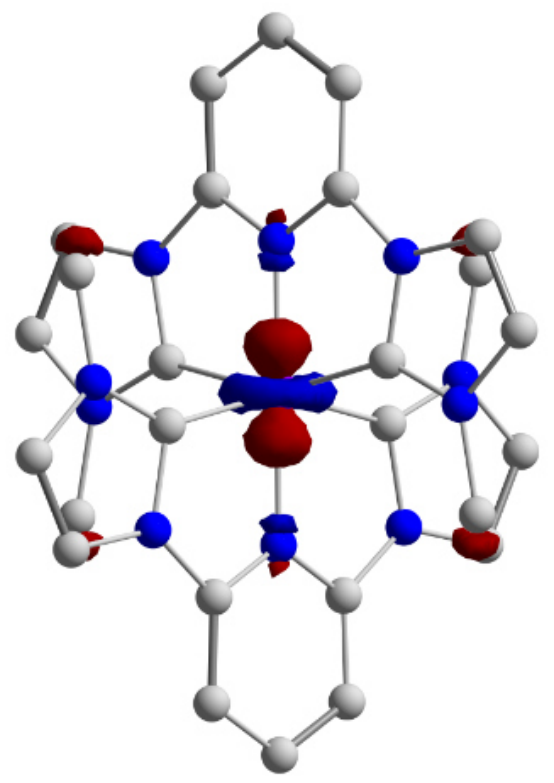	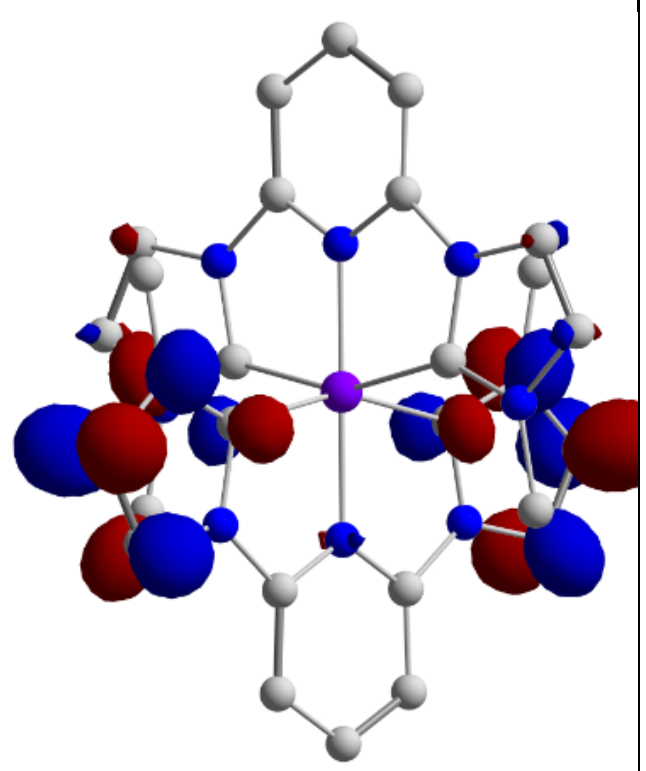	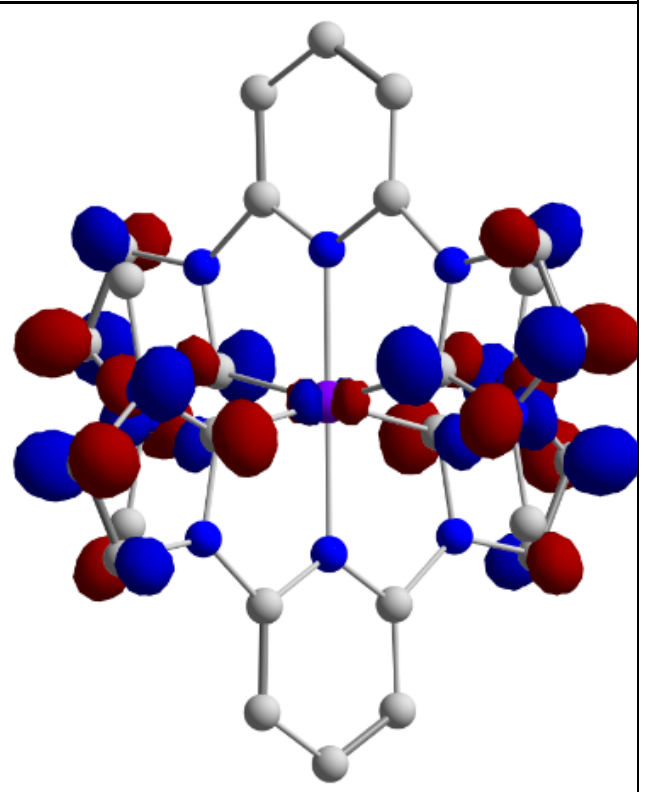
LUMO+8 / 0.2909	LUMO+7 / 0.2014	LUMO+6 / 0.0574
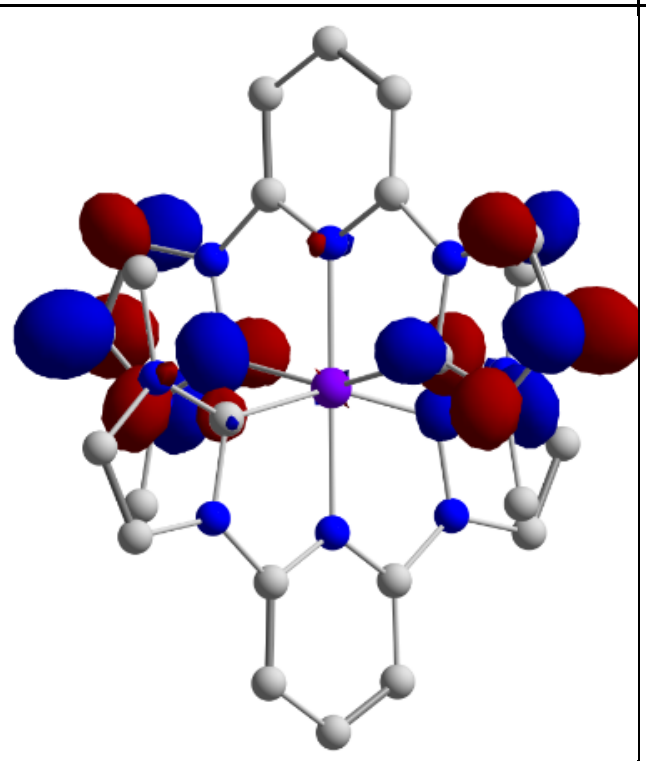	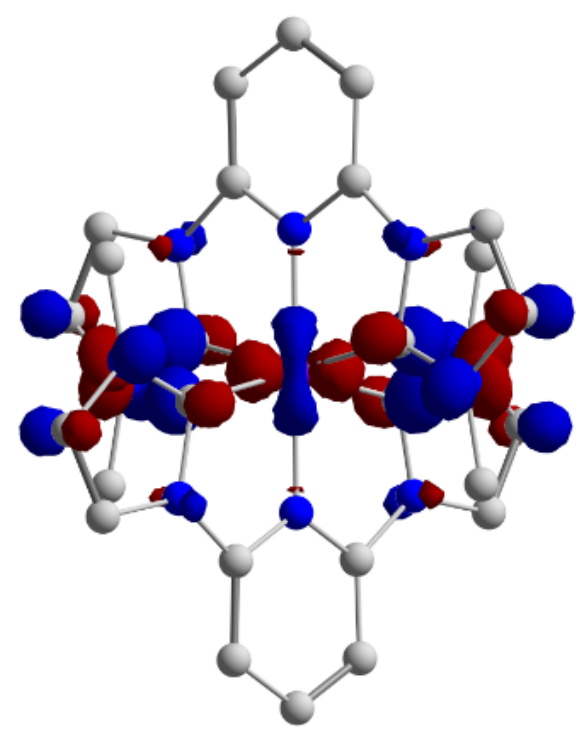	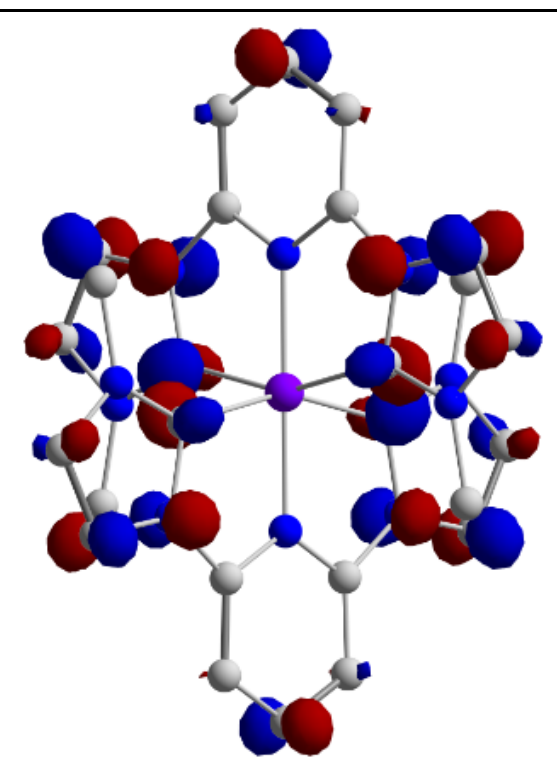

LUMO+5 / −0.0266	LUMO+4 / −0.1094	LUMO+3 / −1.4435
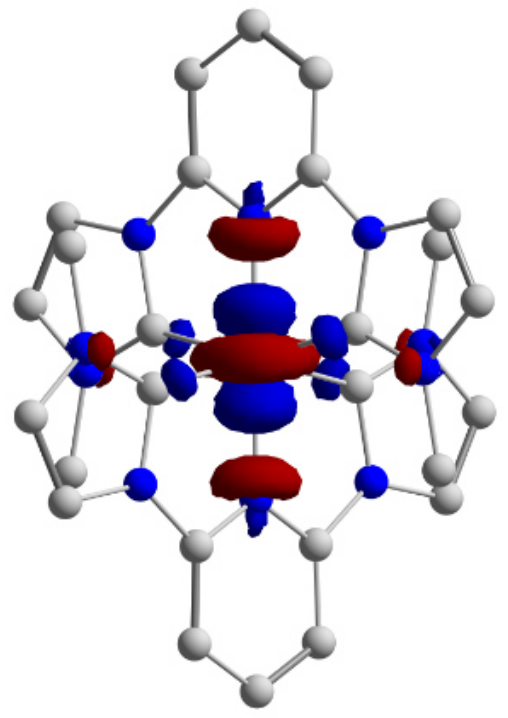	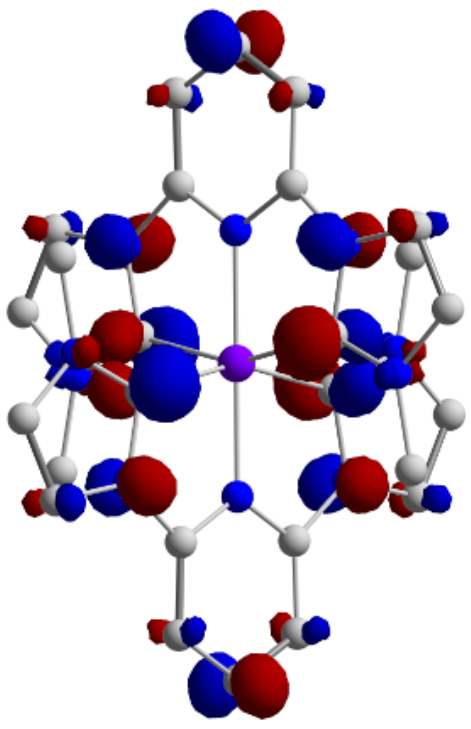	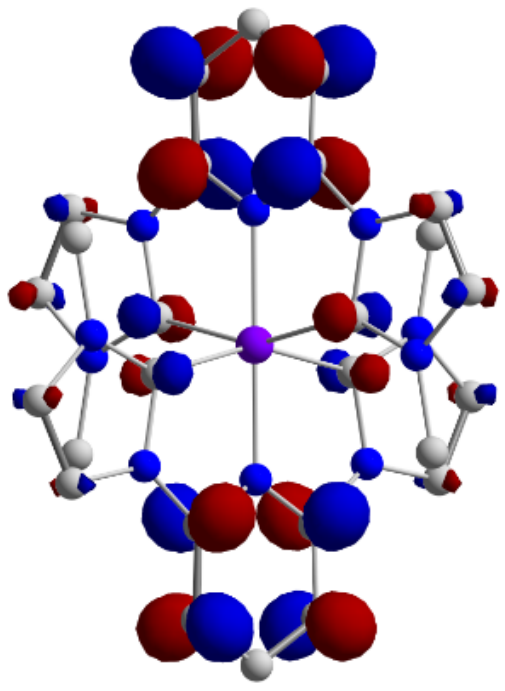
LUMO+2 / −1.4840	LUMO+1 / −1.7566	LUMO / −1.8862
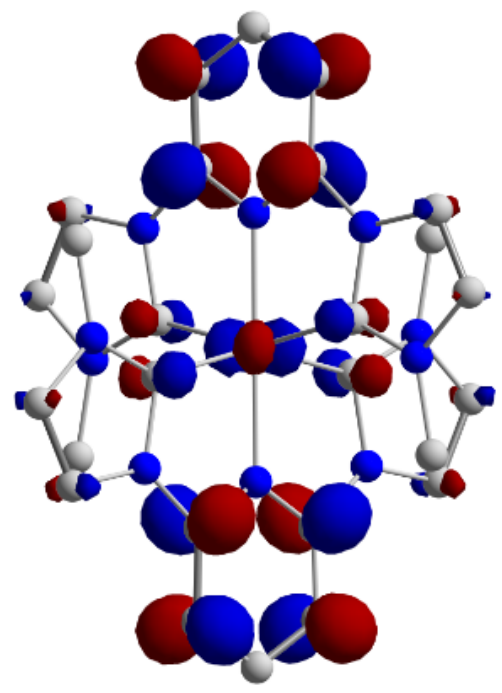	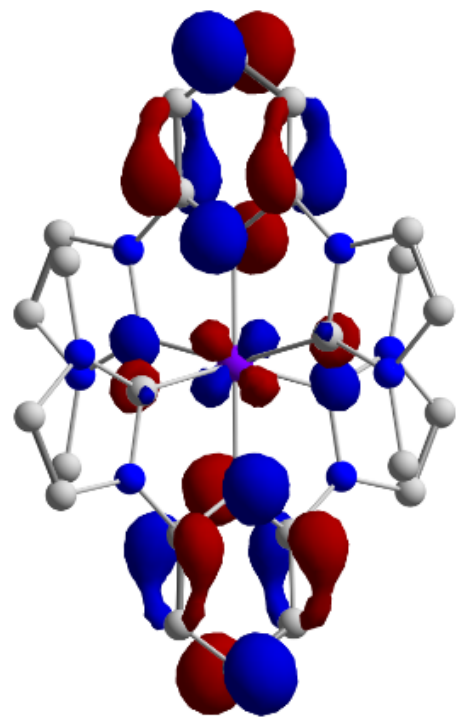	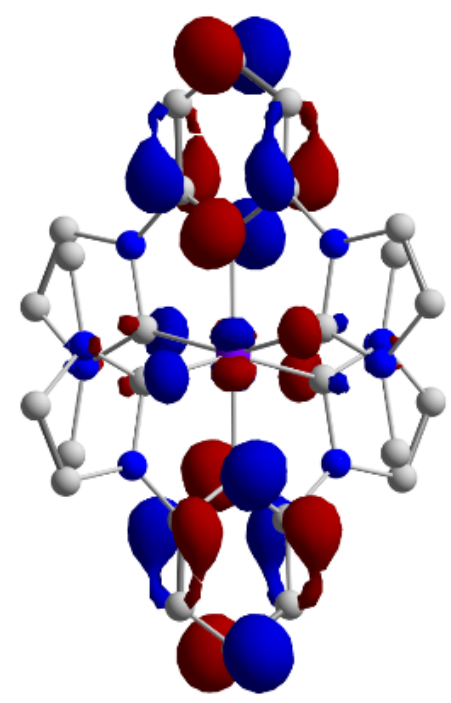
HOMO / −5.5270	HOMO−1 / −5.8350	HOMO−2 / −6.6333
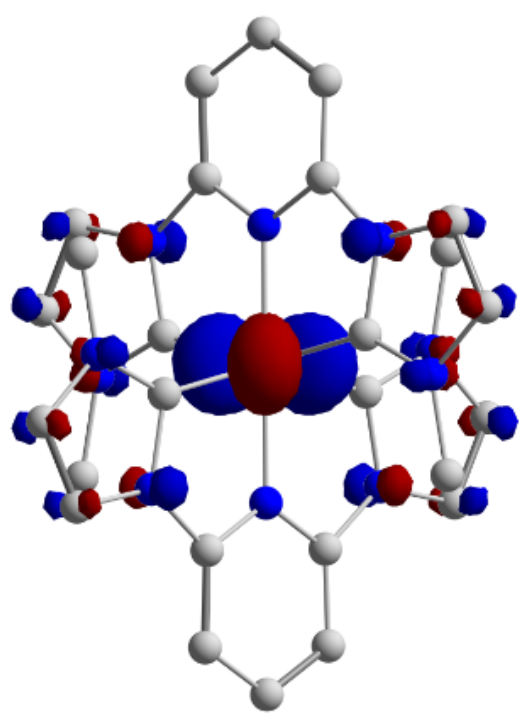	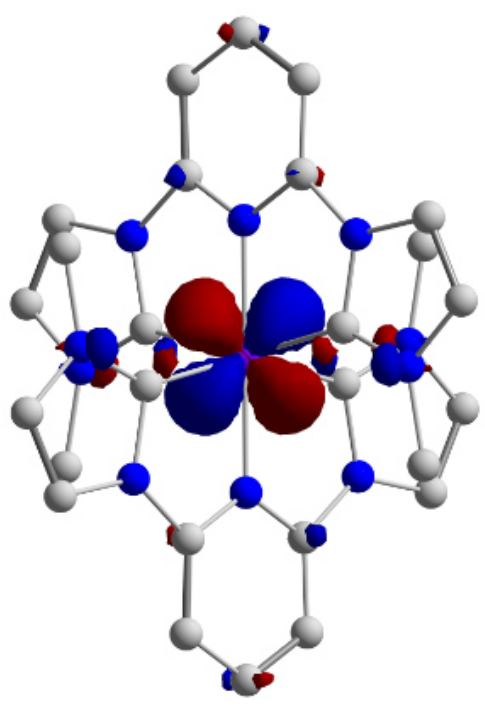	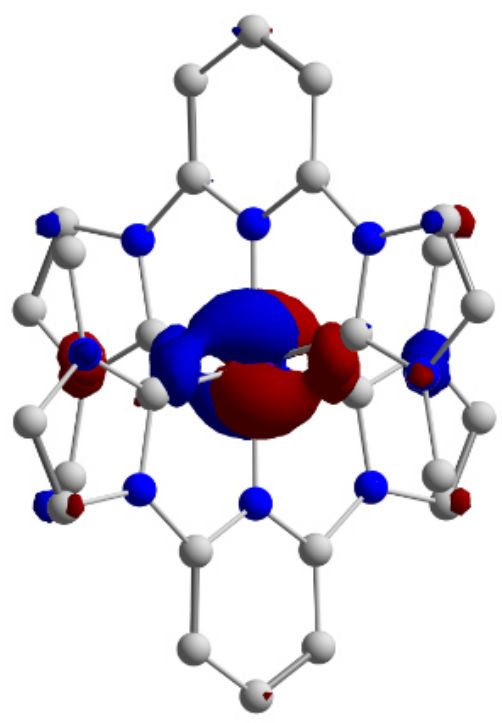
HOMO−3 / −6.7298	HOMO−4 / −6.9647	HOMO−5 / −7.1145
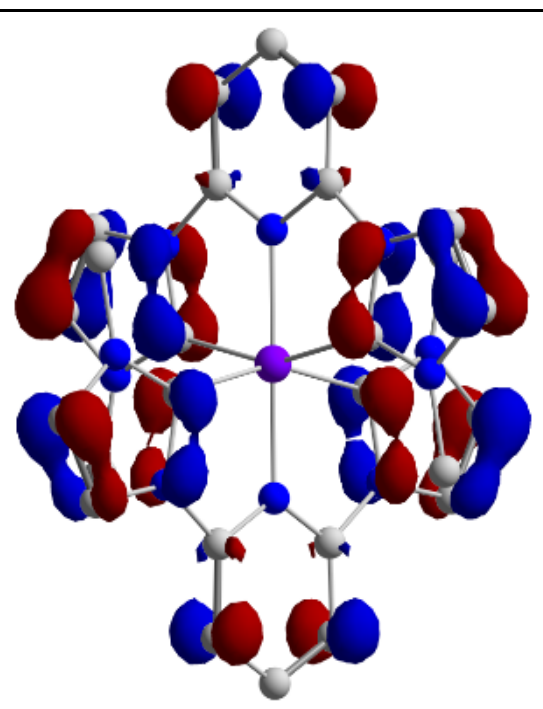	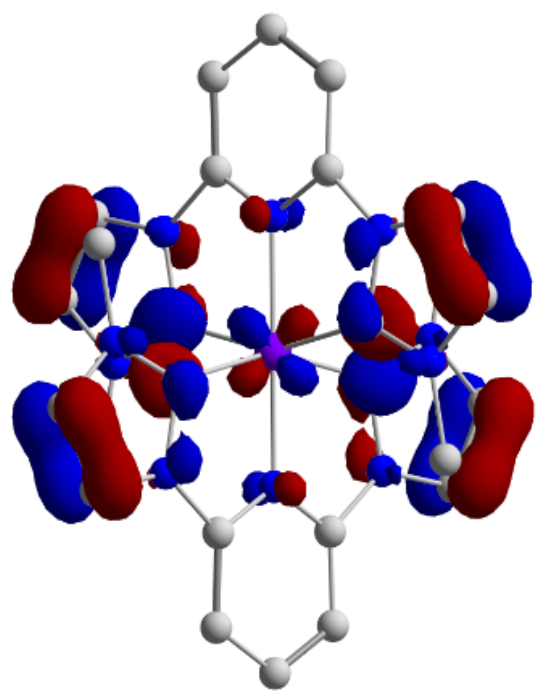	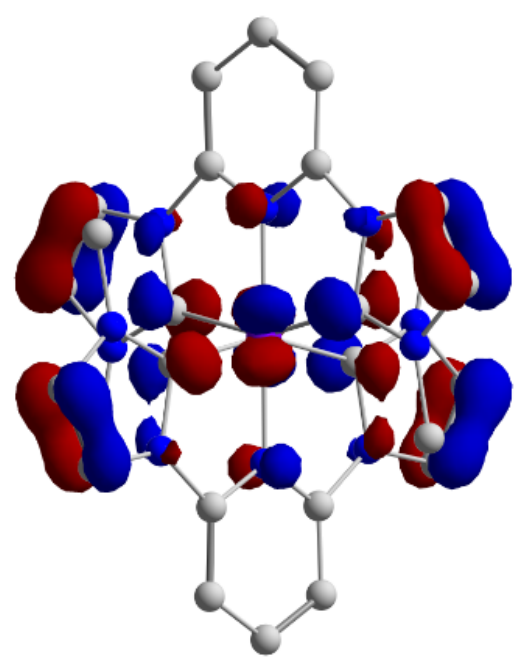

HOMO−6 / −7.1336	HOMO−7 / −7.3739	HOMO−8 / −8.0146
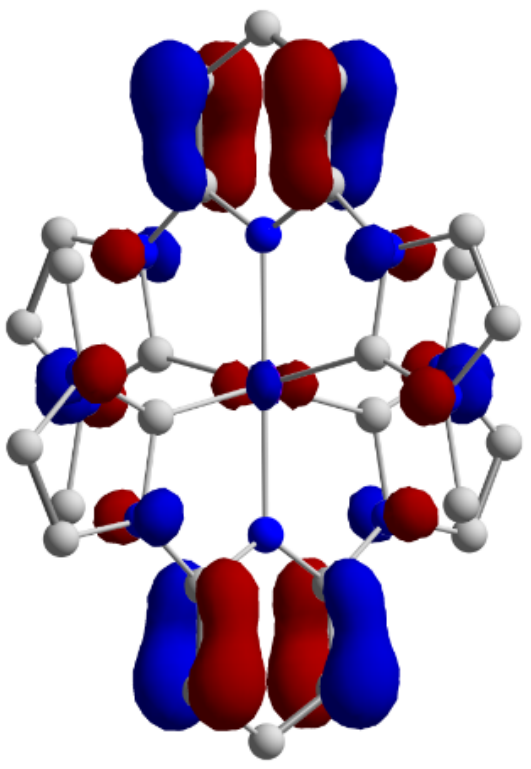	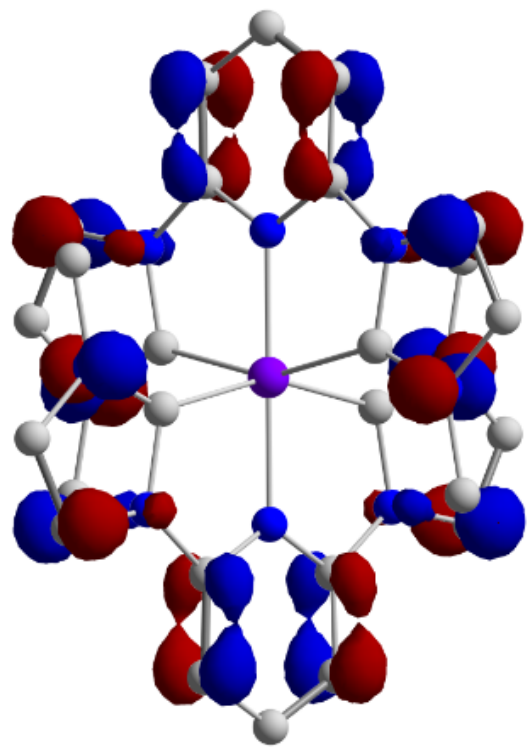	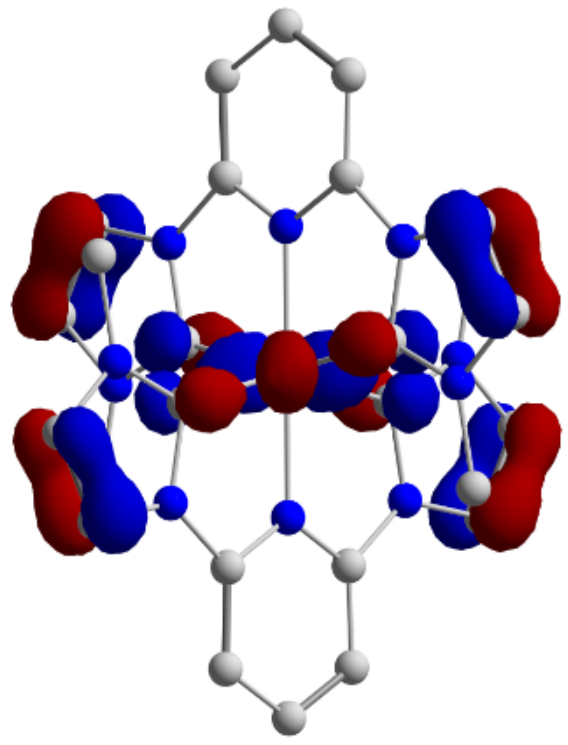

Supplementary Table 4. Selected DFT-calculated alpha molecular orbitals of **HS-1** displayed at an isosurface value of 0.05 a.u., energies given in eV. Hydrogens are omitted for clarity.

LUMO+2 / −1.5505	LUMO+1 / −1.8178	LUMO / −1.8201
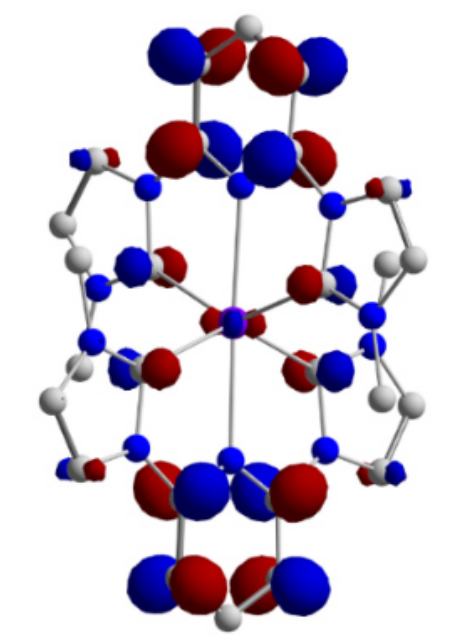	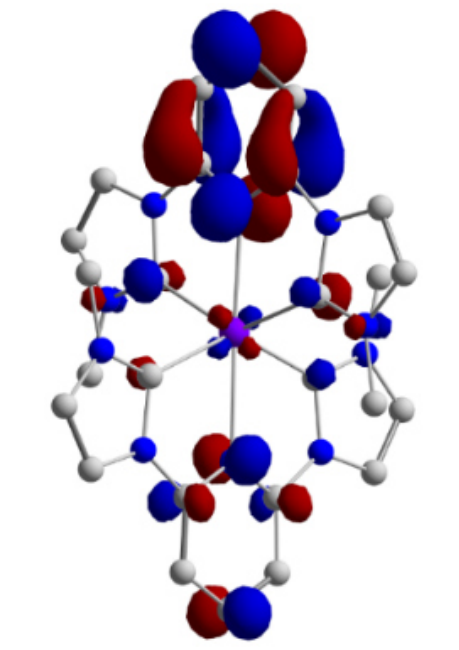	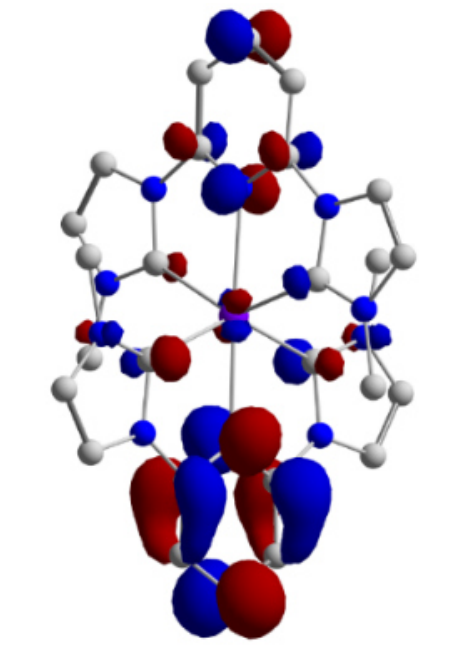
HOMO / −4.8434	HOMO−1 / −5.9035	HOMO−2 / −6.6981
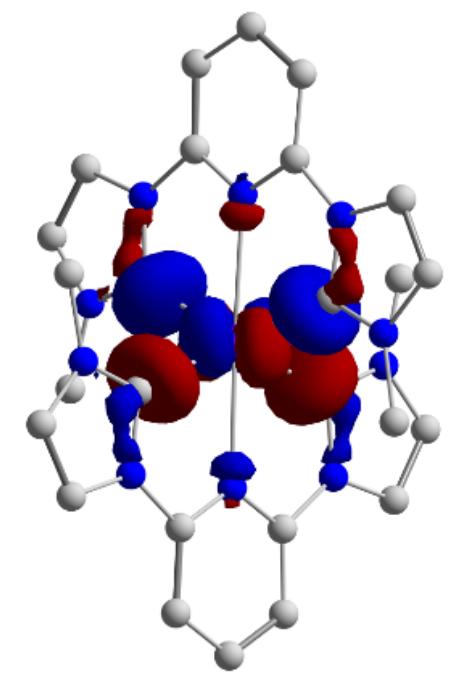	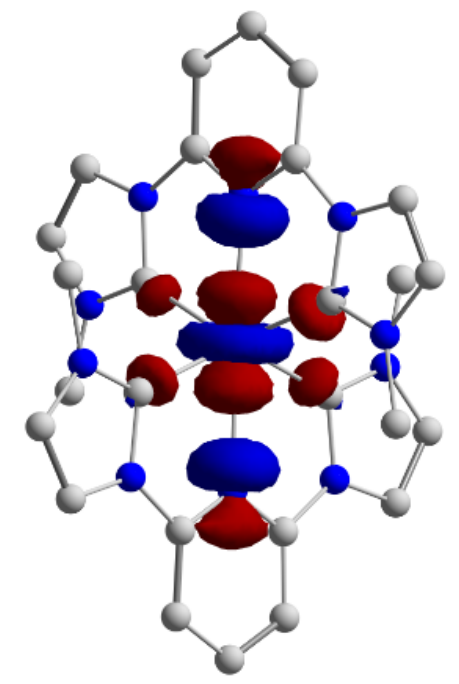	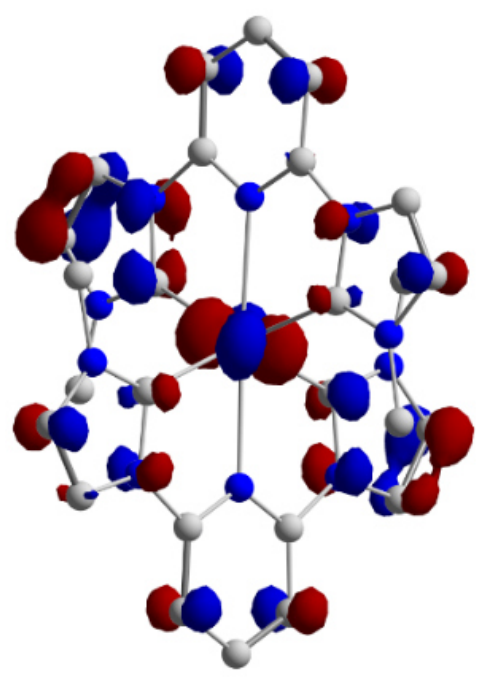
HOMO−3 / −6.7101	HOMO−4 / −6.7155	HOMO−5 / −6.8483
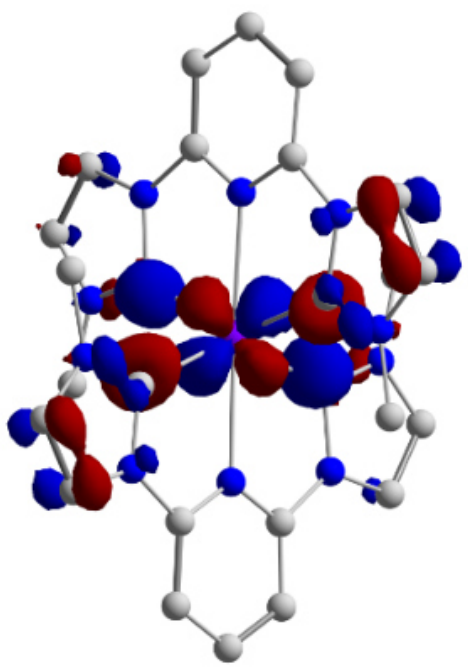	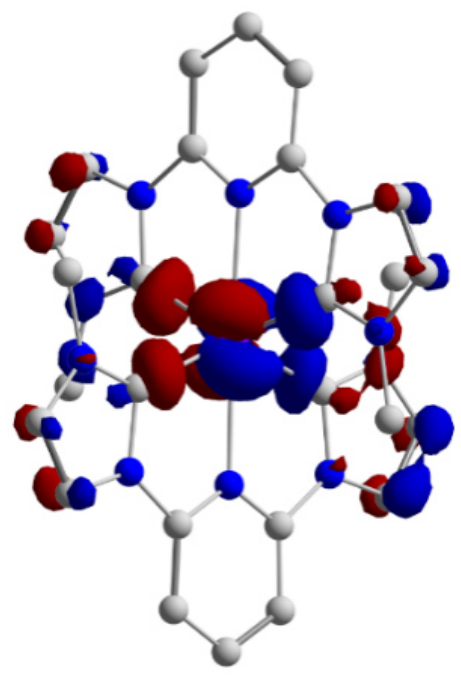	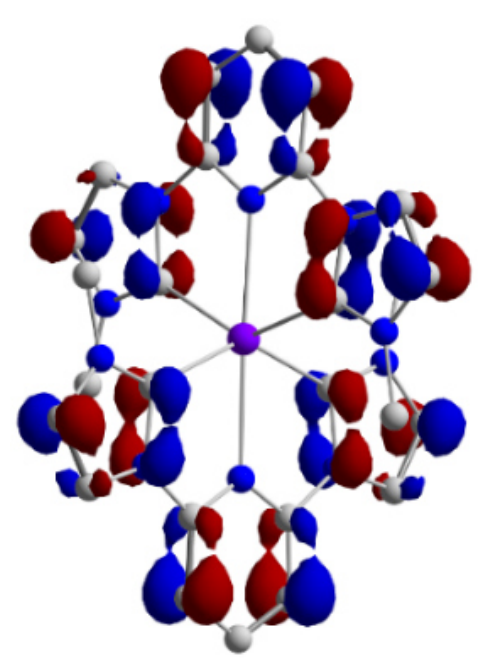
HOMO−6 / −7.2502	HOMO−7 / −7.2529	HOMO−8 / −7.2609
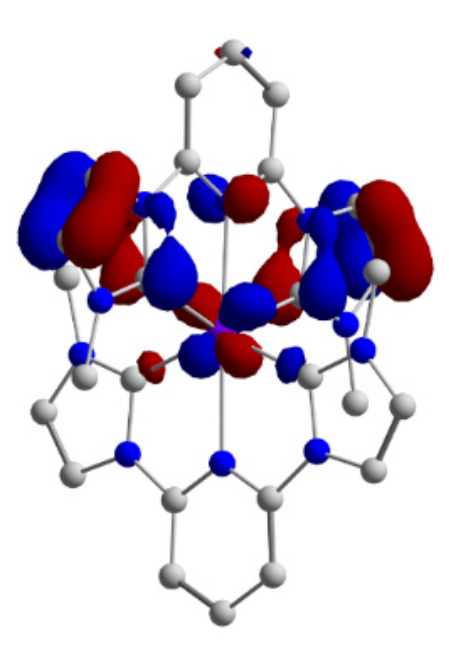	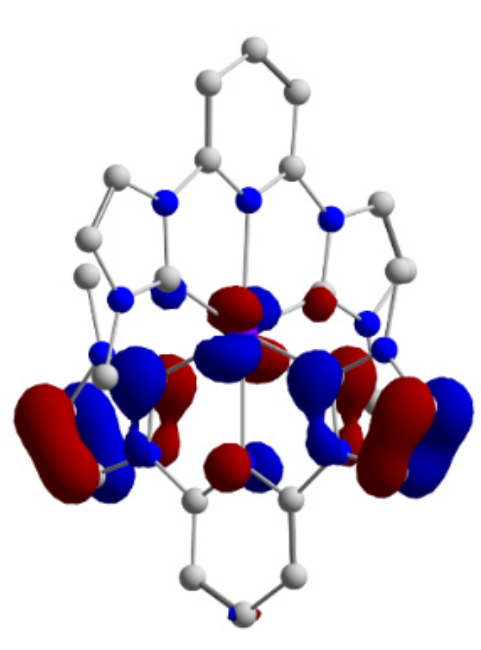	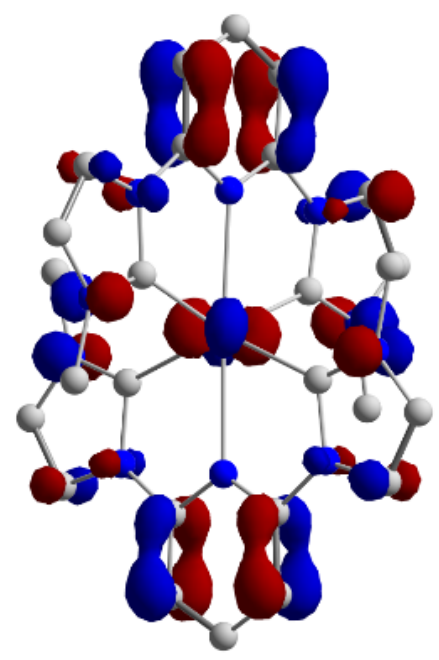

HOMO−9 / −7.3633	HOMO−10 / −8.0257	HOMO−11 / −8.3742
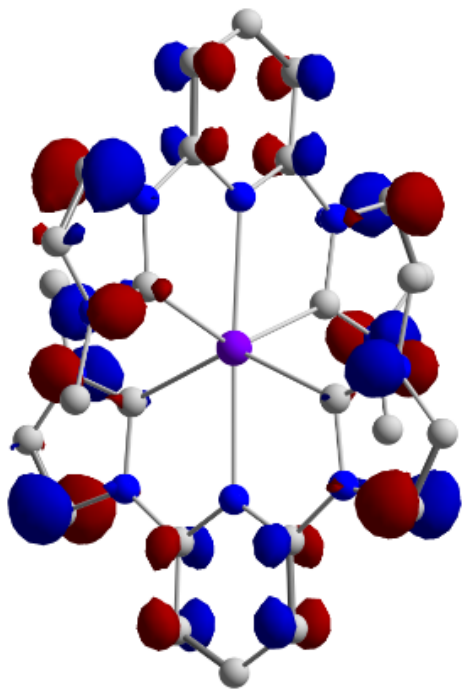	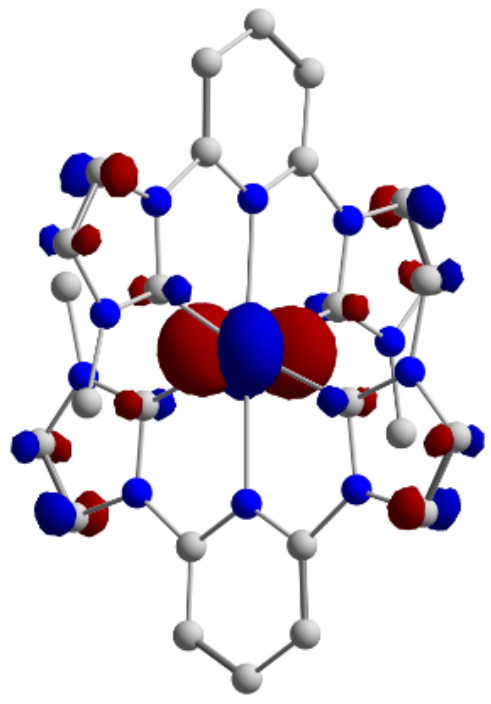	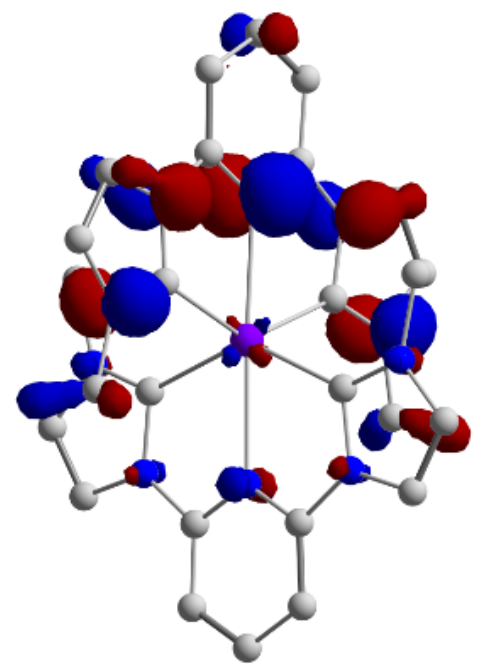
HOMO−12 / −8.3759	HOMO−13 / −8.7826	HOMO−14 / −8.7856
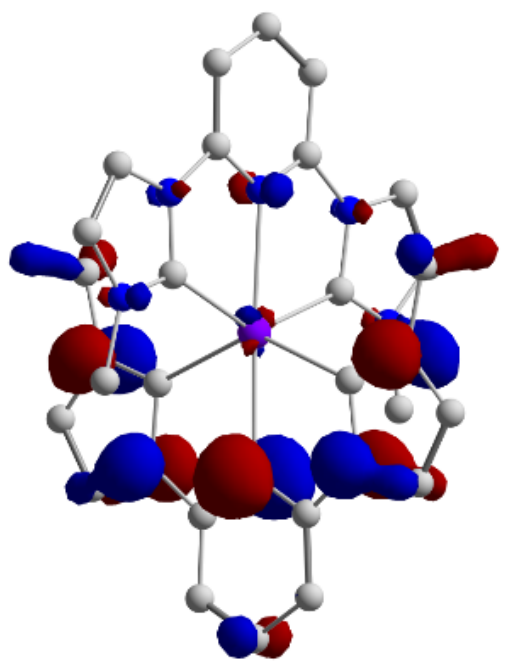	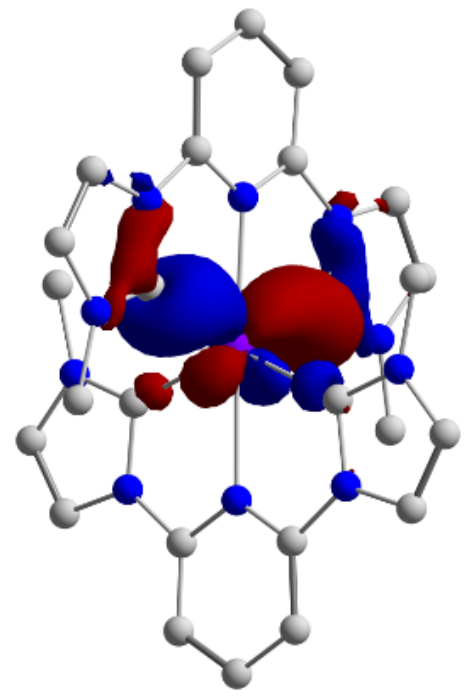	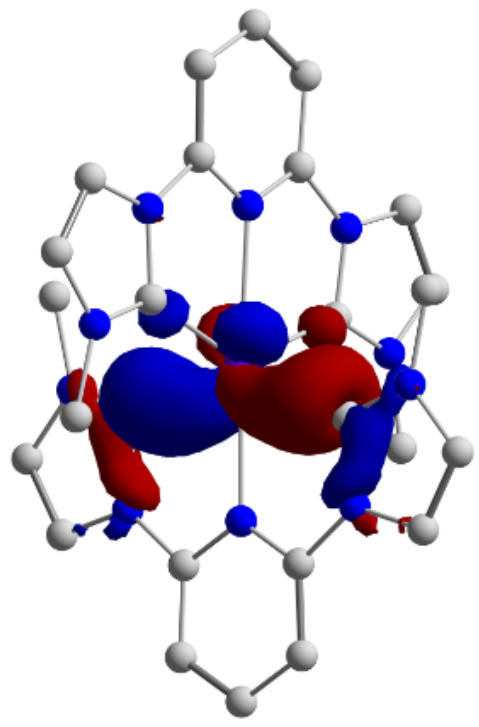

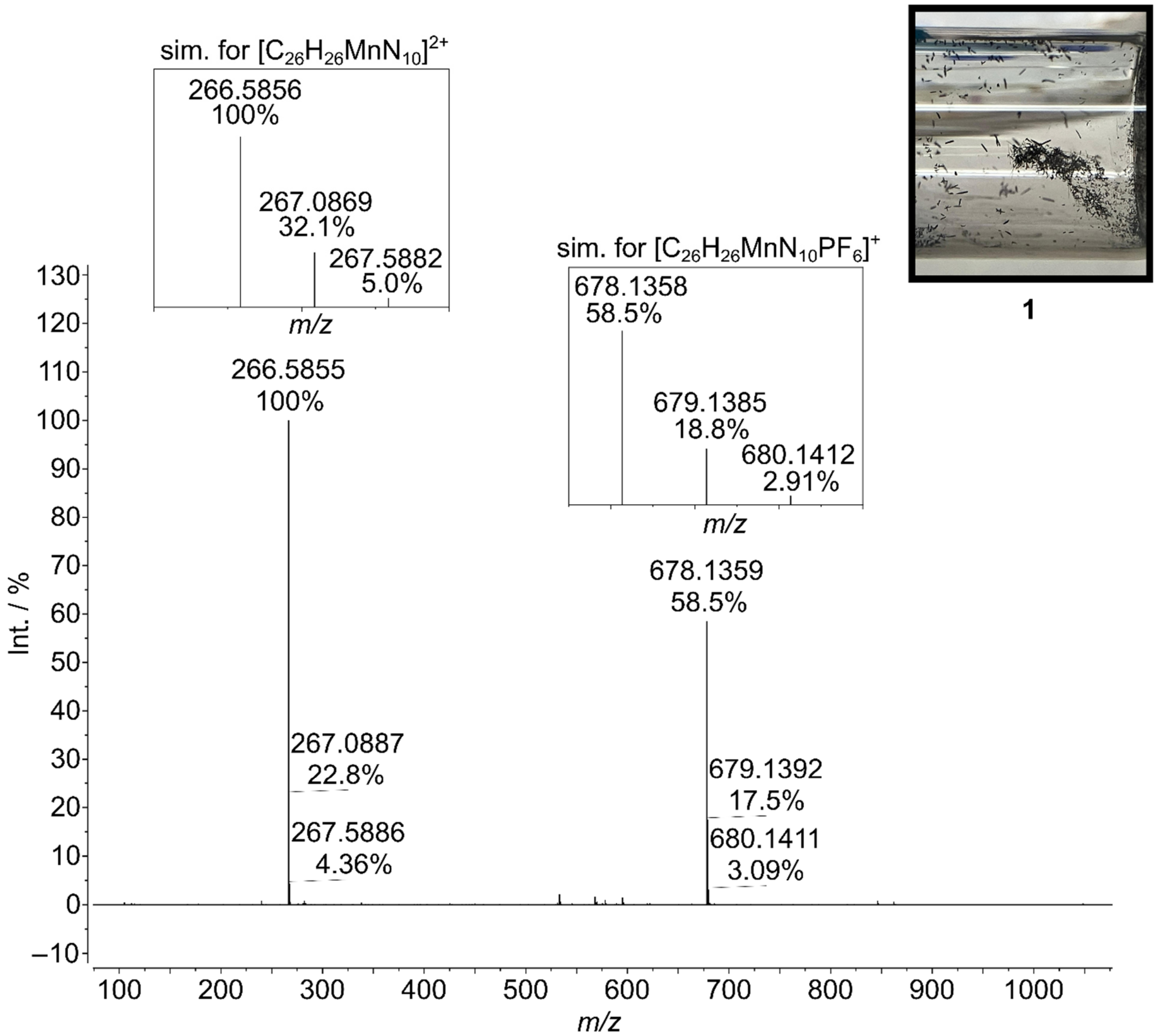

1

Supplementary Figure 1. ESI^{+} mass spectrum of 1 $[Mn(pbmi)_2][PF_6]_2$ in dry MeCN. The inset shows the calculated isotopic pattern for the peaks at *m*/*z* 266.5856 for $[Mn(pbmi)_2]^{2+}$ ($C_{26}H_{26}MnN_6$) and *m*/*z* 678.1358 for $\{[Mn(pbmi)_2][PF_6]\}^{+}$ ($C_{26}H_{26}MnN_{10}PF_6$). Photograph of crystals of **1** at 293 K.

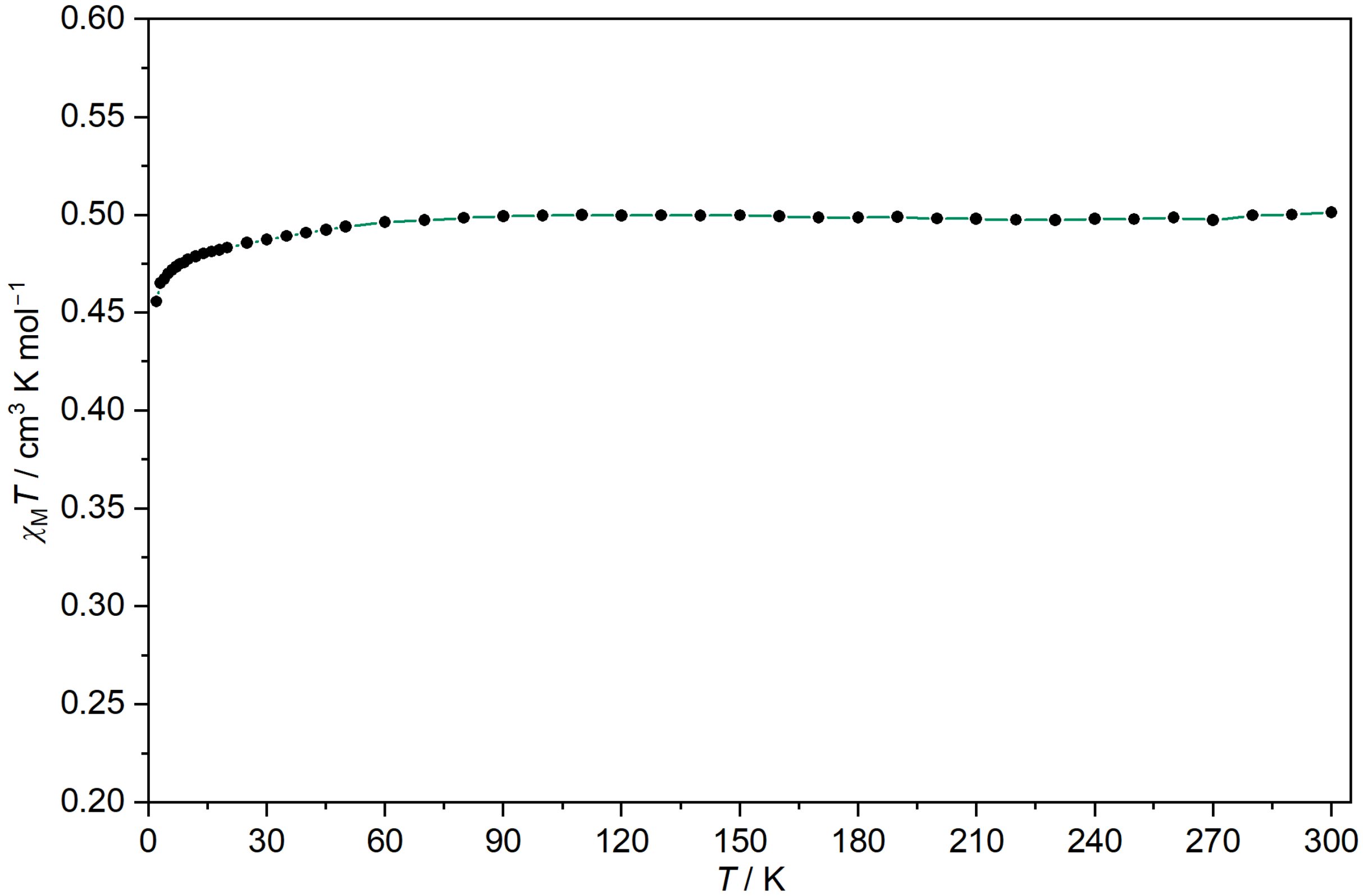

Supplementary Figure 2. Temperature dependence of the magnetic susceptibility of solid **1** plotted as $\chi_M T$ vs T.

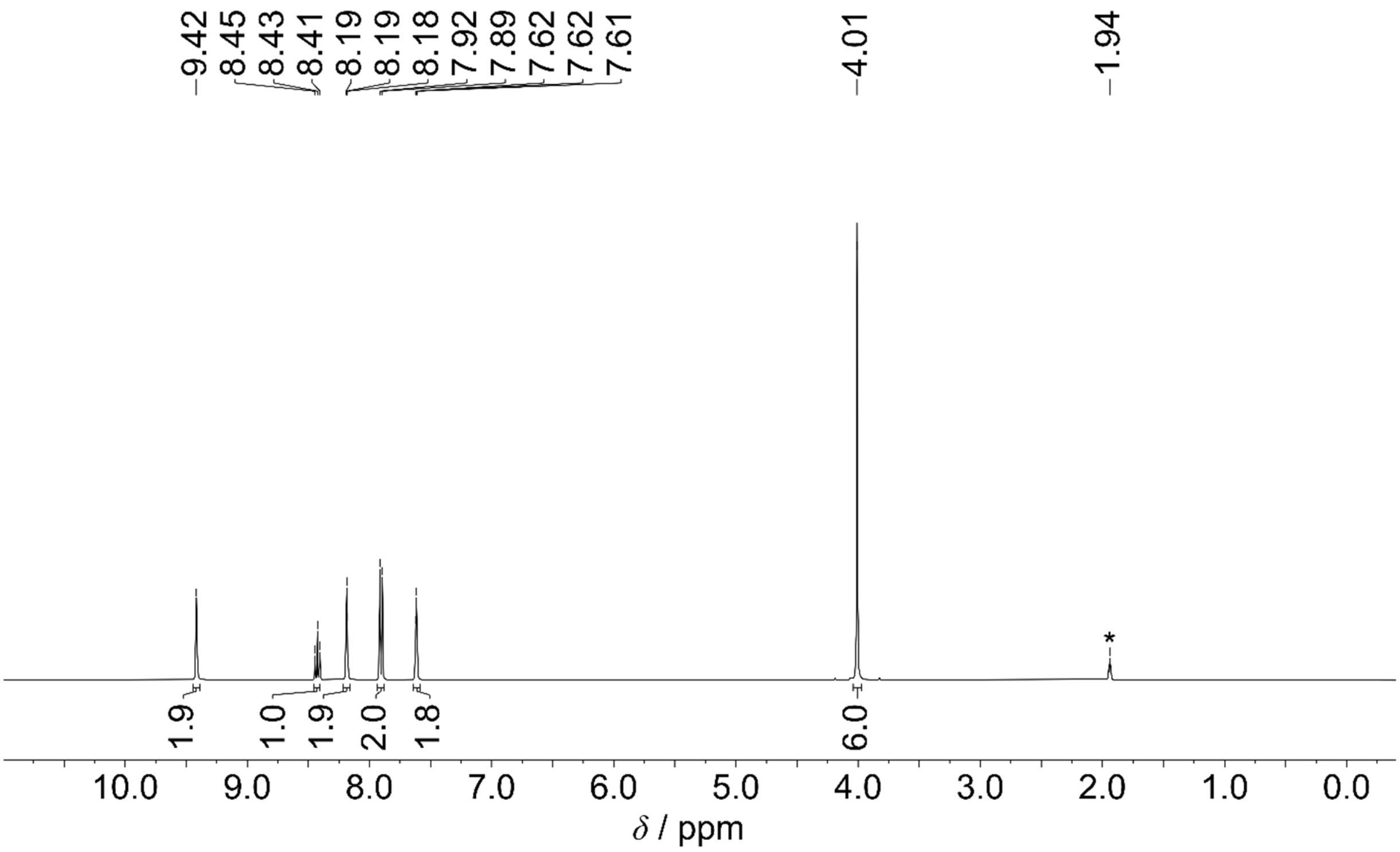

Supplementary Figure 3. ^{1}H NMR spectrum of [H_2pbmi)][PF_6]$_2$ in CD_3CN at 293 K. The asterisk denotes residual solvent resonance.

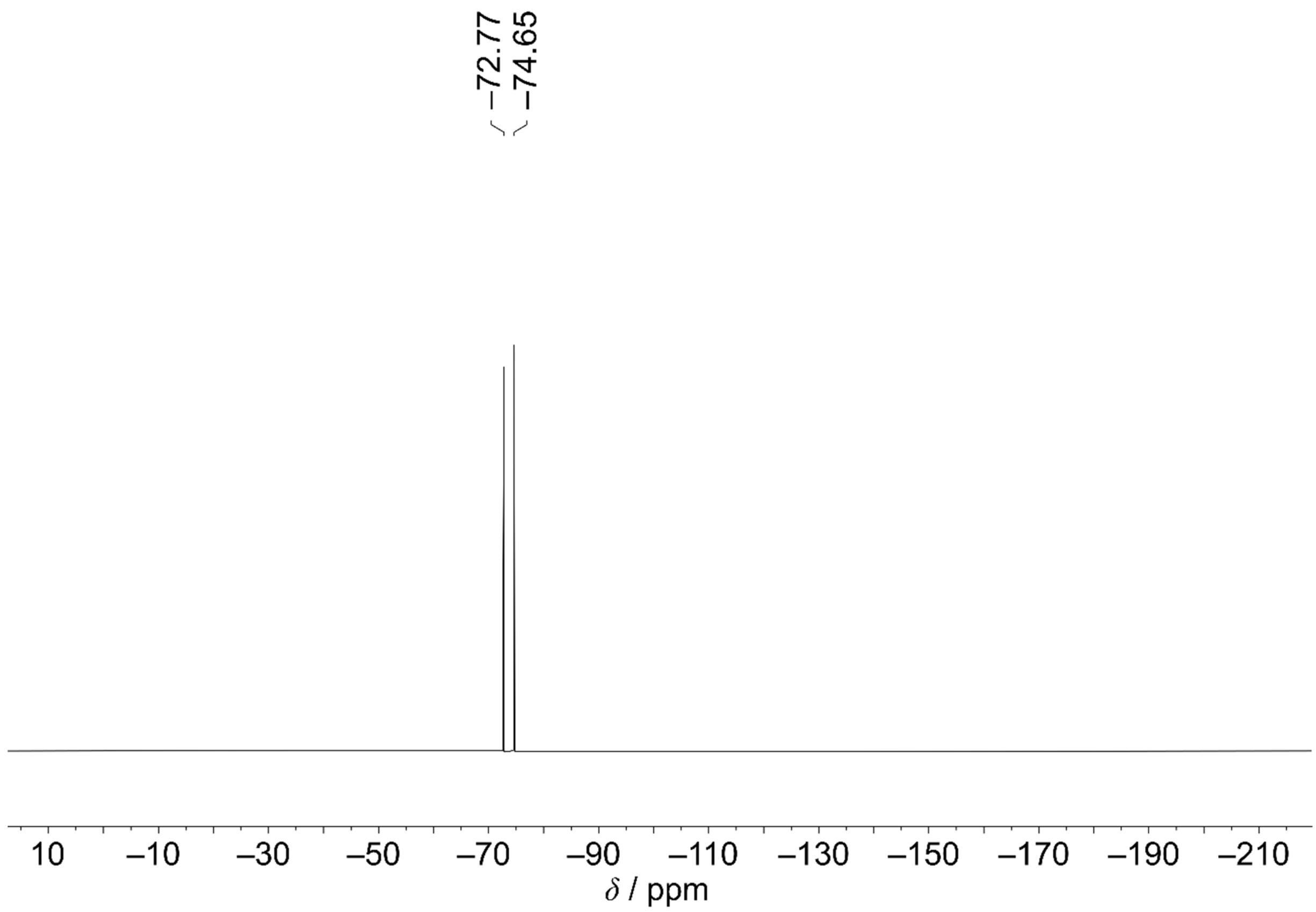

Supplementary Figure 4. ^{19}F NMR spectrum of [H_2pbmi)][PF_6]$_2$ in CD_3CN at 293 K.

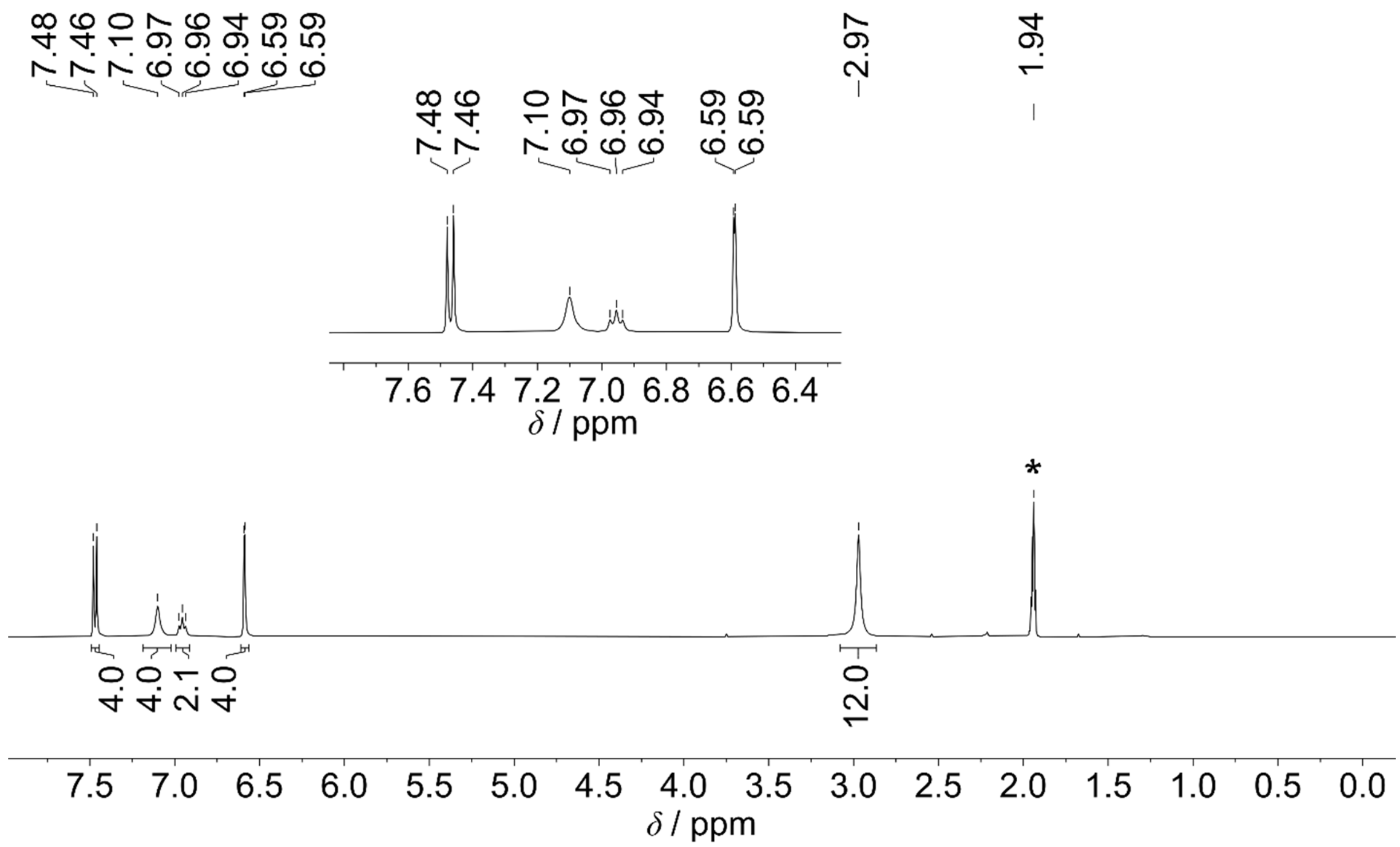

Supplementary Figure 5. ^{1}H NMR spectrum of $[Mn(pbmi)_2][PF_6]$ in CD_3CN at 293 K. The asterisk denotes residual solvent resonance.

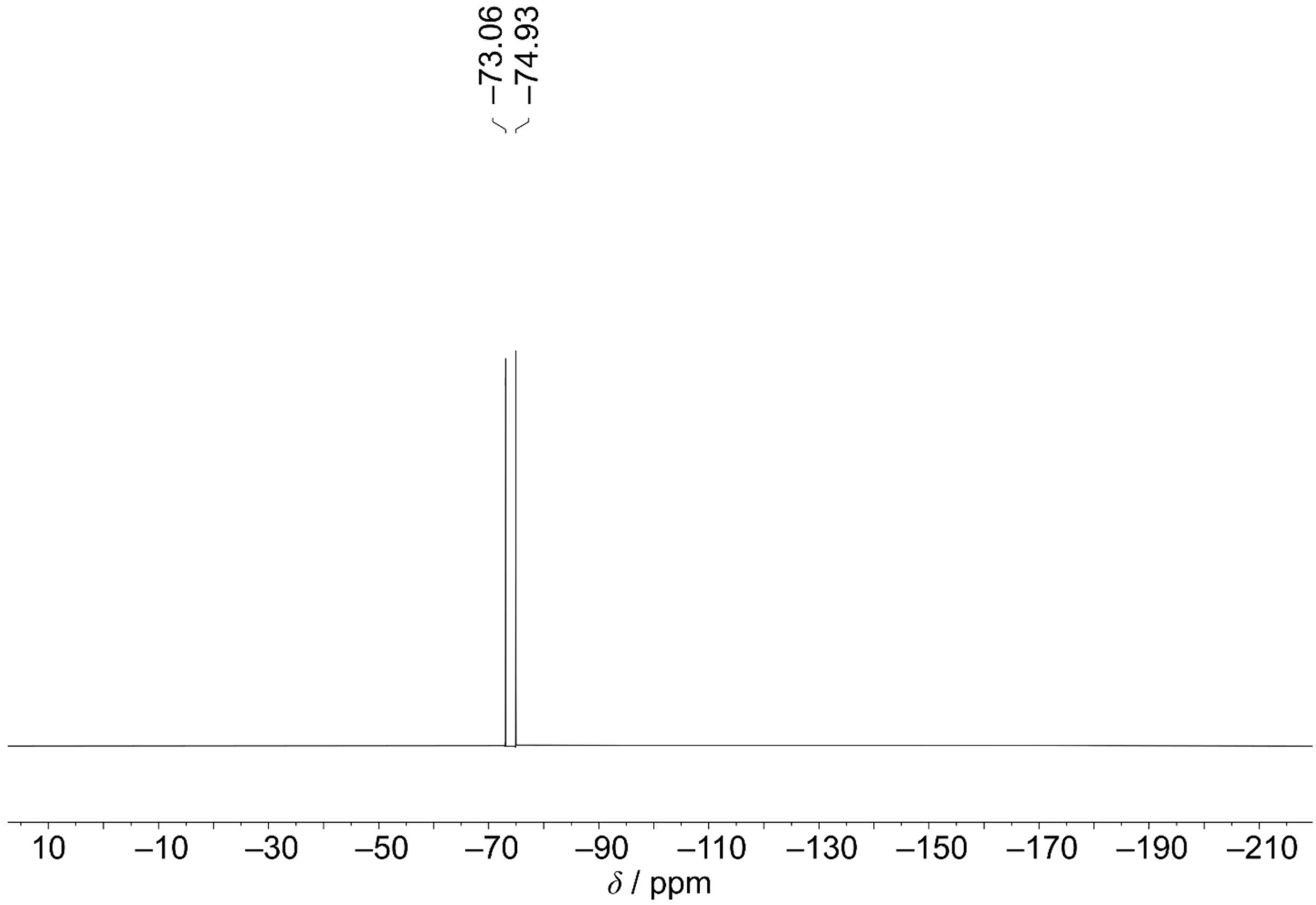

Supplementary Figure 6. ^{19}F NMR spectrum of [Mn(pbmi)$_2$][PF$_6$] in CD_3CN at 293 K.

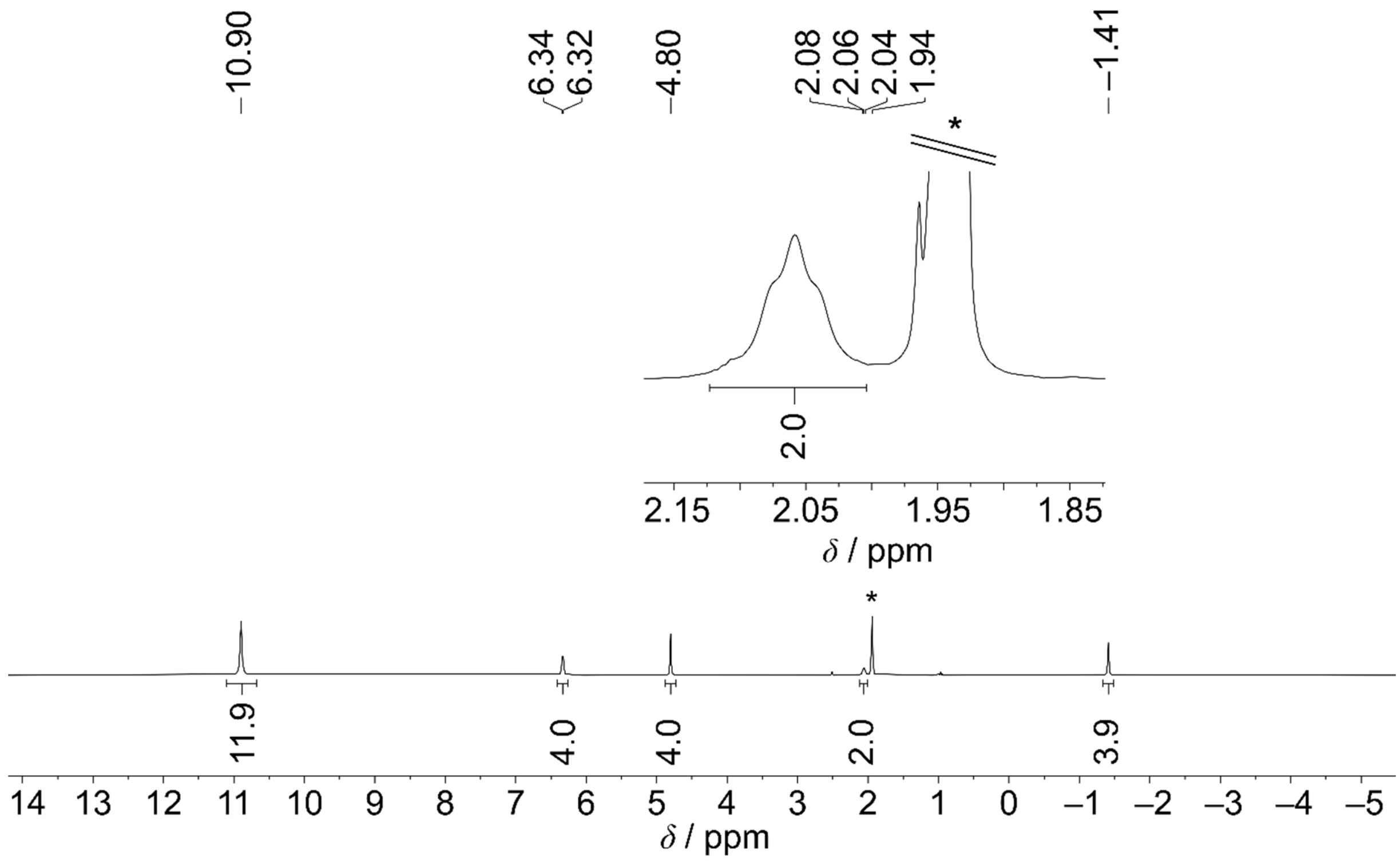

Supplementary Figure 7. ^{1}H NMR spectrum of **1** $[Mn(pbmi)_2][PF_6]_2$ in CD_3CN at 298 K. The asterisk denotes residual solvent resonance.

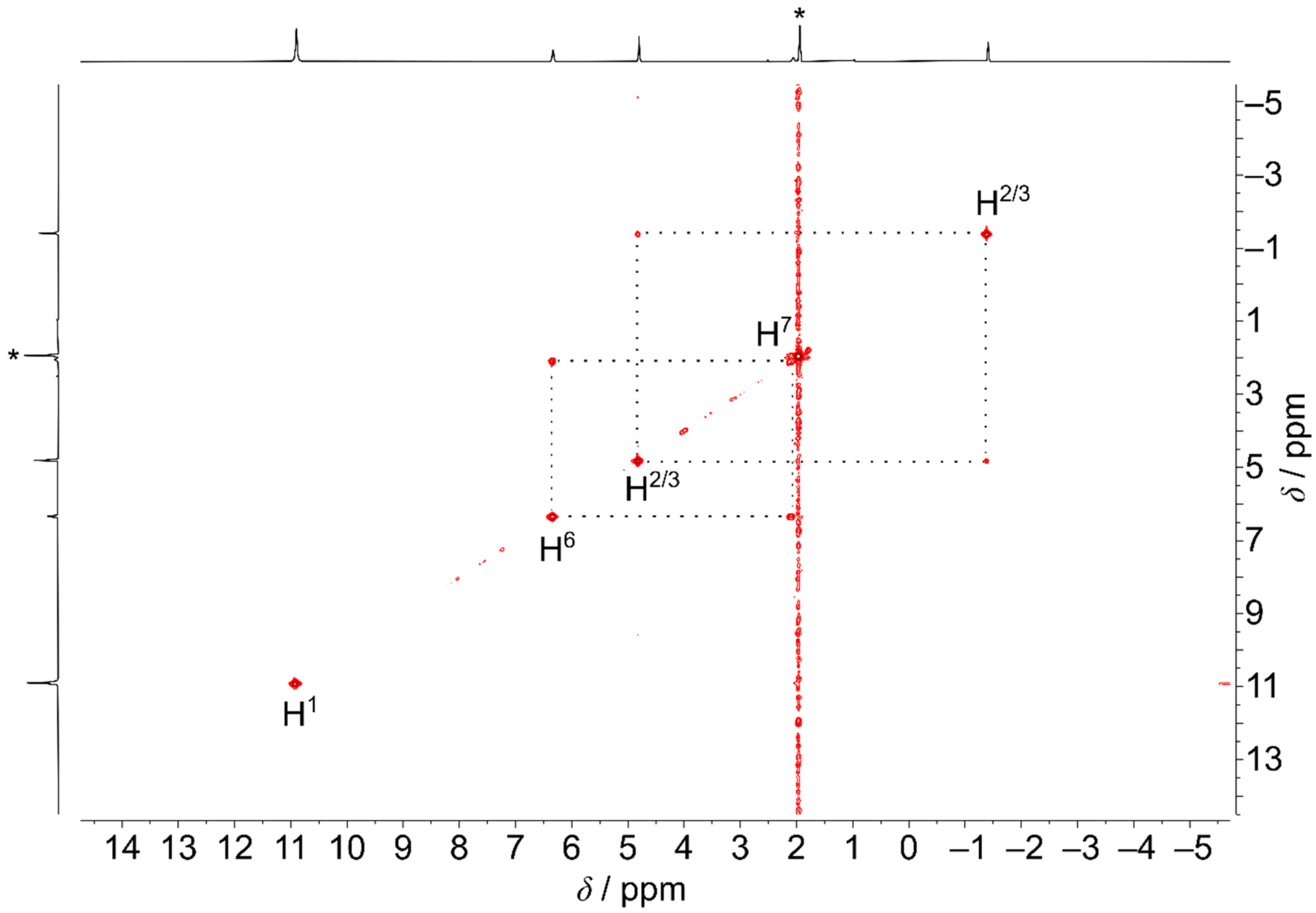

Supplementary Figure 8. ^{1}H, ^{1}H COSY spectrum of **1** $[Mn(pbmi)_2][PF_6]_2$ in CD_3CN at 298 K. The asterisks denote residual solvent resonances.

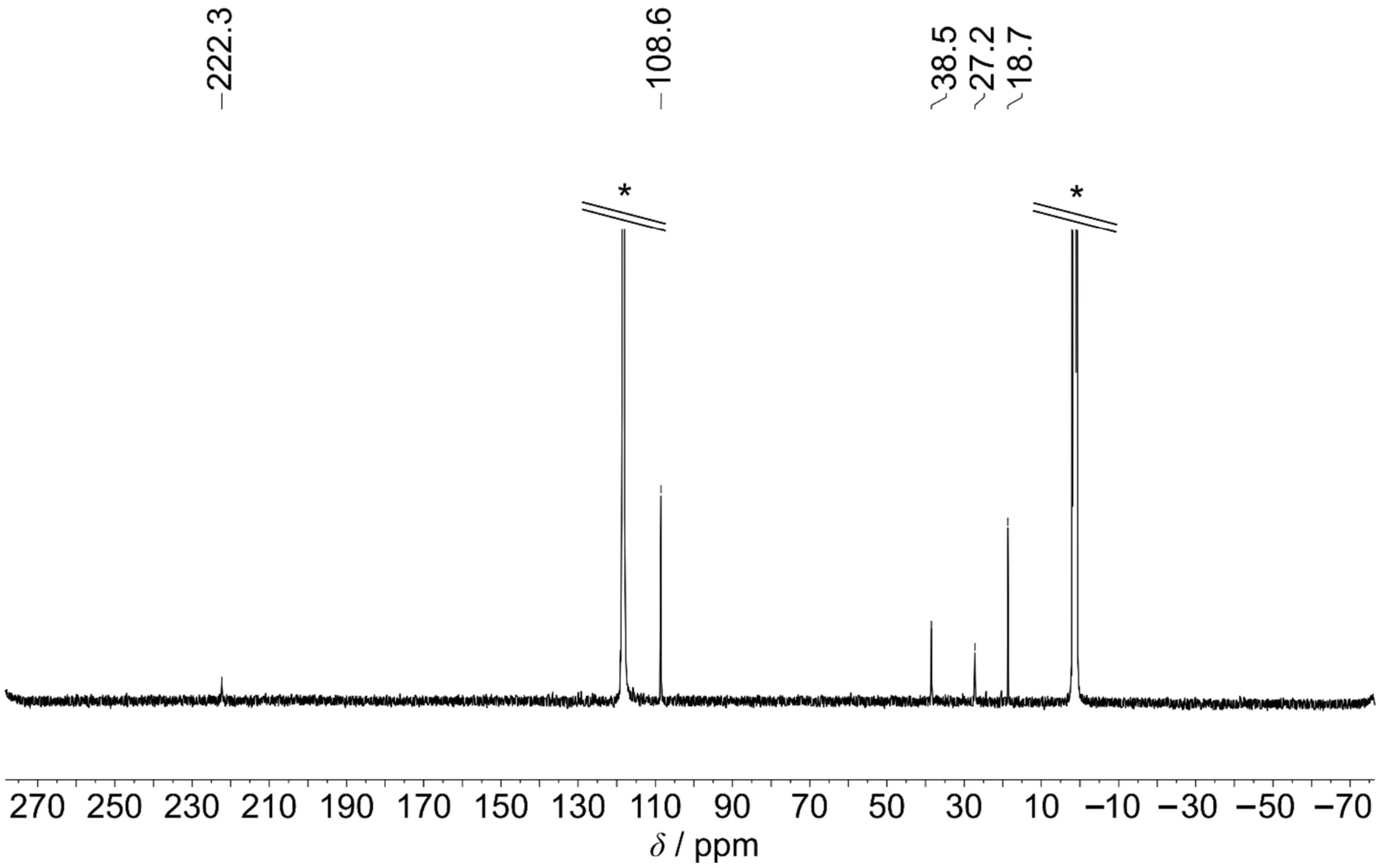

Supplementary Figure 9. $^{13}C\{^1H\}$ NMR spectrum of **1** $[Mn(pbmi)_2][PF_6]_2$ in CD_3CN at 298 K. The asterisks denote residual solvent resonances.

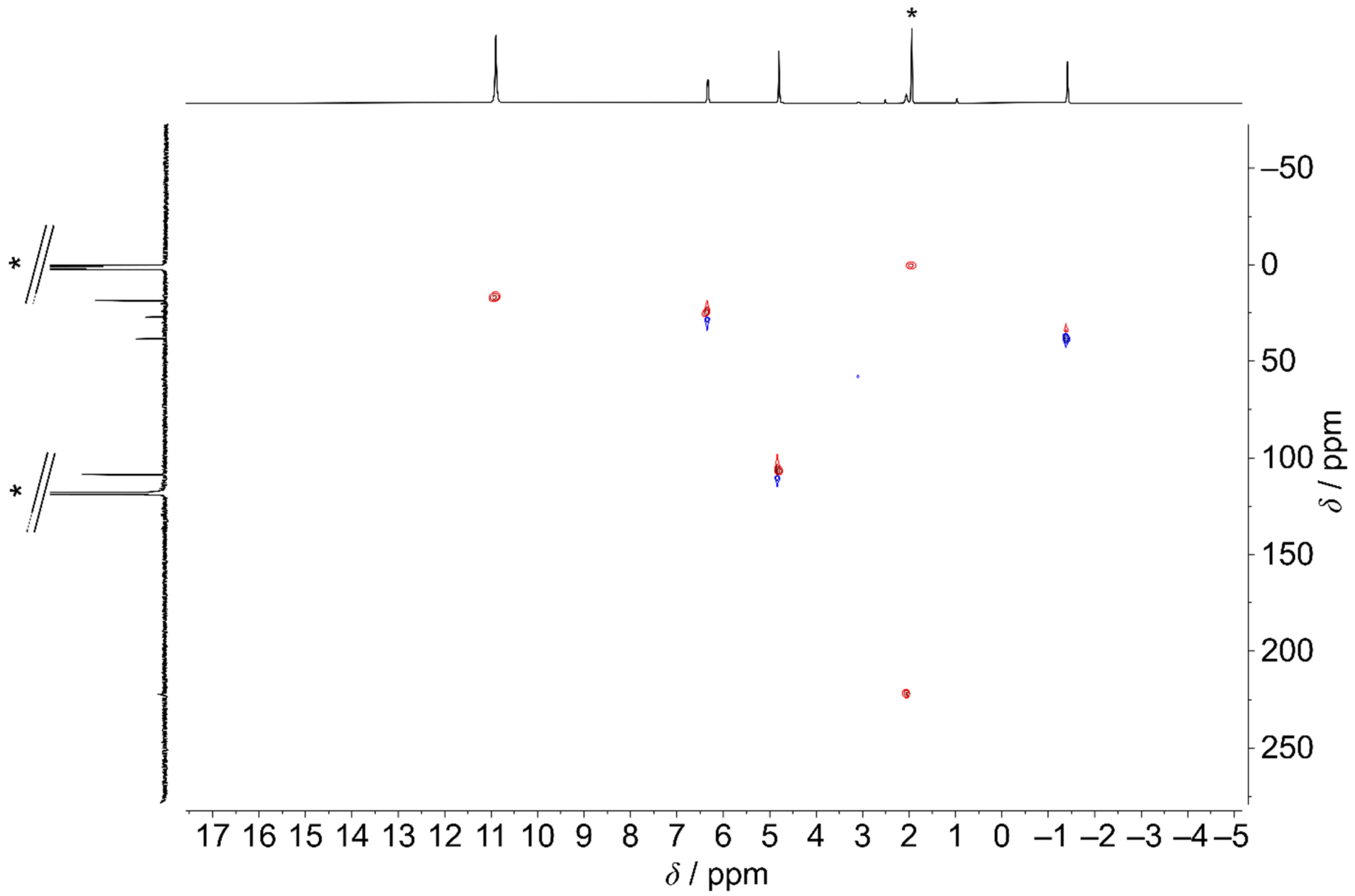

Supplementary Figure 10. ^{1}H, ^{13}C HSQC NMR spectrum of **1** [Mn(pbmi)$_2$][PF$_6$]$_2$ in CD$_3$CN at 298 K. The asterisks denote residual solvent resonances.

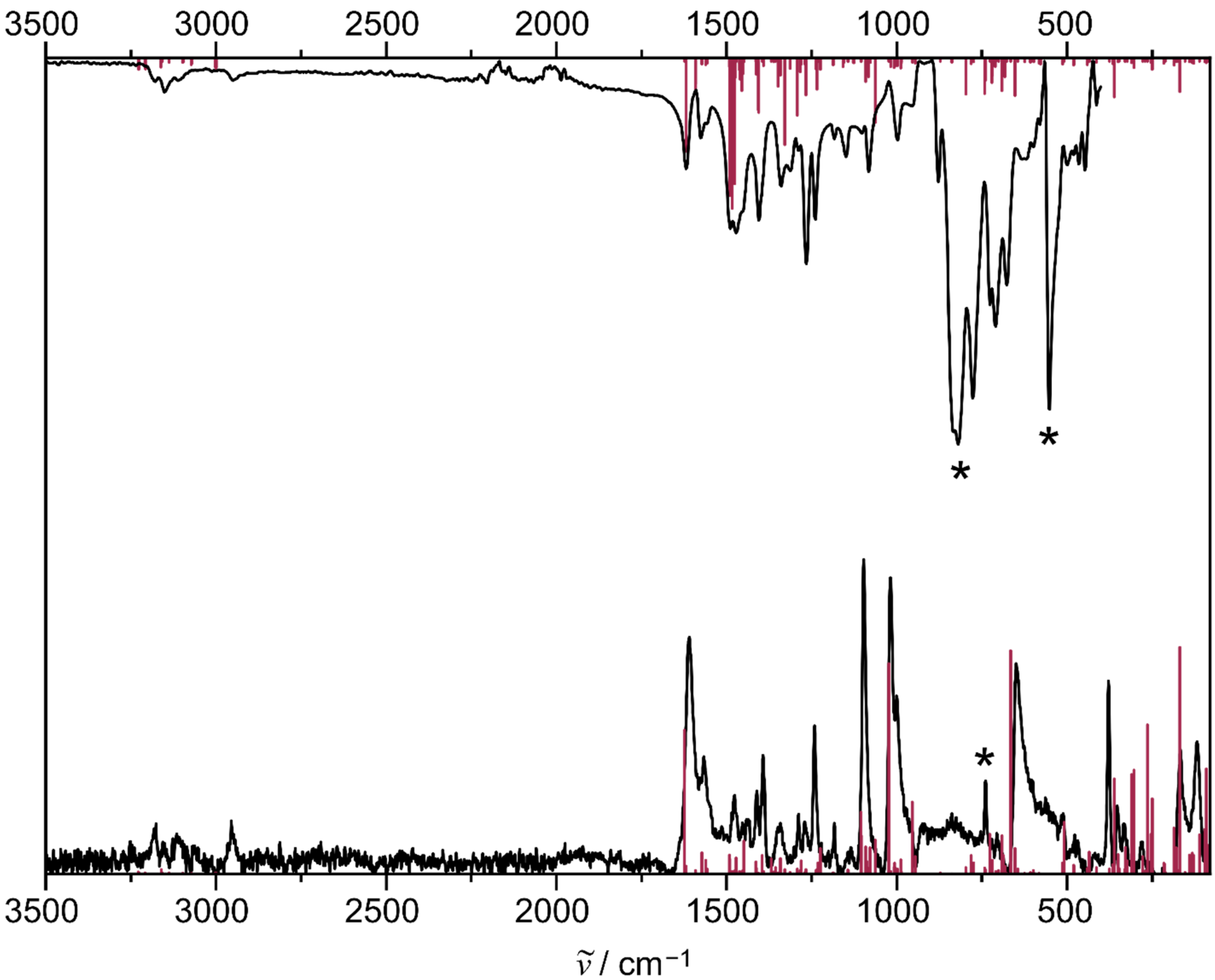

Supplementary Figure 11. Infrared (top) and Raman (bottom) spectra of crystalline **1** $[Mn(pbmi)_2][PF_6]_2$ at 293 K. DFT calculated Raman and IR vibrational bands are scaled with a factor of 0.98 and are depicted as red vertical bars. Asterisks denote absorptions of the hexafluorophosphate counterions.

Supplementary Table 5. Comparison of experimentally determined and DFT-calculated infrared transitions of **LS-1** $[Mn(pbmi)_2][PF_6]_2$. In the DFT calculation, the $[PF_6]^-$ counterions were omitted. Calculated energies were scaled by a factor of 0.98. Experimental intensities are denoted as very strong (vs), strong (s), medium (m) and weak (w), while calculated intensities are represented by the oscillator strength *f*.

**Experimental [cm^{-1}] (intensity)**	**Calculated [cm^{-1}]**	**Scaled by 0.98 [cm^{-1}]**	***f***
3179 (w)	3293	3227	13.0
3151 (w)	3273	3208	14.5
3113 (w)	3226	3161	10.4
2951 (w)	3060/3062	3001/2999	14.3/11.3
1619 (m)	1652	1619	166
1576 (m)	1623	1591	60.2
1558 (m)	1594	1562	6.35
1489 (s)	1521	1490	248
1472 (s)	1514/1506	1483/1476	272/226
1451 (s)	1485	1455	54.6
1405 (s)	1436/1435	1407/1406	90.3/94.5
1340 (m)	1357	1330	154
1312 (m)	1341	1314	13.3
1289 (m)	1319	1293	99.8
1265 (s)	1294	1268	62.7
1241 (s)	1260	1235	51.8
1185 (m)	1212/1210	1188/1186	6.12/6.19
1148 (m)	1180	1156	1.88
1101 (m)	1128	1105	0.04
1082 (m)	1114/1109/1107	1092/1087/1085	37.4/3.8/24.0
997 (m)	1007	987	0.80
954 (m)	975	956	1.96
877 (m)	890	872	0.12
818 (PF_6) (vs)	-	-	-
777 (vs)	813	797	60.8
726 (s)	736/735	721/720	38.9/26.0
710 (s)	725/724/716	711/710/702	10.2/0.01/0.17
676 (vs)	706/701/699/697	692/687/685/683	54.7/10.5/10.6/28.5
618 (m)	638	625	0.570
600 (m)	611	599	0.610
579 (m)	590	578	5.14
553 (PF_6) (vs)			
500 (m)	520	510	0.100
466 (m)	490	480	6.98
447 (m)	449	440	5.92

Supplementary Table 6. Comparison of experimentally determined and DFT-calculated Raman transitions of **LS-1** $[Mn(pbmi)_2][PF_6]_2$. In the DFT calculation, the $[PF_6]^-$ counterions were omitted. Calculated energies were scaled by factor of 0.98. Experimental intensities are denoted as very strong (vs), strong (s), medium (m) and weak (w), while calculated Raman intensities are given for comparison.

**Experimental [cm^{-1}] (intensity)**	**Calculated [cm^{-1}]**	**Scaled by 0.98 [cm^{-1}]**	**Calculated Raman intensity**
118 (m)	114	112	11959
167 (m)	173	170	69699
333 (w)	355	348	1696
363 (w)	358	351	5994
378 (s)	369	361	29158
649 (s)	680	666	68665
739 (PF_6) (m)	-	-	-
1000 (s)	1008	988	4369
1017 (vs)	1045	1024	64554
1097 (vs)	1127/1128	1104/1106	11701/18923
1183 (w)	1210/1212	1186/1188	236/288
1241 (m)	1250	1225	7872
1271 (w)	1294	1268	1349
1288 (w)	1319	1292	1426
1342 (w)	1369	1342	4757
1392 (m)	1424	1396	5643
1411 (m)	1442/1443	1413/1414	1831/3662
1441 (w)	1479	1450	10116
1476 (m)	1521/1522	1490/1492	4609/5716
1567 (m)	1605	1573	6540
1609 (s)	1656	1623	44192
2954 (w)	3062/3063	3001/3002	343/300
3179 (w)	3226	3161	1312

Supplementary Table 7. Charge transfer number analysis of the 50 lowest energy spin-allowed transitions of **LS-1** calculated by Loewdin population analysis with the complex cation fragmented into manganese (Mn), pyridines (L) and NHCs (L'). Difference electron densities (red = electron loss; blue = electron gain; displayed at an isosurface value of 0.003 a.u.) of the 20 lowest spin-allowed transitions of LS-1 with the respective calculated wavelengths (λ_{calcd}), wavelengths shifted by 0.4 eV to lower energies ($\lambda_{shifted}$), oscillator strengths (f) and dominant character. The transitions #1 and #2, representing the splitting of the triply degenerate T_2 ground state (in octahedral symmetry), were not shifted.

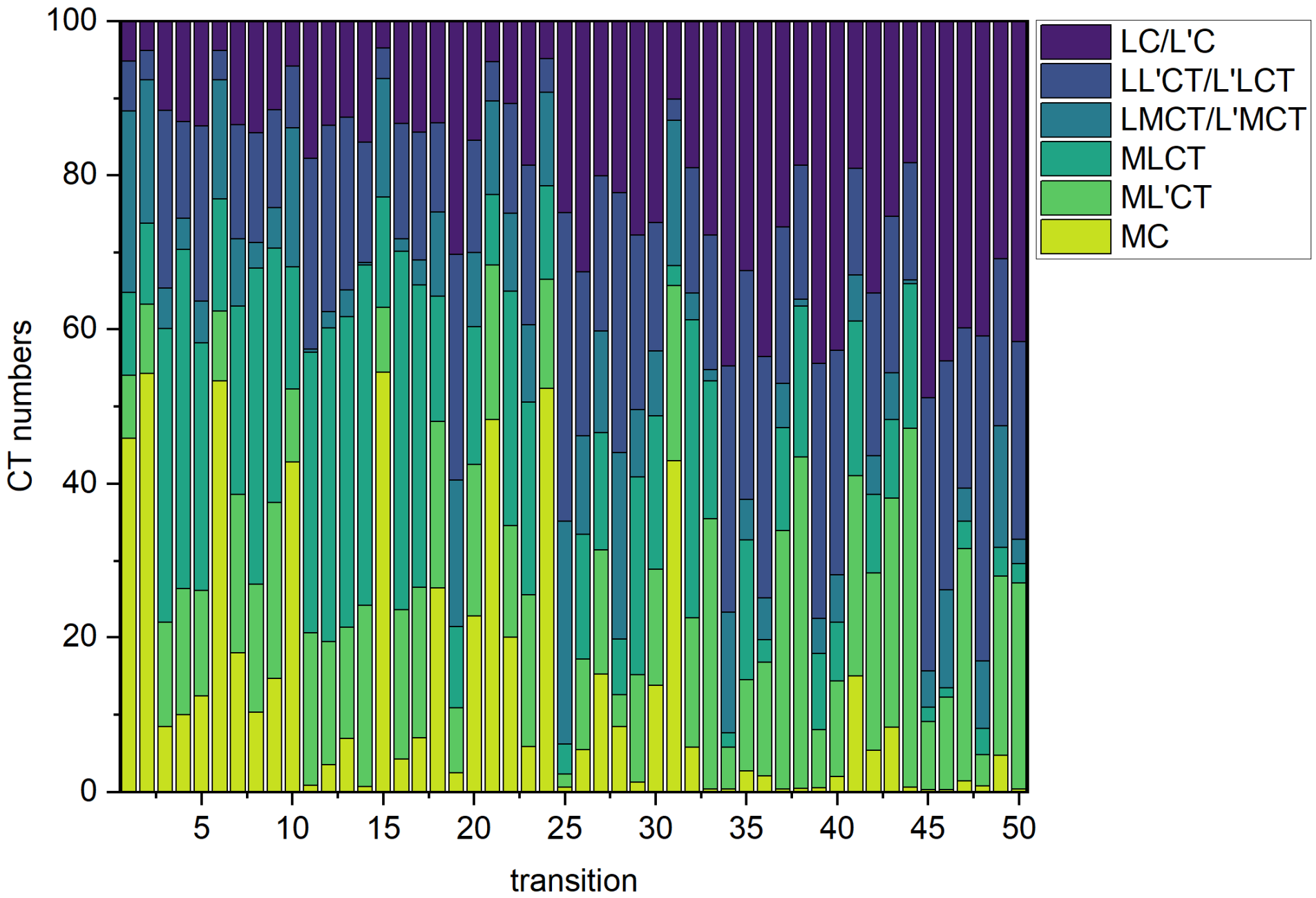

No.	TDDFT calculated difference density (isosurface value 0.003 a.u.)	λ_{calcd} / nm	$\lambda_{shifted}$ / nm (shifted by 0.4 eV to lower energy)[a]	oscillator strength f	Character
1	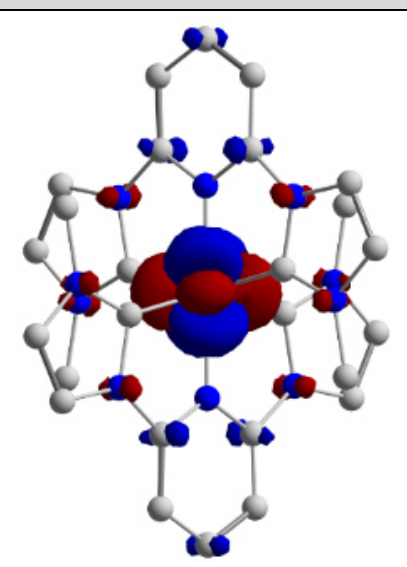	3763.1	-	1.08E–03	MLCT d_{Mn}-p_{py} (10.8%)<br>ML'CT d_{Mn}-p_{NHC} (8.16%)<br>L'MCT/ LMCT p_L-d_{Mn} (23.5%)<br>L'LCT/LL'CT $p_{L'}$-p_L (6.46%)<br>L'C/LC p_L-p_L, $p_{L'}$-$p_{L'}$ (5.16%)<br>**MC d_{Mn}-d_{Mn} (45.9%)**
2	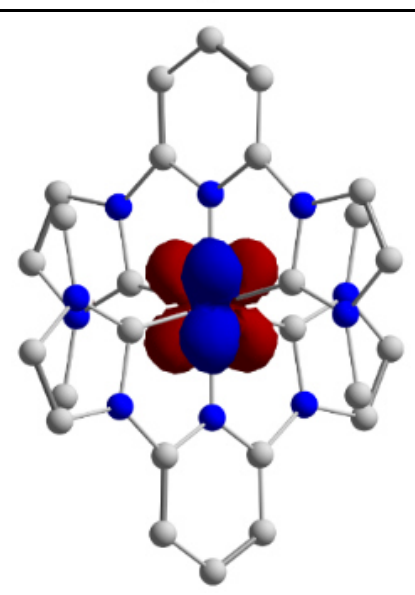	3223.1	-	4.47E–03	MLCT d_{Mn}-p_{py} (10.6%)<br>ML'CT d_{Mn}-p_{NHC} (8.96%)<br>L'MCT/ LMCT p_L-d_{Mn} (18.5%)<br>L'LCT/LL'CT $p_{L'}$-p_L (3.79%)<br>L'C/LC p_L-p_L, $p_{L'}$-$p_{L'}$ (3.85%)<br>**MC d_{Mn}-d_{Mn} (54.3%)**

3	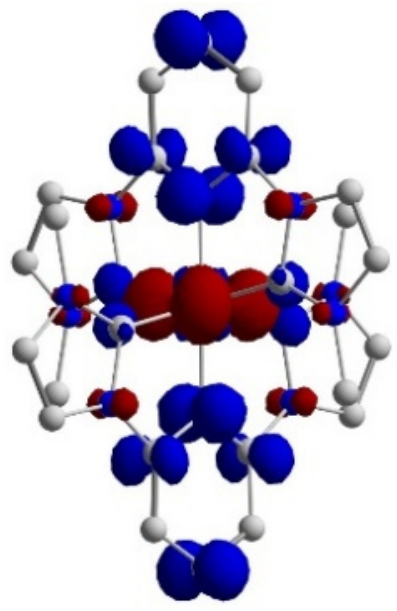	606.8	754.5	1.70E–03	**MLCT d_{Mn}-p_{py} (38.1%)**<br>ML’CT d_{Mn}-p_{NHC} (13.5%)<br>L’MCT/ LMCT p_{L}-d_{Mn} (5.35%)<br>L’LCT/LL’CT $p_{L'}$-p_{L} (23.0%)<br>L’C/LC p_{L}-p_{L}, $p_{L'}$-$p_{L'}$ (11.5%)<br>MC d_{Mn}-d_{Mn} (8.48%)
4	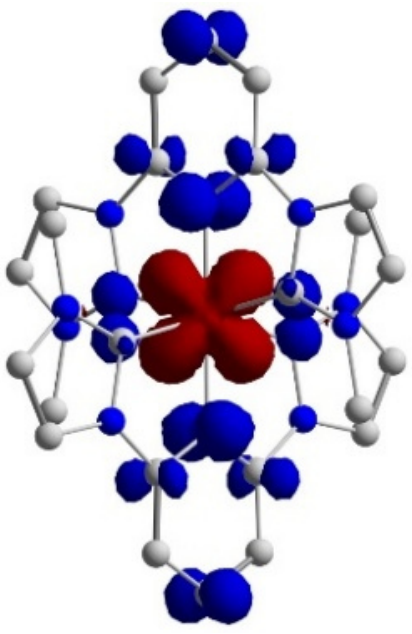	567.4	694.5	4.72E–06	**MLCT d_{Mn}-p_{py} (44.0%)**<br>ML’CT d_{Mn}-p_{NHC} (16.4%)<br>L’MCT/ LMCT p_{L}-d_{Mn} (4.05%)<br>L’LCT/LL’CT $p_{L'}$-p_{L} (12.5%)<br>L’C/LC p_{L}-p_{L}, $p_{L'}$-$p_{L'}$ (13.0%)<br>MC d_{Mn}-d_{Mn} (10.0%)
5	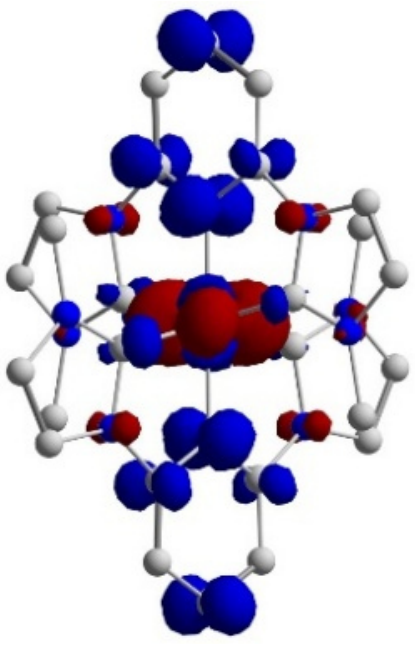	545.9	662.6	3.21E–03	**MLCT d_{Mn}-p_{py} (32.1%)**<br>ML’CT d_{Mn}-p_{NHC} (13.7%)<br>L’MCT/ LMCT p_{L}-d_{Mn} (5.36%)<br>L’LCT/LL’CT $p_{L'}$-p_{L} (22.8%)<br>L’C/LC p_{L}-p_{L}, $p_{L'}$-$p_{L'}$ (13.6%)<br>MC d_{Mn}-d_{Mn} (12.4%)
6	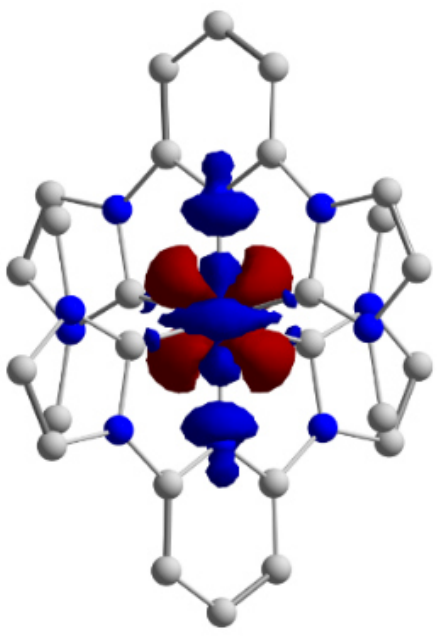	543.2	658.6	3.64E–04	MLCT d_{Mn}-p_{py} (14.6%)<br>ML’CT d_{Mn}-p_{NHC} (9.06%)<br>L’MCT/ LMCT p_{L}-d_{Mn} (15.4%)<br>L’LCT/LL’CT $p_{L'}$-p_{L} (3.85%)<br>L’C/LC p_{L}-p_{L}, $p_{L'}$-$p_{L'}$ (3.80%)<br>**MC d_{Mn}-d_{Mn} (53.3%)**
7	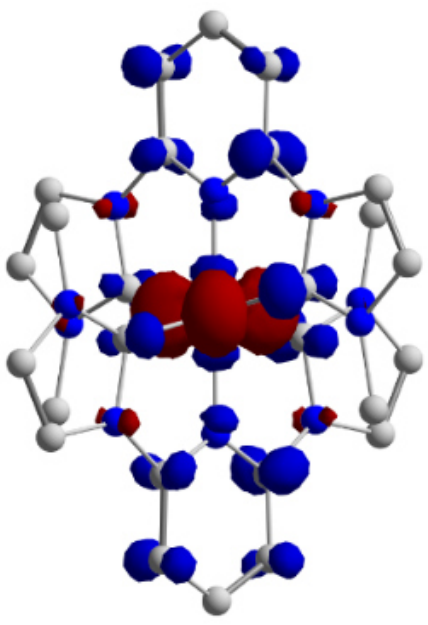	538.3	651.4	3.91E–05	**MLCT d_{Mn}-p_{py} (24.4%)**<br>ML’CT d_{Mn}-p_{NHC} (20.5%)<br>L’MCT/ LMCT p_{L}-d_{Mn} (8.79%)<br>L’LCT/LL’CT $p_{L'}$-p_{L} (14.8%)<br>L’C/LC p_{L}-p_{L}, $p_{L'}$-$p_{L'}$ (13.4%)<br>MC d_{Mn}-d_{Mn} (18.0%)

8	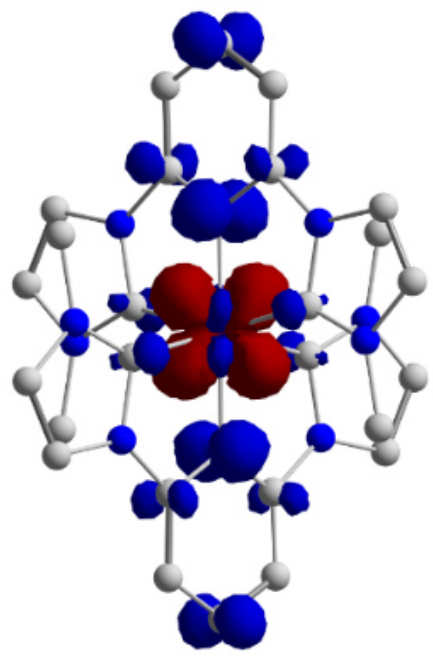	507.3	606.6	2.40E–02	**MLCT d_{Mn}-p_{py} (41.1%)**<br>ML’CT d_{Mn}-p_{NHC} (16.6%)<br>L’MCT/ LMCT p_{L}-d_{Mn} (3.25%)<br>L’LCT/LL’CT $p_{L'}$-p_{L} (14.2%)<br>L’C/LC p_{L}-p_{L}, $p_{L'}$-$p_{L'}$ (14.5%)<br>MC d_{Mn}-d_{Mn} (10.3%)
9	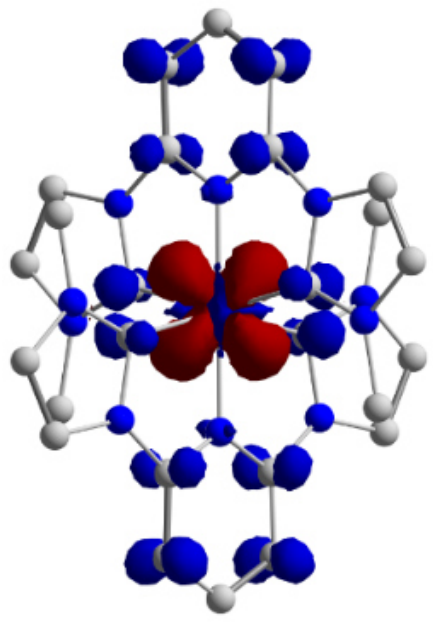	485.1	575.1	3.15E–03	**MLCT d_{Mn}-p_{py} (33.1%)**<br>ML’CT d_{Mn}-p_{NHC} (22.9%)<br>L’MCT/ LMCT p_{L}-d_{Mn} (5.20%)<br>L’LCT/LL’CT $p_{L'}$-p_{L} (12.7%)<br>L’C/LC p_{L}-p_{L}, $p_{L'}$-$p_{L'}$ (11.5%)<br>MC d_{Mn}-d_{Mn} (14.7%)
10	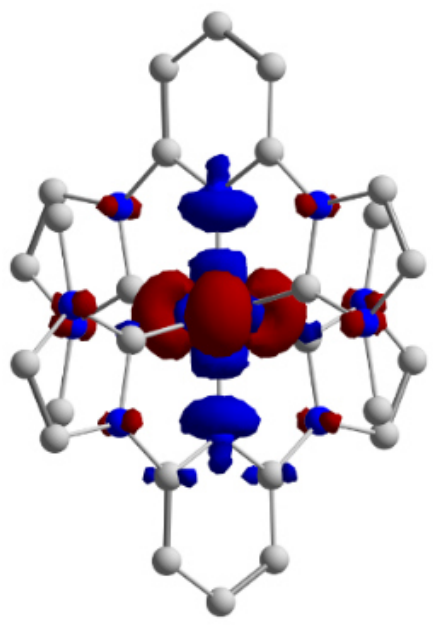	480.6	568.8	1.22E–04	MLCT d_{Mn}-p_{py} (15.9%)<br>ML’CT d_{Mn}-p_{NHC} (9.42%)<br>L’MCT/ LMCT p_{L}-d_{Mn} (18.0%)<br>L’LCT/LL’CT $p_{L'}$-p_{L} (8.02%)<br>L’C/LC p_{L}-p_{L}, $p_{L'}$-$p_{L'}$ (5.81%)<br>**MC d_{Mn}-d_{Mn} (42.8%)**
11	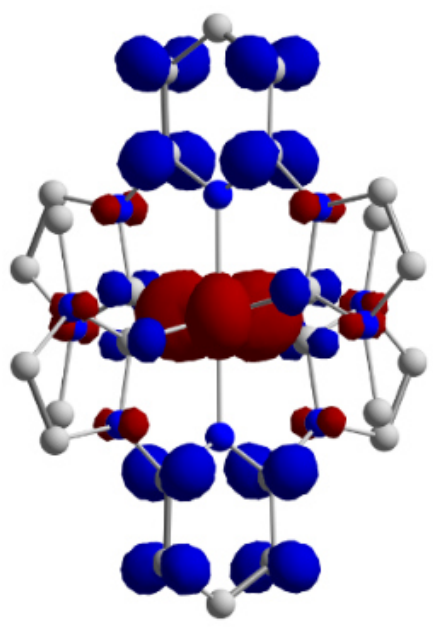	479.3	567.0	3.98E–02	**MLCT d_{Mn}-p_{py} (36.4%)**<br>ML’CT d_{Mn}-p_{NHC} (19.8%)<br>L’MCT/LMCT p_{L}-d_{Mn} (0.469%)<br>L’LCT/LL’CT $p_{L'}$-p_{L} (24.8%)<br>L’C/LC p_{L}-p_{L}, $p_{L'}$-$p_{L'}$ (17.8%)<br>MC d_{Mn}-d_{Mn} (0.858%)
12	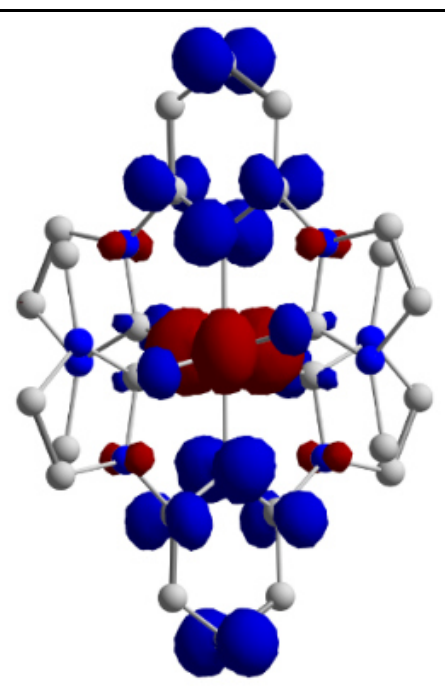	462.8	544.0	3.90E–03	**MLCT d_{Mn}-p_{py} (40.7%)**<br>ML’CT d_{Mn}-p_{NHC} (16.0%)<br>L’MCT/ LMCT p_{L}-d_{Mn} (2.04%)<br>L’LCT/LL’CT $p_{L'}$-p_{L} (24.3%)<br>L’C/LC p_{L}-p_{L}, $p_{L'}$-$p_{L'}$ (13.5%)<br>MC d_{Mn}-d_{Mn} (3.51%)

13	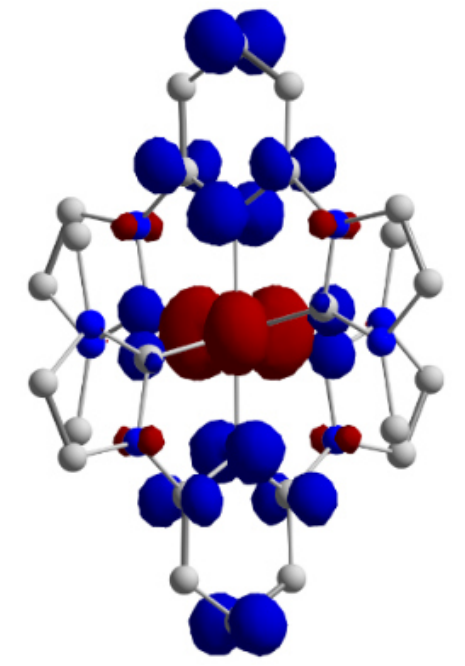	450.4	527.0	2.00E–03	**MLCT d_{Mn}-p_{py} (40.3%)**<br>ML'CT d_{Mn}-p_{NHC} (14.3%)<br>L'MCT/ LMCT p_{L}-d_{Mn} (3.53%)<br>L'LCT/LL'CT $p_{L'}$-p_{L} (22.3%)<br>L'C/LC p_{L}-p_{L}, $p_{L'}$-$p_{L'}$ (12.5%)<br>MC d_{Mn}-d_{Mn} (6.93%)
14	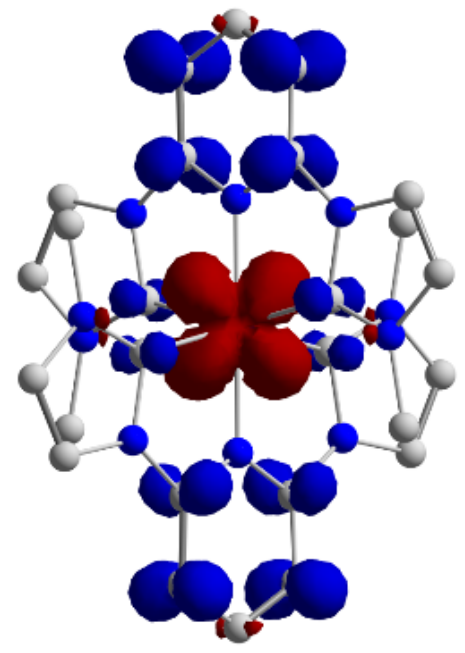	445.2	519.9	5.58E–03	**MLCT d_{Mn}-p_{py} (44.3%)**<br>ML'CT d_{Mn}-p_{NHC} (23.4%)<br>L'MCT/LMCT p_{L}-d_{Mn} (0.291%)<br>L'LCT/LL'CT $p_{L'}$-p_{L} (15.6%)<br>L'C/LC p_{L}-p_{L}, $p_{L'}$-$p_{L'}$ (15.6%)<br>MC d_{Mn}-d_{Mn} (0.748%)
15	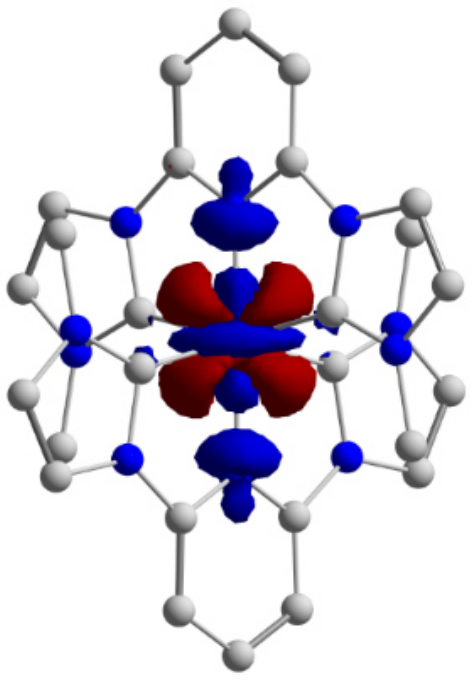	418.9	484.4	6.09E–04	MLCT d_{Mn}-p_{py} (14.4%)<br>ML'CT d_{Mn}-p_{NHC} (8.36%)<br>L'MCT/ LMCT p_{L}-d_{Mn} (15.4%)<br>L'LCT/LL'CT $p_{L'}$-p_{L} (3.98%)<br>L'C/LC p_{L}-p_{L}, $p_{L'}$-$p_{L'}$ (3.47%)<br>**MC d_{Mn}-d_{Mn} (54.4%)**
16	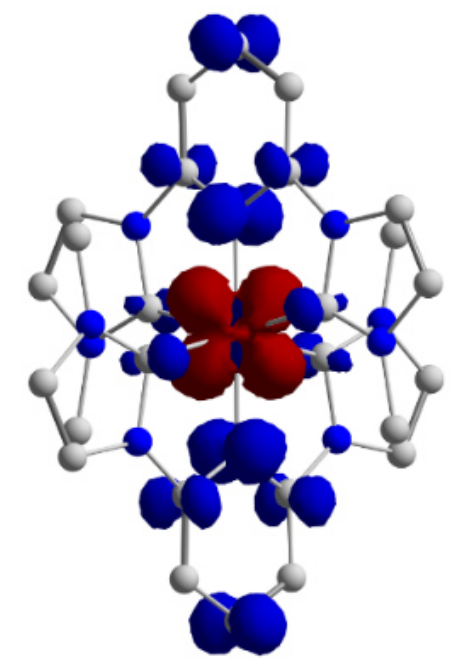	411.8	474.9	0.111	**MLCT d_{Mn}-p_{py} (46.5%)**<br>ML'CT d_{Mn}-p_{NHC} (19.3%)<br>L'MCT/ LMCT p_{L}-d_{Mn} (1.68%)<br>L'LCT/LL'CT $p_{L'}$-p_{L} (14.9%)<br>L'C/LC p_{L}-p_{L}, $p_{L'}$-$p_{L'}$ (13.3%)<br>MC d_{Mn}-d_{Mn} (4.28%)

17	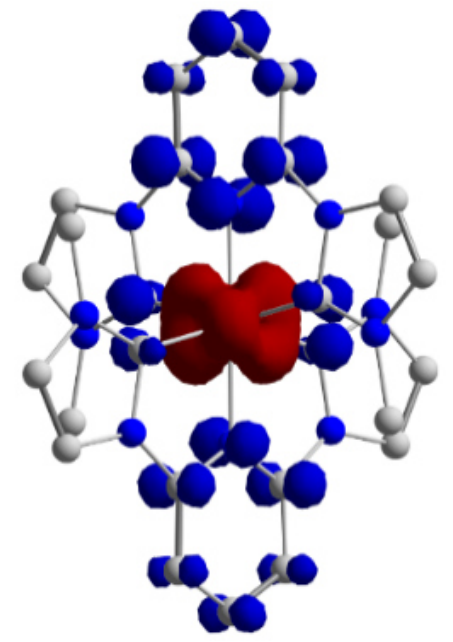	409.8	472.2	3.00E-4	**MLCT d_{Mn}-p_{py} (39.2%)**<br>ML'CT d_{Mn}-p_{NHC} (19.5%)<br>L'MCT/ LMCT p_{L}-d_{Mn} (3.27%)<br>L'LCT/LL'CT $p_{L'}$-p_{L} (16.5%)<br>L'C/LC p_{L}-p_{L}, $p_{L'}$-$p_{L'}$ (14.4%)<br>MC d_{Mn}-d_{Mn} (7.03%)
18	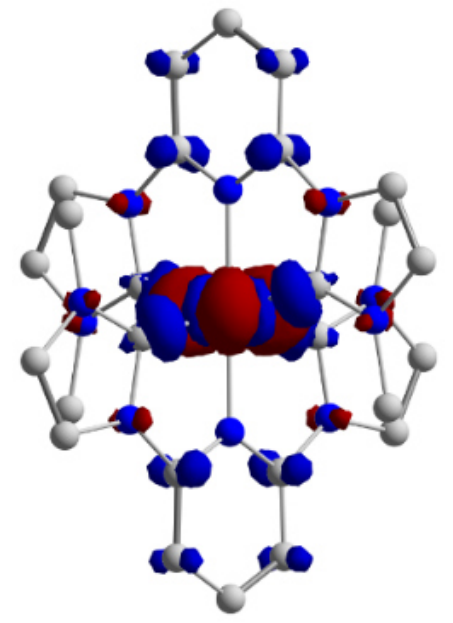	383.3	437.4	5.0E-02	MLCT d_{Mn}-p_{py} (16.3%)<br>ML'CT d_{Mn}-p_{NHC} (21.5%)<br>L'MCT/ LMCT p_{L}-d_{Mn} (10.9%)<br>L'LCT/LL'CT $p_{L'}$-p_{L} (11.6%)<br>L'C/LC p_{L}-p_{L}, $p_{L'}$-$p_{L'}$ (13.2%)<br>**MC d_{Mn}-d_{Mn} (26.5%)**
19	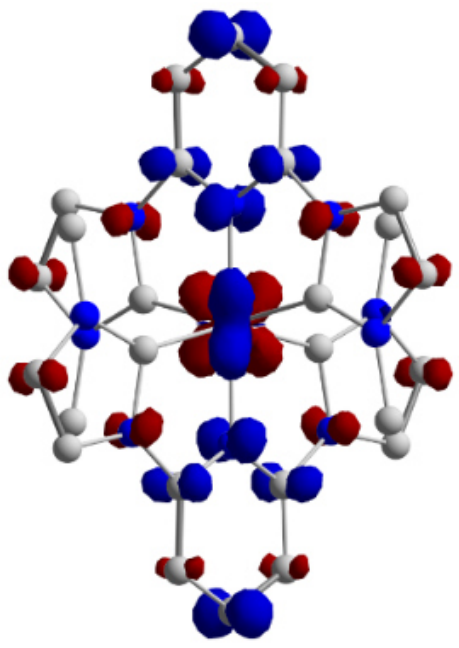	380.6	433.9	4.16E-04	MLCT d_{Mn}-p_{py} (10.5%)<br>ML'CT d_{Mn}-p_{NHC} (8.37%)<br>L'MCT/ LMCT p_{L}-d_{Mn} (19.0%)<br>L'LCT/LL'CT $p_{L'}$-p_{L} (29.3%)<br>**L'C/LC p_{L}-p_{L}, $p_{L'}$-$p_{L'}$ (30.2%)**<br>MC d_{Mn}-d_{Mn} (2.53%)
20	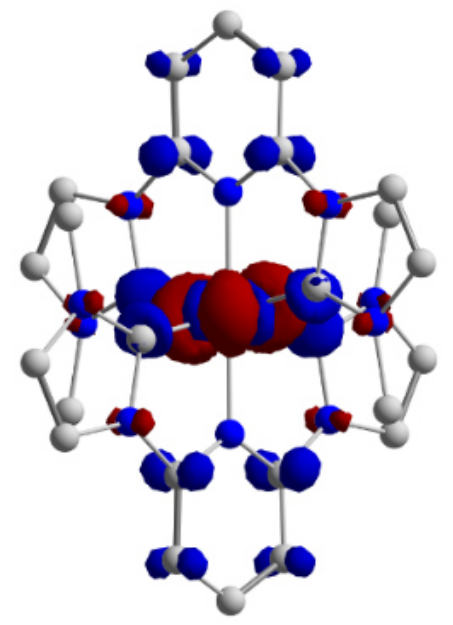	372.9	423.9	4.34E-02	MLCT d_{Mn}-p_{py} (17.8%)<br>ML'CT d_{Mn}-p_{NHC} (19.7%)<br>L'MCT/ LMCT p_{L}-d_{Mn} (9.76%)<br>L'LCT/LL'CT $p_{L'}$-p_{L} (14.5%)<br>L'C/LC p_{L}-p_{L}, $p_{L'}$-$p_{L'}$ (15.5%)<br>**MC d_{Mn}-d_{Mn} (22.8%)**

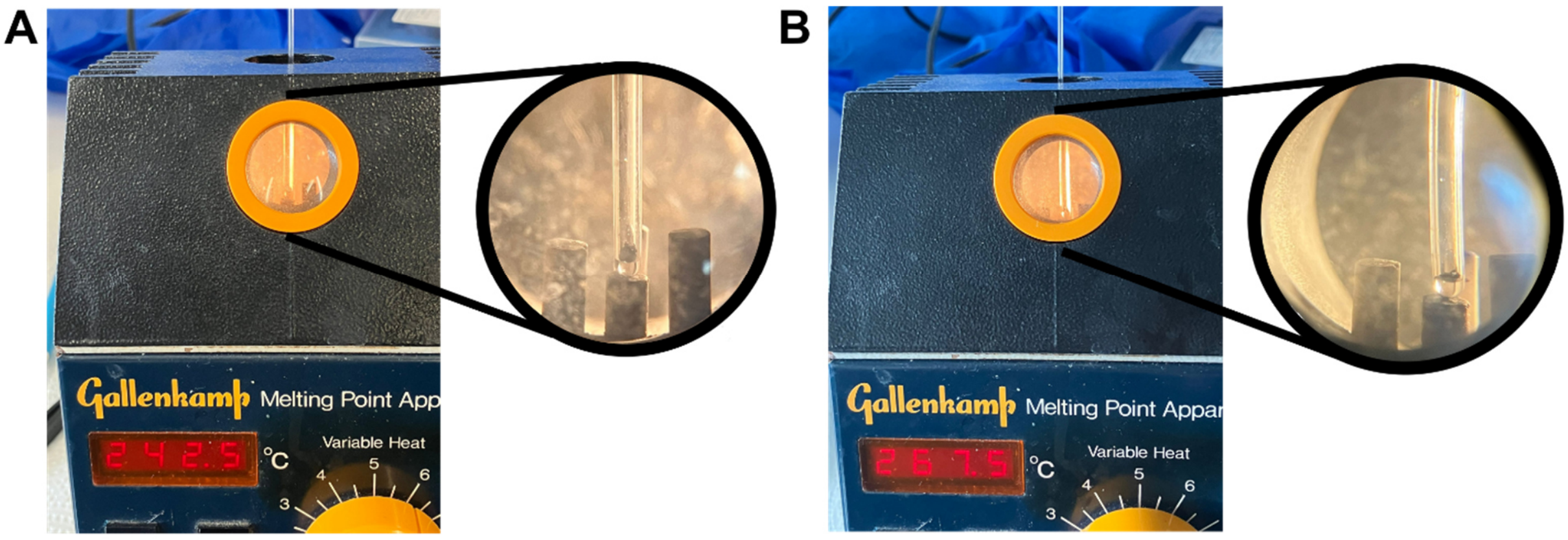

Supplementary Figure 12. Photograph of solid **1** after the temperature was increased to 515.5 K (**A**), appearing as a dark solid, which indicates the LS state. Solid **1** after the temperature was further increased to 540.5 K (**B**). The shrunk solid material indicates at least partial decomposition of **1** at these high temperatures, but no clear sign of conversion to the HS state.

Supplementary Table 8. Charge transfer number analysis of the 50 lowest energy spin-allowed transitions of **HS-1** calculated by Loewdin population analysis with the complex cation fragmented into manganese (Mn), pyridines (L) and NHCs (L'). Difference electron densities (red = electron loss; blue = electron gain; displayed at an isosurface value of 0.003 a.u.) of the 10 lowest spin-allowed transitions of **HS-1** with the respective calculated wavelengths (λ_{calcd}), wavelengths shifted by 0.1 eV to lower energies ($\lambda_{shifted}$), oscillator strengths (f) and dominant character.

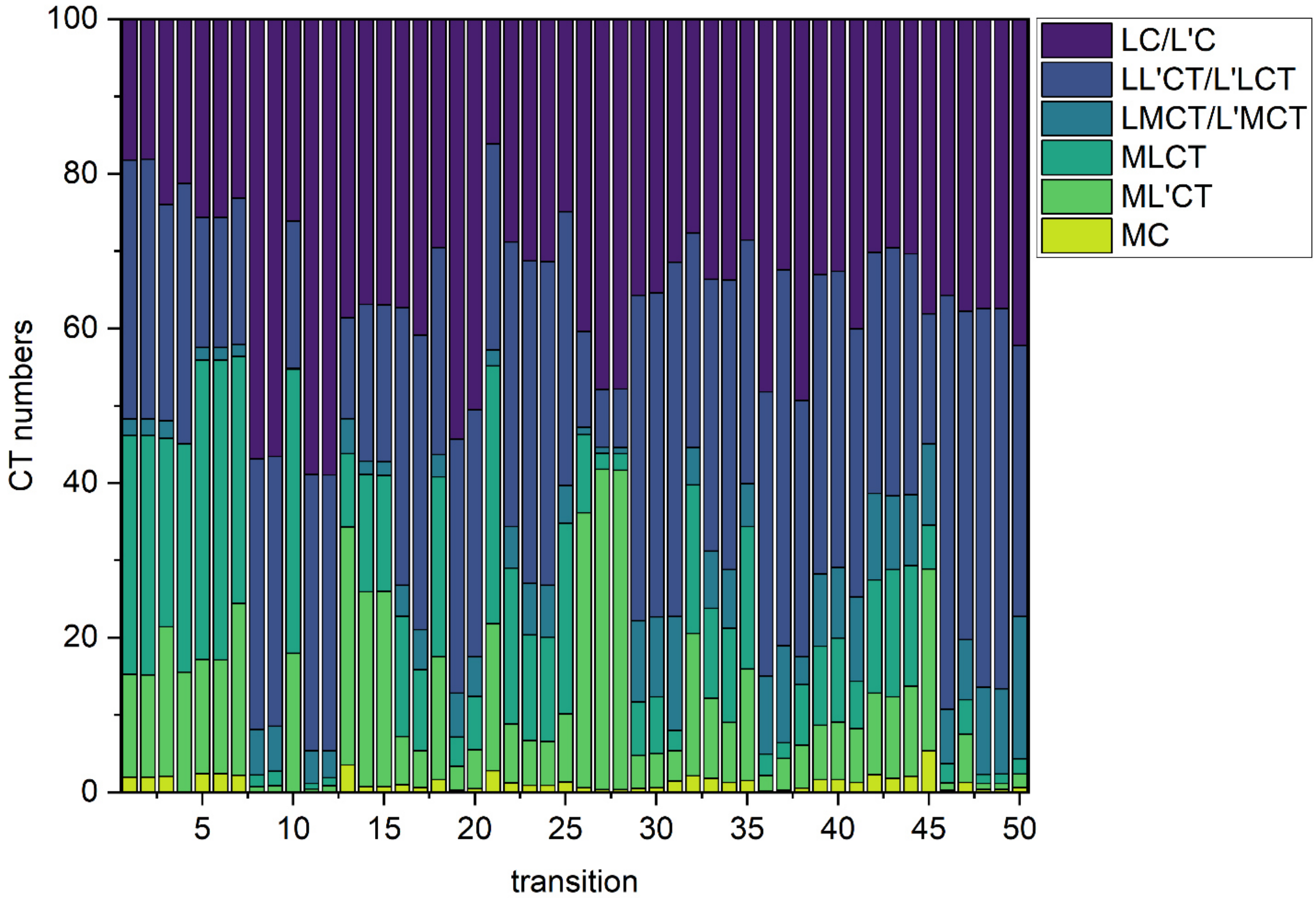

No.	TDDFT calculated difference density (isosurface value 0.003 a.u.)	λ_{calcd} / nm	$\lambda_{shifted}$ / nm (shifted by 0.1 eV to lower energy)	oscillator strength f	Character
1	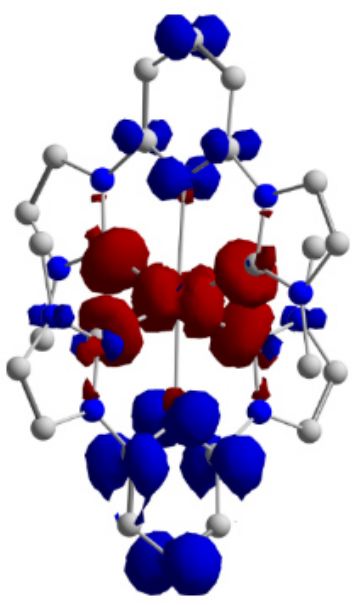	585.2	614.2	3.92E–03	MLCT d_{Mn}-p_{py} (31.0%)<br>ML'CT d_{Mn}-p_{NHC} (13.3%)<br>L'MCT/ LMCT p_{L}-d_{Mn} (2.11%)<br>**L'LCT/LL'CT $p_{L'}$-p_{L} (33.5%)**<br>L'C/LC p_{L}-p_{L}, $p_{L'}$-$p_{L'}$ (18.2%)<br>MC d_{Mn}-d_{Mn} (1.99%)

2	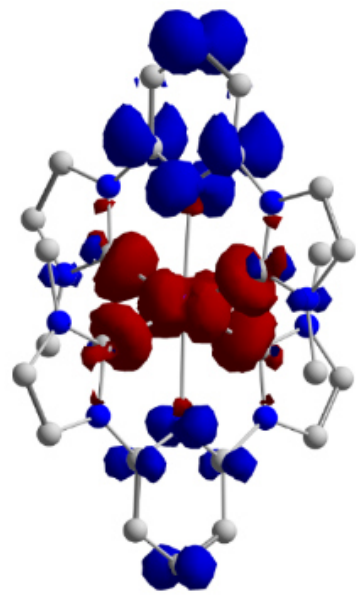	583.9	612.8	3.83E–03	MLCT d_{Mn}-p_{py} (31.1%)<br>ML’CT d_{Mn}-p_{NHC} (13.2%)<br>L’MCT/ LMCT p_{L}-d_{Mn} (2.10%)<br>**L’LCT/LL’CT $p_{L'}$-p_{L} (33.6%)**<br>L’C/LC p_{L}-p_{L}, $p_{L'}$-$p_{L'}$ (18.1%)<br>MC d_{Mn}-d_{Mn} (1.98%)
3	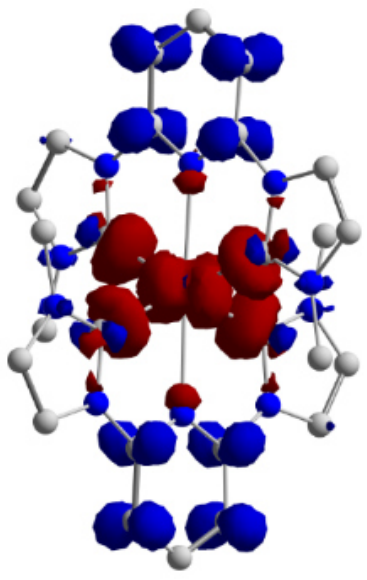	513.2	535.4	2.49E–05	MLCT d_{Mn}-p_{py} (24.4%)<br>ML’CT d_{Mn}-p_{NHC} (19.3%)<br>L’MCT/ LMCT p_{L}-d_{Mn} (2.26%)<br>**L’LCT/LL’CT $p_{L'}$-p_{L} (28.0%)**<br>L’C/LC p_{L}-p_{L}, $p_{L'}$-$p_{L'}$ (23.9%)<br>MC d_{Mn}-d_{Mn} (2.11%)
4	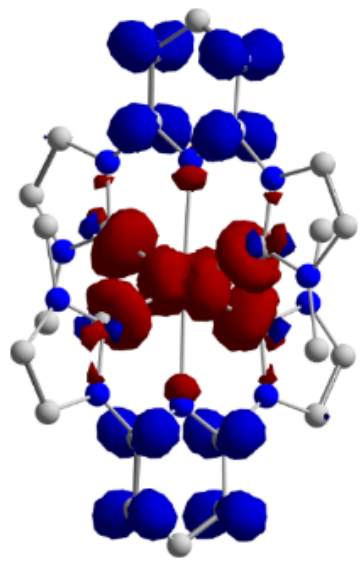	475.9	494.9	1.36E–05	MLCT d_{Mn}-p_{py} (29.5%)<br>ML’CT d_{Mn}-p_{NHC} (15.5%)<br>L’MCT/LMCT p_{L}-d_{Mn} (5.8E–4%)<br>**L’LCT/LL’CT $p_{L'}$-p_{L} (33.6%)**<br>L’C/LC p_{L}-p_{L}, $p_{L'}$-$p_{L'}$ (21.2%)<br>MC d_{Mn}-d_{Mn} (2.7E–4%)
5	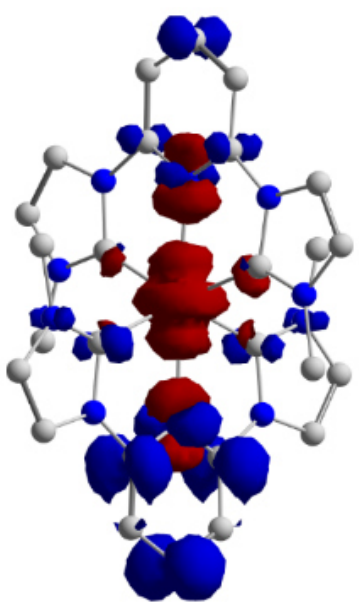	402.1	415.6	8.90E–04	**MLCT d_{Mn}-p_{py} (38.8%)**<br>ML’CT d_{Mn}-p_{NHC} (14.8%)<br>L’MCT/ LMCT p_{L}-d_{Mn} (1.64%)<br>L’LCT/LL’CT $p_{L'}$-p_{L} (16.8%)<br>L’C/LC p_{L}-p_{L}, $p_{L'}$-$p_{L'}$ (25.6%)<br>MC d_{Mn}-d_{Mn} (2.45%)
6	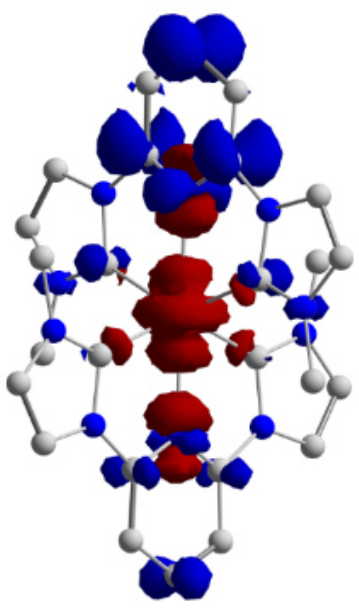	401.5	414.9	8.46E–04	**MLCT d_{Mn}-p_{py} (38.9%)**<br>ML’CT d_{Mn}-p_{NHC} (14.7%)<br>L’MCT/ LMCT p_{L}-d_{Mn} (1.63%)<br>L’LCT/LL’CT $p_{L'}$-p_{L} (16.8%)<br>L’C/LC p_{L}-p_{L}, $p_{L'}$-$p_{L'}$ (25.6%)<br>MC d_{Mn}-d_{Mn} (2.44%)

7	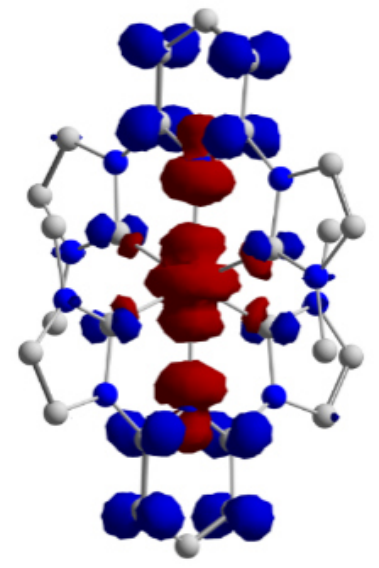	356.5	367.1	8.34E–07	**MLCT d_{Mn}-p_{py} (32.0%)**<br>ML’CT d_{Mn}-p_{NHC} (22.2%)<br>L’MCT/ LMCT p_{L}-d_{Mn} (1.51%)<br>L’LCT/LL’CT $p_{L'}$-p_{L} (18.9%)<br>L’C/LC p_{L}-p_{L}, $p_{L'}$-$p_{L'}$ (23.1%)<br>MC d_{Mn}-d_{Mn} (2.22%)
8	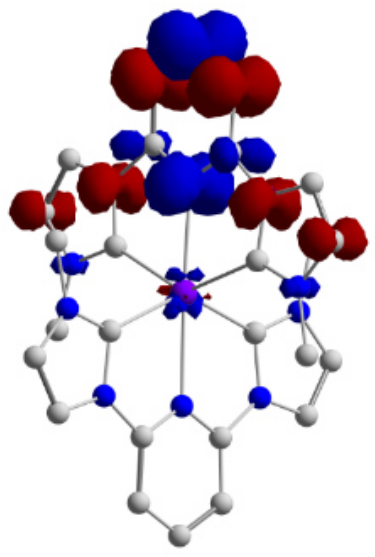	341.9	351.6	5.39E–04	MLCT d_{Mn}-p_{py} (1.53%)<br>ML’CT d_{Mn}-p_{NHC} (0.613%)<br>L’MCT/ LMCT p_{L}-d_{Mn} (5.87%)<br>L’LCT/LL’CT $p_{L'}$-p_{L} (35.0%)<br>**L’C/LC p_{L}-p_{L}, $p_{L'}$-$p_{L'}$ (56.9%)**<br>MC d_{Mn}-d_{Mn} (0.107%)
9	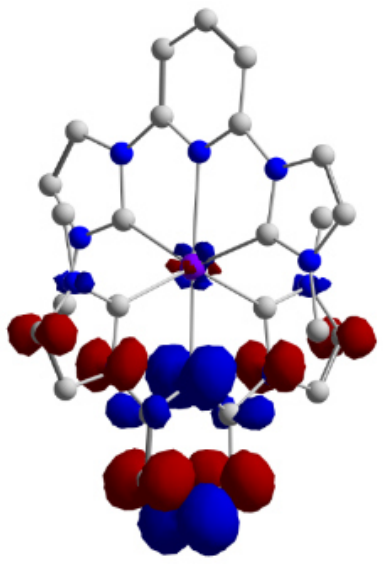	341.8	351.5	4.97E–04	MLCT d_{Mn}-p_{py} (1.87%)<br>ML’CT d_{Mn}-p_{NHC} (0.779%)<br>L’MCT/ LMCT p_{L}-d_{Mn} (5.80%)<br>L’LCT/LL’CT $p_{L'}$-p_{L} (34.9%)<br>**L’C/LC p_{L}-p_{L}, $p_{L'}$-$p_{L'}$ (56.6%)**<br>MC d_{Mn}-d_{Mn} (0.108%)
10	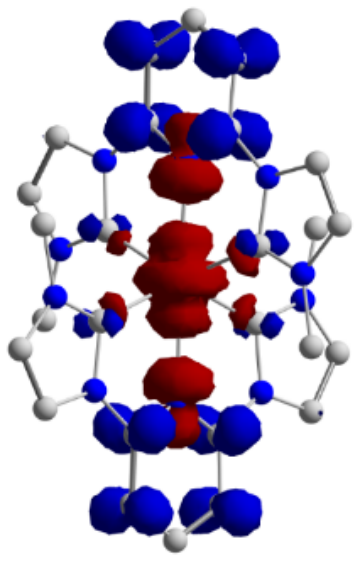	340.8	350.4	1.20E–05	**MLCT d_{Mn}-p_{py} (36.9%)**<br>ML’CT d_{Mn}-p_{NHC} (17.9%)<br>L’MCT/ LMCT p_{L}-d_{Mn} (0.114%)<br>L’LCT/LL’CT $p_{L'}$-p_{L} (19.0%)<br>L’C/LC p_{L}-p_{L}, $p_{L'}$-$p_{L'}$ (26.0%)<br>MC d_{Mn}-d_{Mn} (4.6 E–04%)

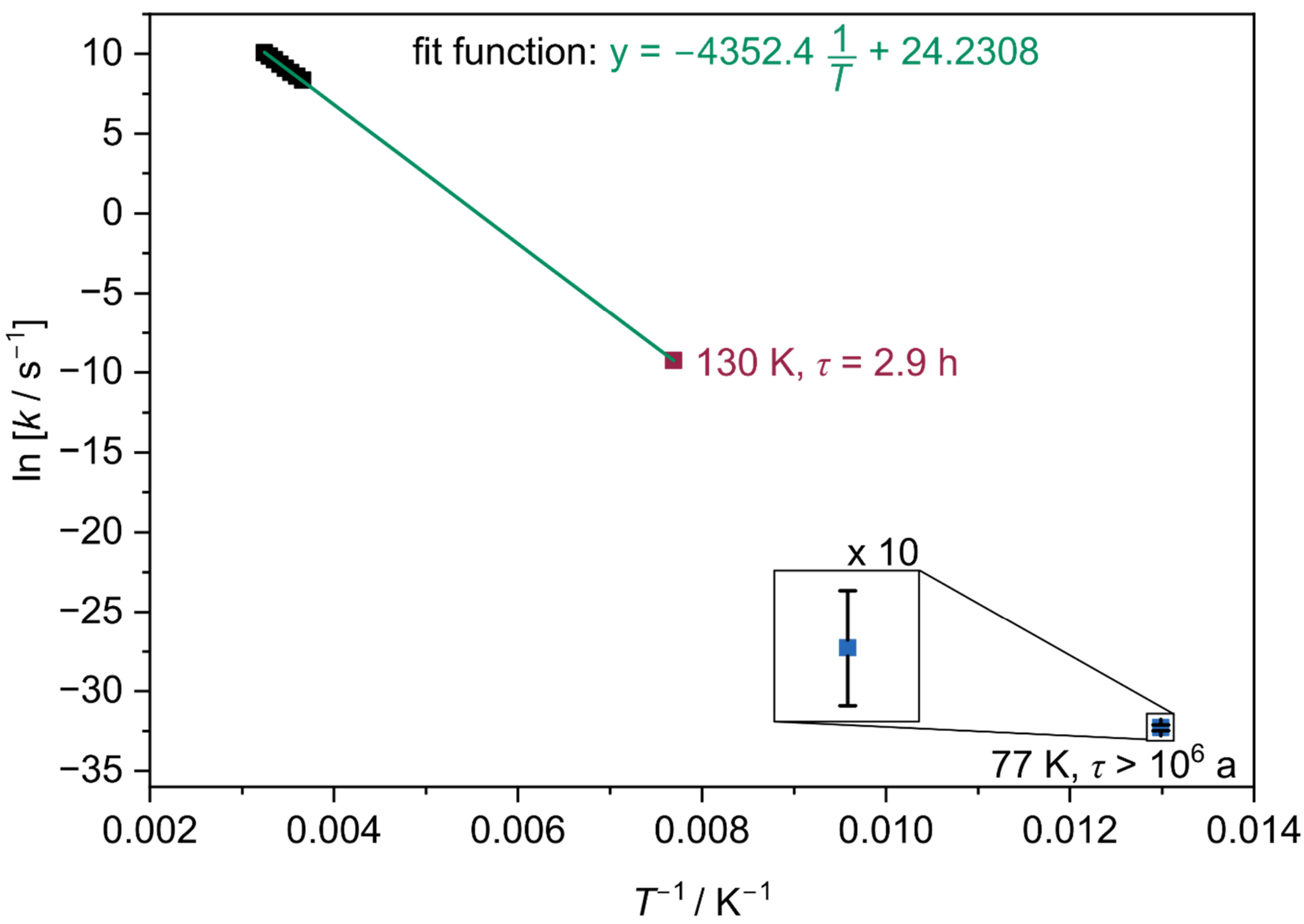

Supplementary Figure 13. Arrhenius plot (black and red squares) of experimentally determined rate constants for the HS → LS relaxation of **1** from TA (black squares) and absorption (red squares) spectroscopy in the temperature range of 130 K – 308 K of **1** in MeCN (TA, Fig. 2E) or 2-MeTHF/MeCN (29:1, *v/v,* absorption spectroscopy, Fig. 3A). The linear fit function is given in green. The lifetime at 77 K was extrapolated from the fit function (blue square) with the respective 95% prediction interval, which was calculated according to equation 4 (see Methods, calculation of prediction intervals) giving $\tau_{HL,min}(77\ K) = 2.8\times10^6$ a and $\tau_{HL,max}(77\ K) = 4.0\times10^6$ a.

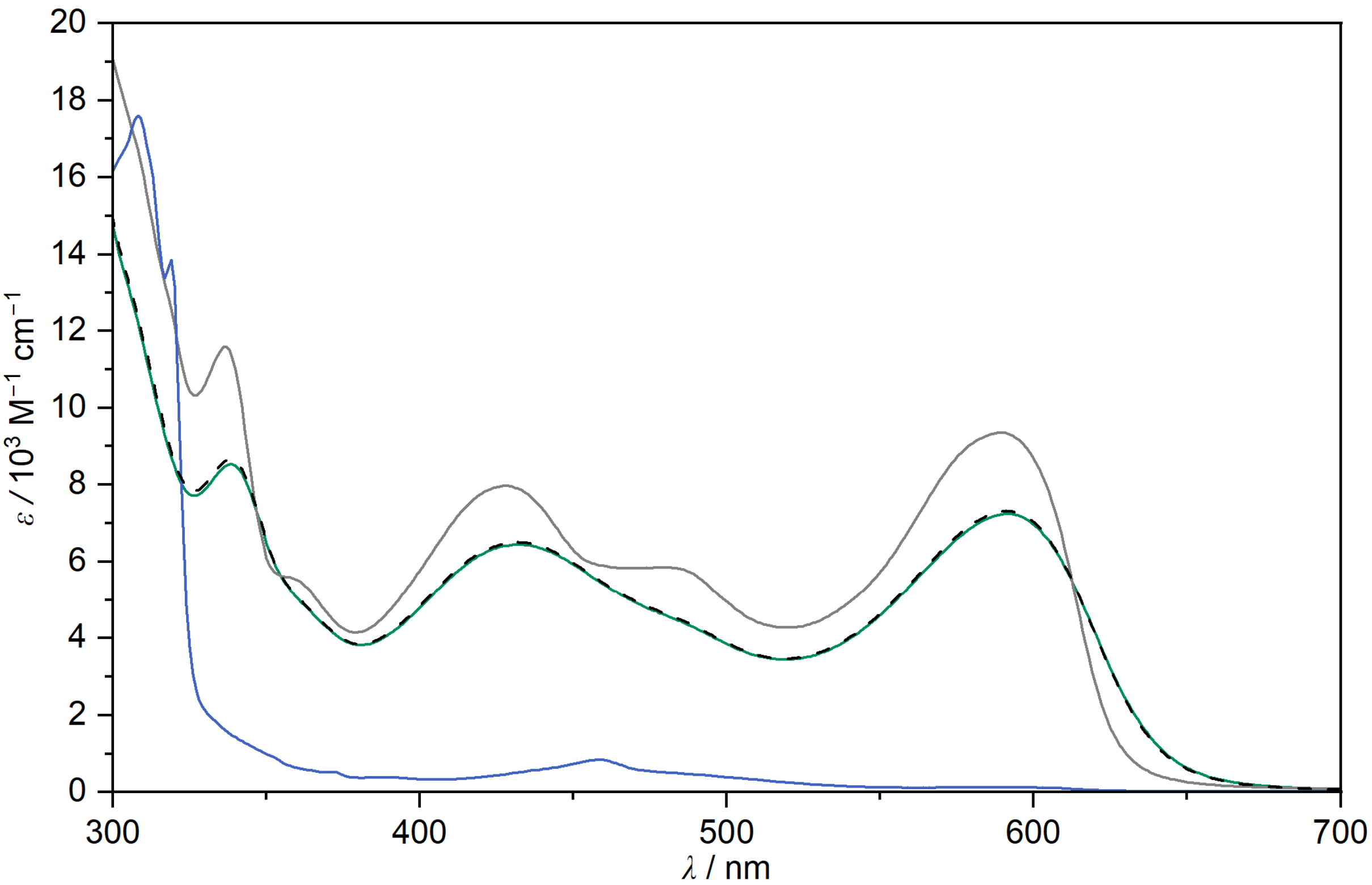

Supplementary Figure 14. Absorption spectra of **1** in 2-MeTHF/MeCN (29:1 *v*/*v*) before the T_{LIESST} experiment at 293 K (green) and at 95 K (grey), leading to a thermochromic shift and narrowing of the absorption bands upon cooling. This results in a visible color change of the **LS-1** complex from green to red. Irradiation of **LS-1** at λ_{exc} = 560 nm (2 minutes) at 95 K, results in **LS-1** to **HS-1** conversion (blue). The absorption spectrum after the T_{LIESST} experiment and warming to 293 K (black dashed line) confirms full recovery of **LS-1**.

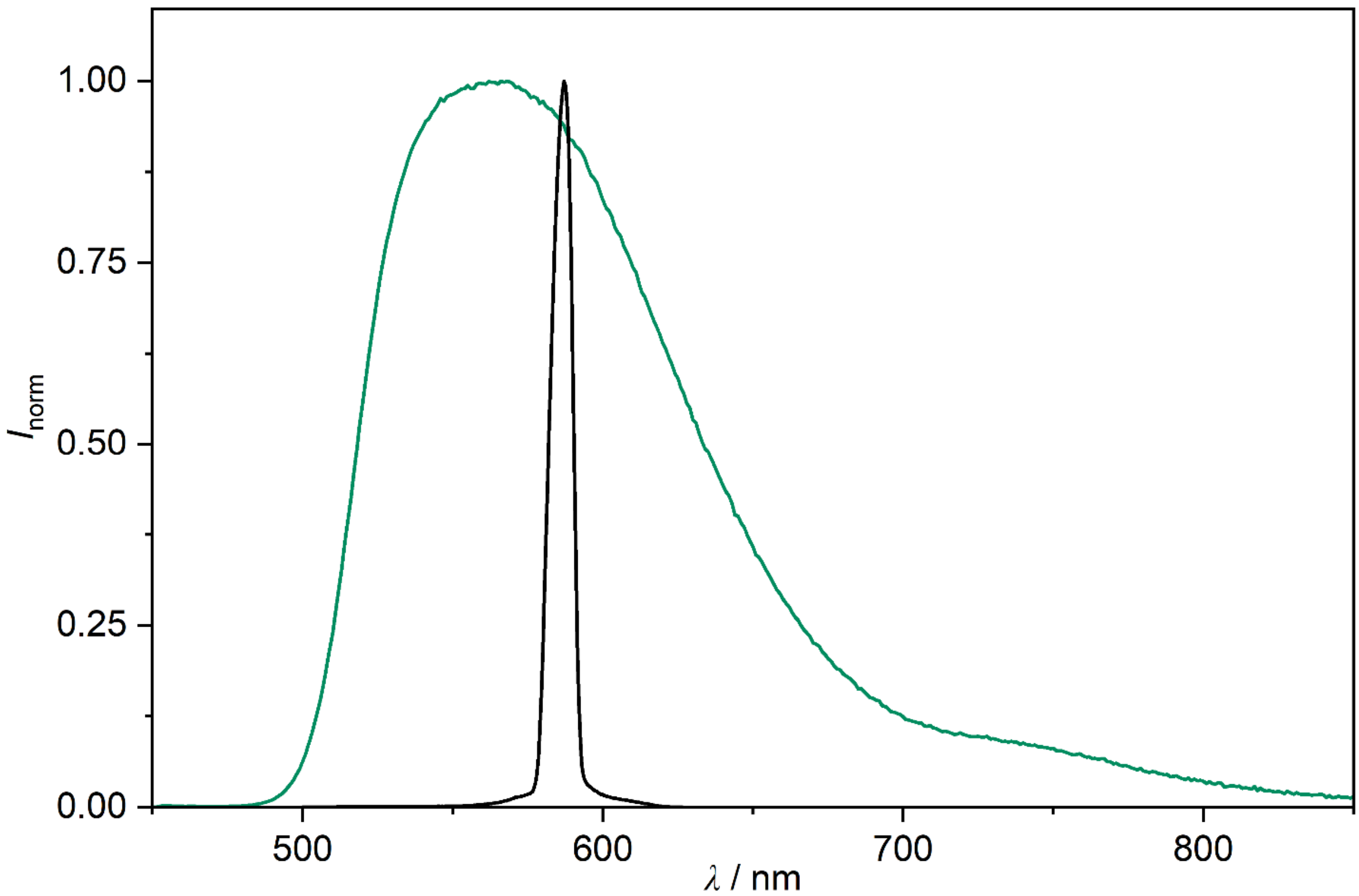

Supplementary Figure 15. Normalized emission spectra of the UHP-LED (λ_{exc} = 560 nm, green) and Mountain Photonics Hyperchromator (λ_{exc} = 590 nm, black) light sources employed for irradiation experiments.

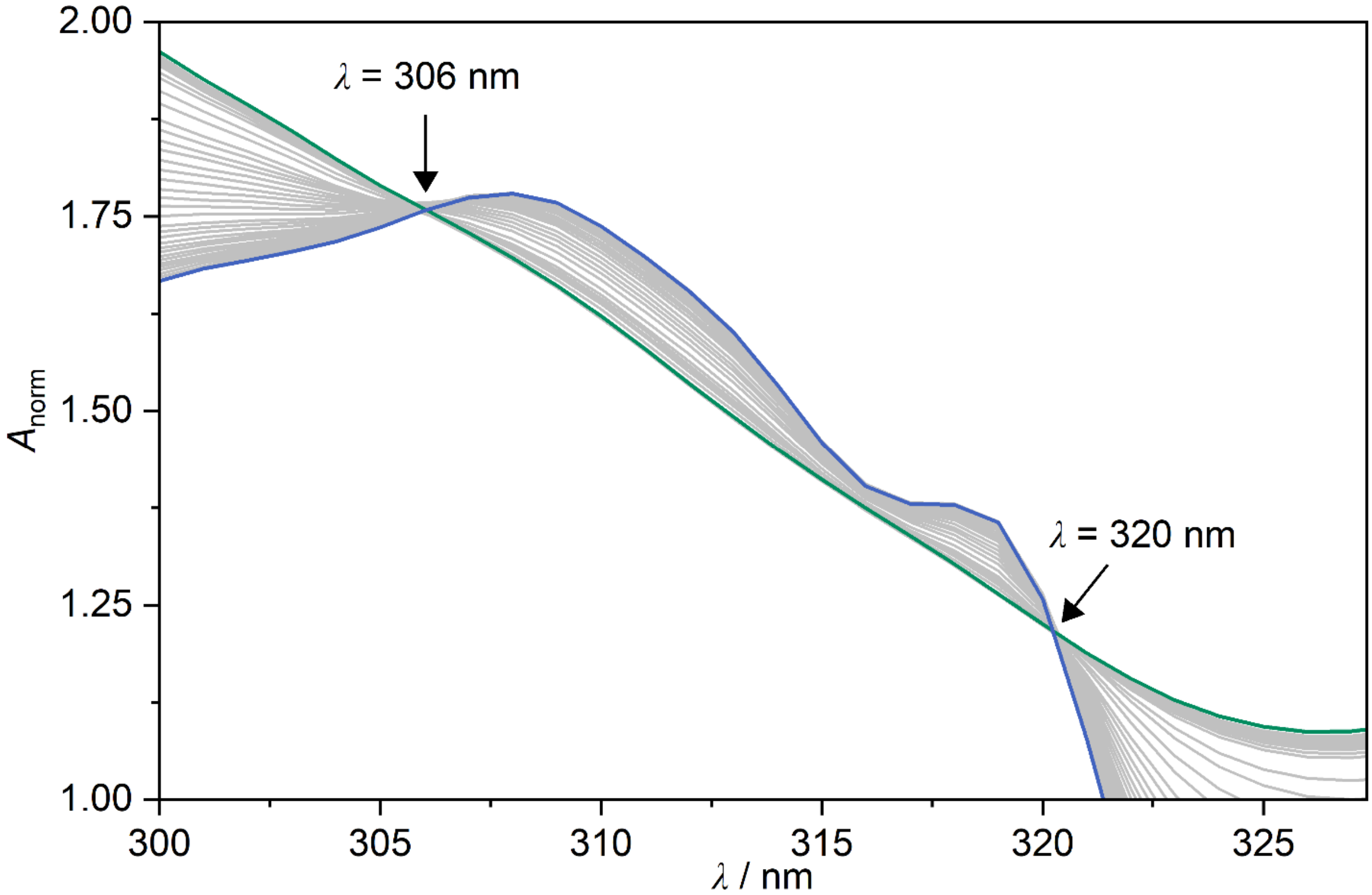

Supplementary Figure 16. Excerpt of the spectra of Fig. 3A displaying isosbestic points at $\lambda = 306$ nm and $\lambda = 320$ nm at 130 K after **LS-1** was irradiated at $\lambda_{exc} = 560$ nm for two minutes leading to **HS-1** formation (blue), which then converts to **LS-1** (green).

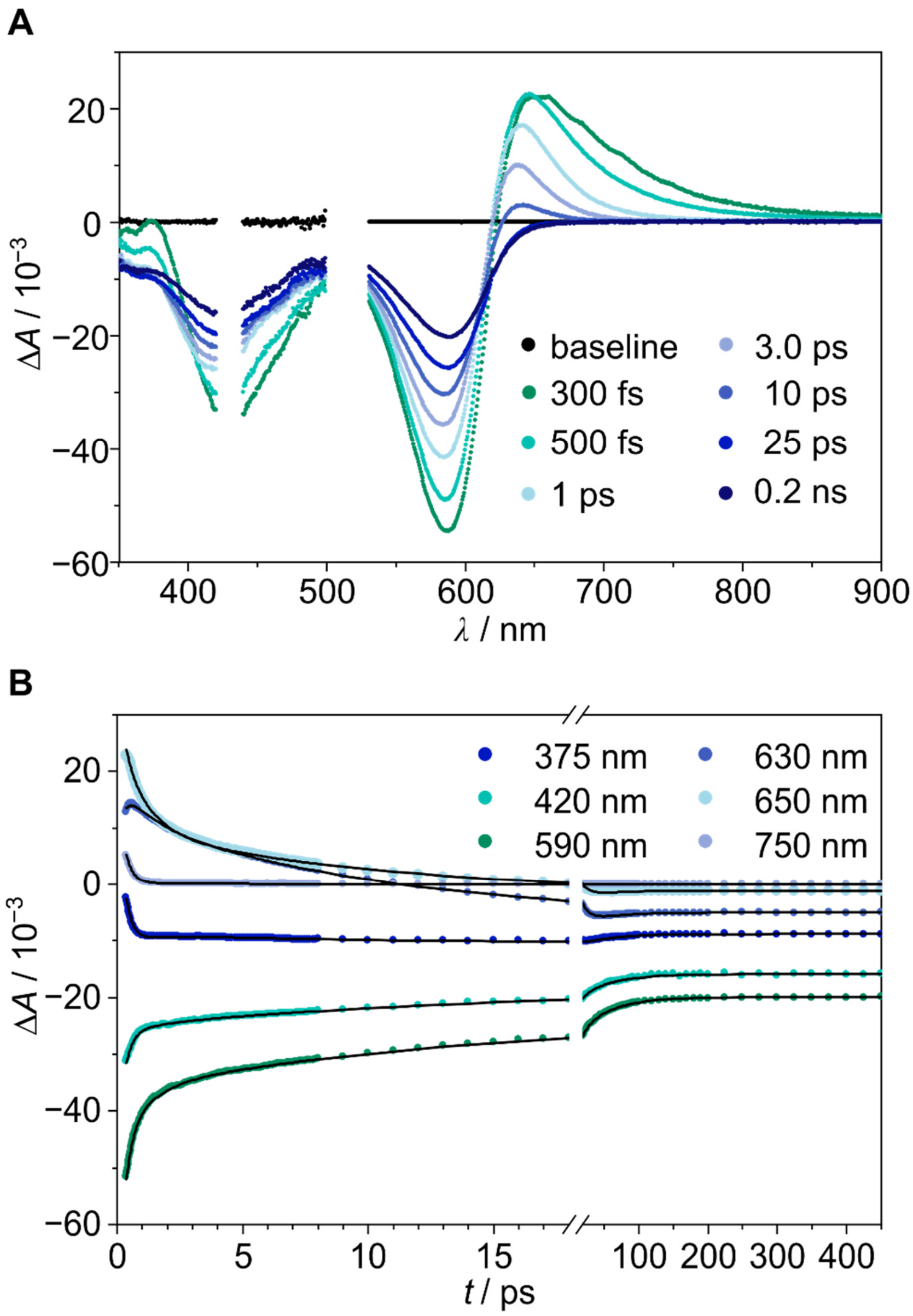

Supplementary Figure 17. fs-TA spectra of **1** in MeCN at 293 K after excitation at λ_{exc} = 430 nm (**A**) and the respective decay traces (**B**) (colored dots) for several wavelengths as well as fit curves (black lines) with R^2 = 0.9998.

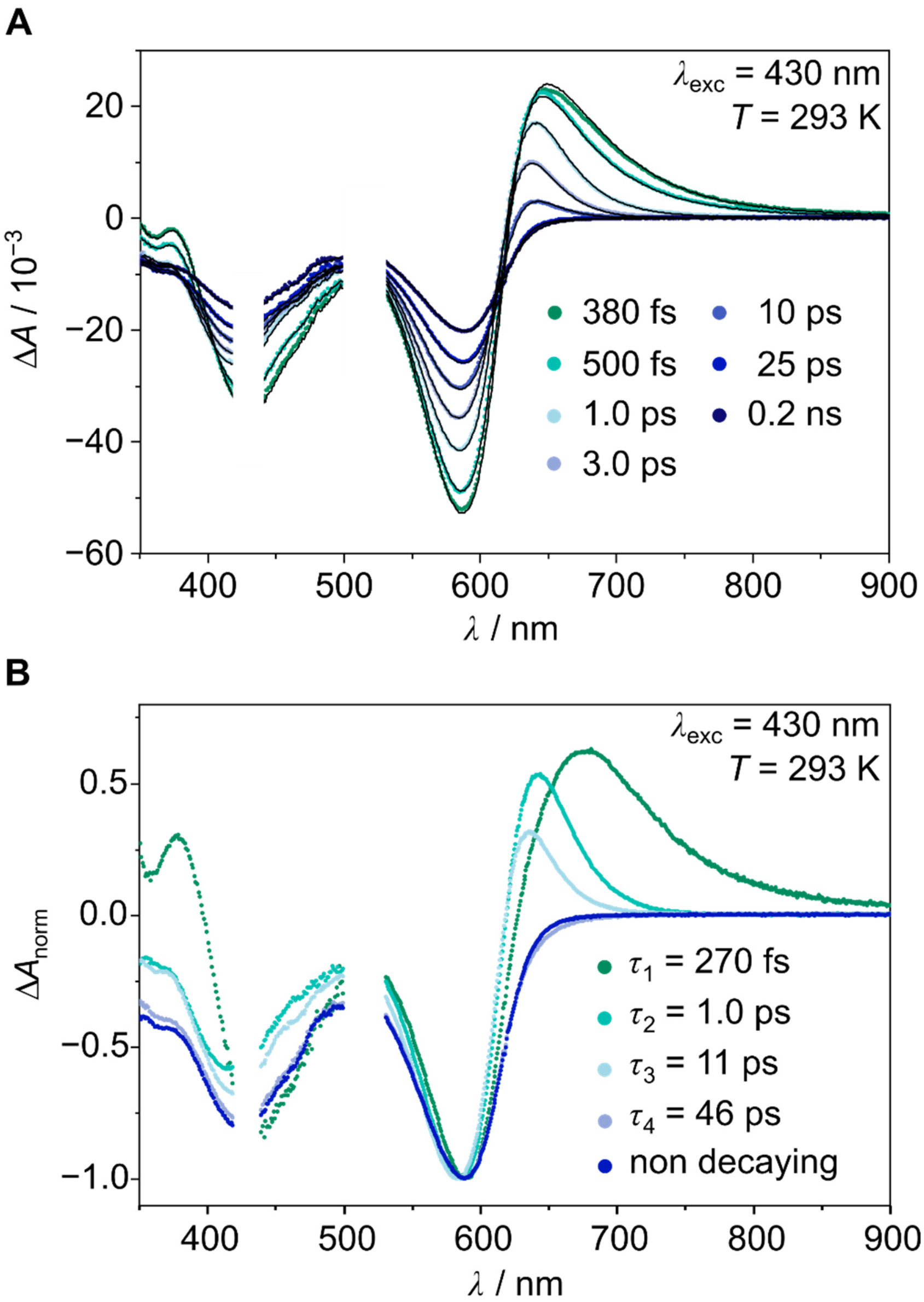

Supplementary Figure 18. Rebinned data (colored dots) vs fits (black lines, R^2 = 0.9998) from the fs-TA spectra (Fig. 17) of **1** in MeCN at 293 K after excitation at λ_{exc} = 430 nm (**A**). Respective evolution associated difference spectra (EADS) after global analysis (**B**).

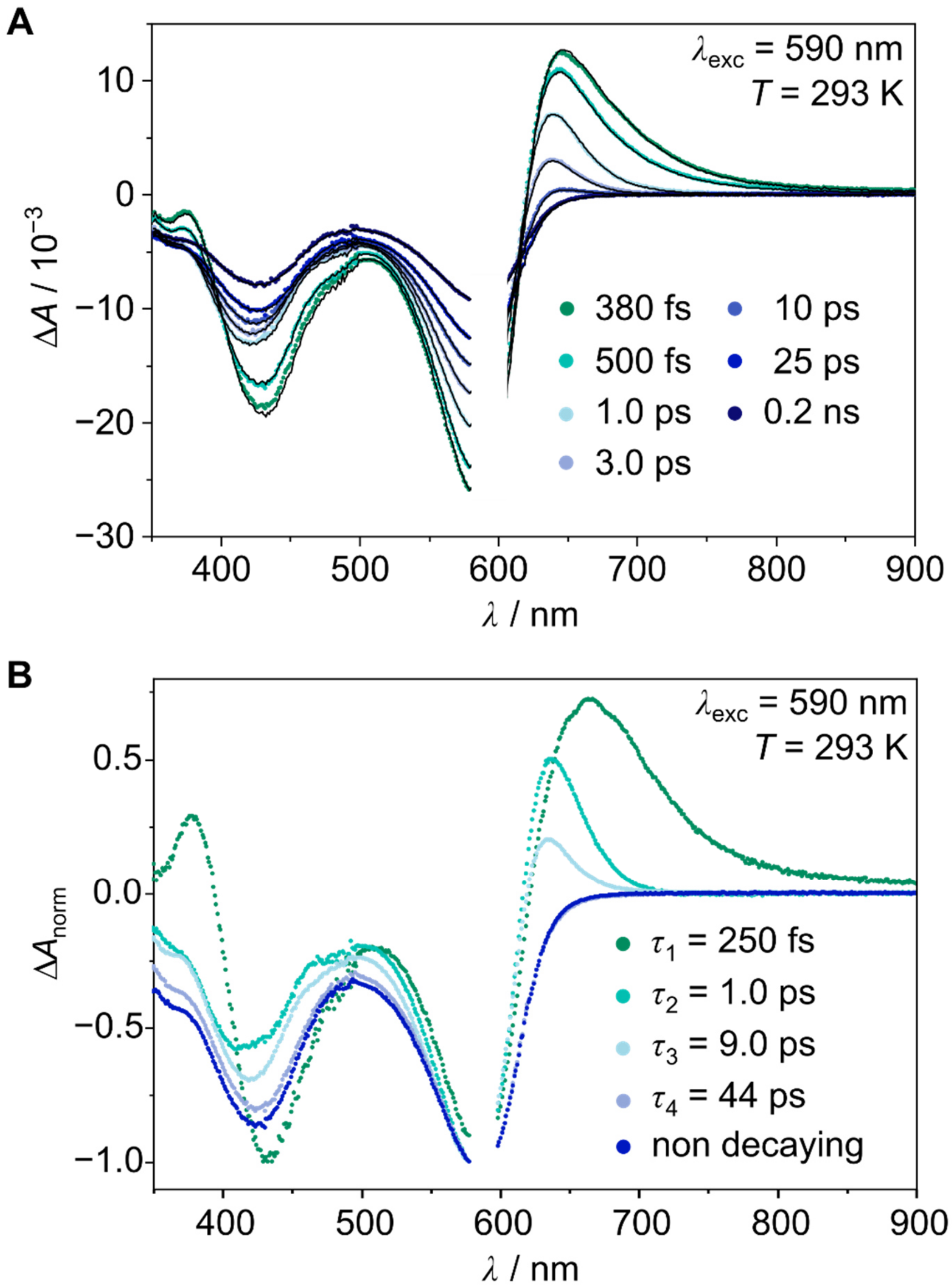

Supplementary Figure 19. Rebinned data (colored dots) vs fits (black lines, R^2 = 0.9997) from the fs-TA spectra (Fig. 3B) of **1** in MeCN at 293 K after excitation at λ_{exc} = 590 nm (**A**). Respective evolution associated difference spectra (EADS) after global analysis (**B**).

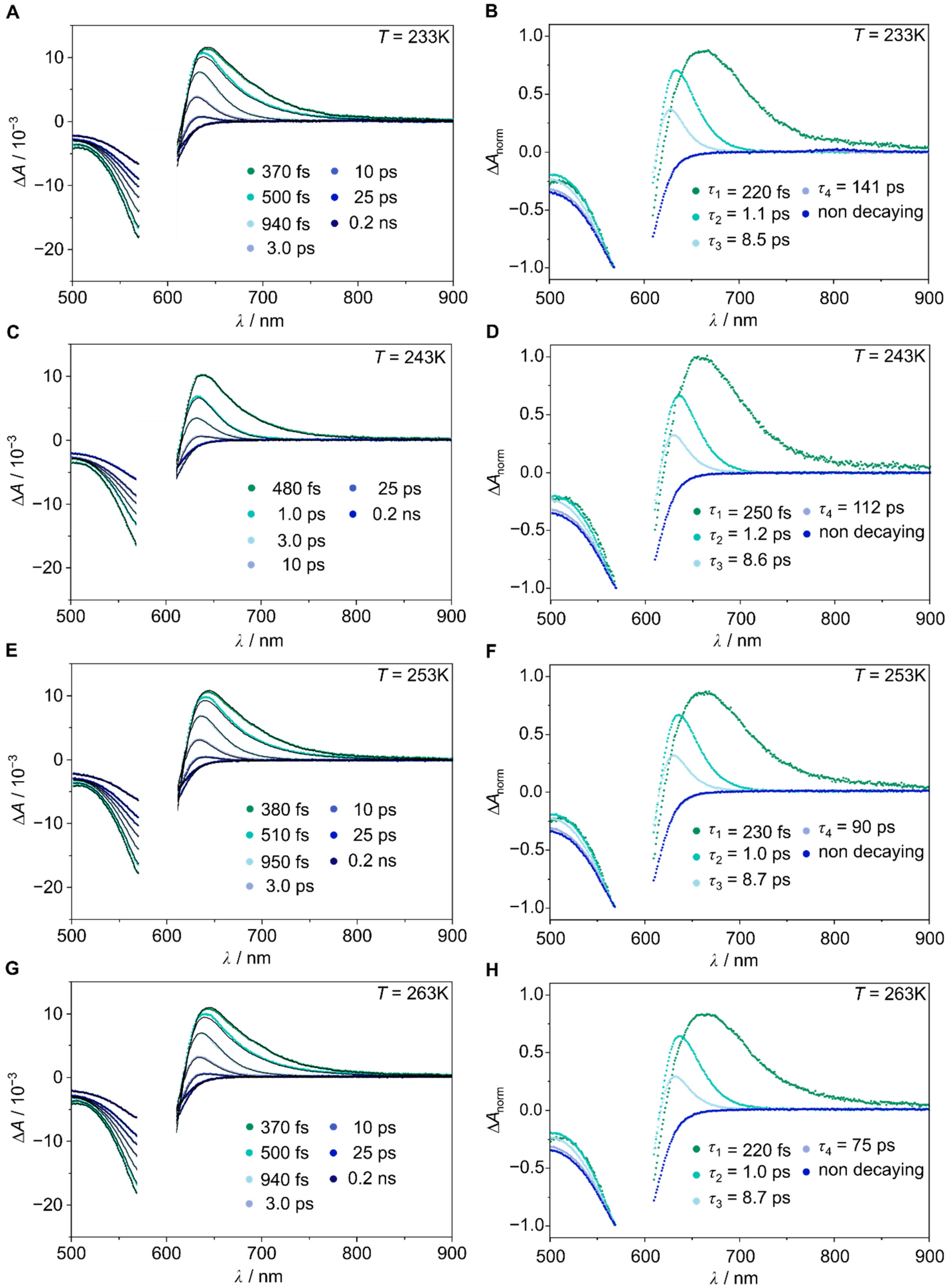
A
T = 233K
370 fs
500 fs
940 fs
3.0 ps
10 ps
25 ps
0.2 ns
ΔA / 10⁻³
λ / nm
B
T = 233K
τ1 = 220 fs
τ2 = 1.1 ps
τ3 = 8.5 ps
τ4 = 141 ps
non decaying
ΔAnorm
λ / nm
C
T = 243K
480 fs
1.0 ps
3.0 ps
10 ps
25 ps
0.2 ns
ΔA / 10⁻³
λ / nm
D
T = 243K
τ1 = 250 fs
τ2 = 1.2 ps
τ3 = 8.6 ps
τ4 = 112 ps
non decaying
ΔAnorm
λ / nm
E
T = 253K
380 fs
510 fs
950 fs
3.0 ps
10 ps
25 ps
0.2 ns
ΔA / 10⁻³
λ / nm
F
T = 253K
τ1 = 230 fs
τ2 = 1.0 ps
τ3 = 8.7 ps
τ4 = 90 ps
non decaying
ΔAnorm
λ / nm
G
T = 263K
370 fs
500 fs
940 fs
3.0 ps
10 ps
25 ps
0.2 ns
ΔA / 10⁻³
λ / nm
H
T = 263K
τ1 = 220 fs
τ2 = 1.0 ps
τ3 = 8.7 ps
τ4 = 75 ps
non decaying
ΔAnorm
λ / nm

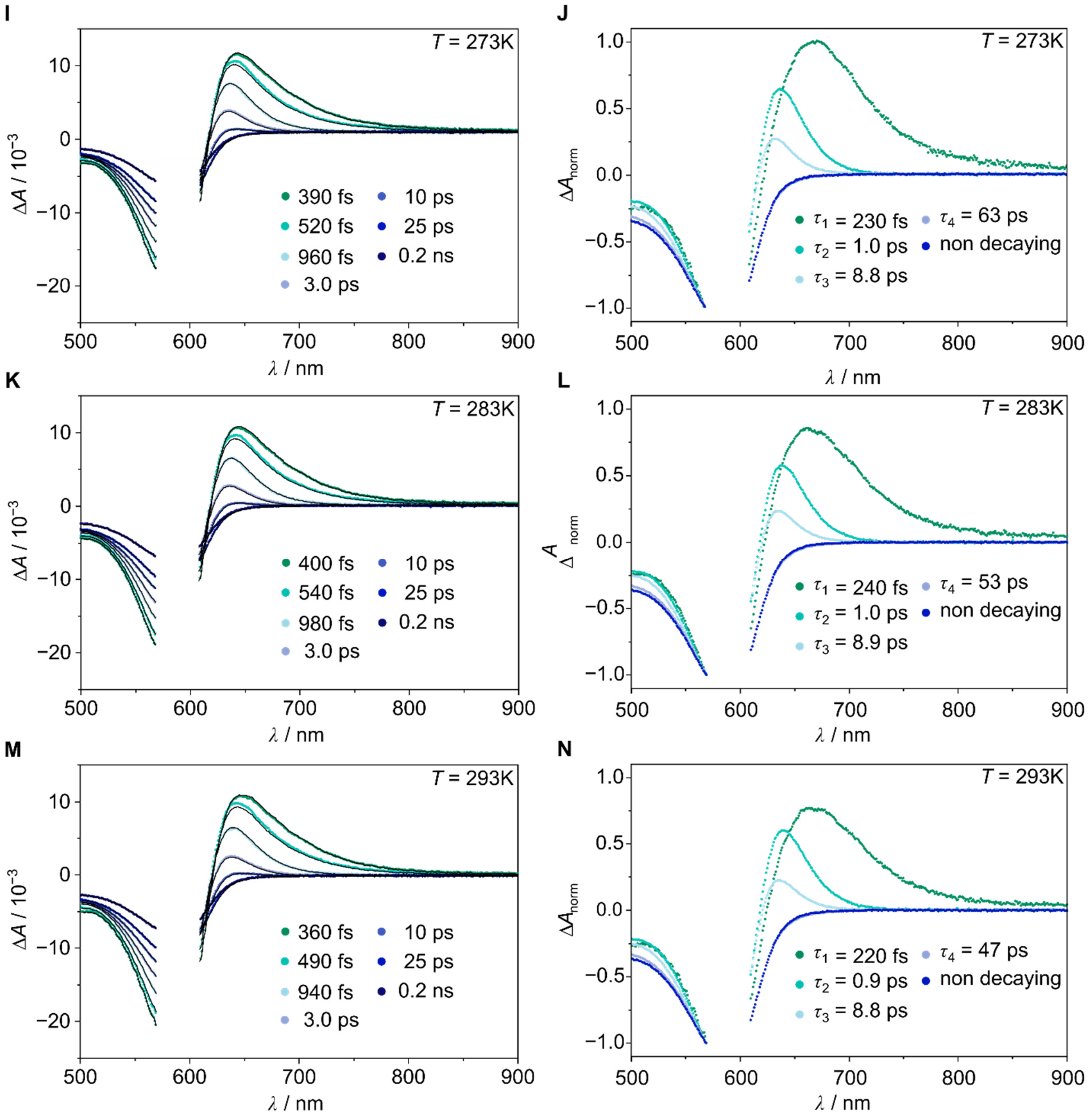

Supplementary Figure 20. Rebinned data (colored dots) vs fits (black lines) from the fs-TA spectra of **1** in MeCN after excitation at λ_{exc} = 590 nm at different temperatures T = 233 K (**A**, R^2 = 0.9994), T = 243 K (**C**, R^2 = 0.9995), T = 253 K (**E**, R^2 = 0.9995), T = 263 K (**G**, R^2 = 0.9996), T = 273 K (**I**, R^2 = 0.9996), T = 283 K (**K**, R^2 = 0.9996) and T = 293 K (**M**, R^2 = 0.9996). Respective evolution associated difference spectra (EADS) after global analysis at temperatures T = 233 K (**B**), T = 243 K (**D**), T = 253 K (**F**), T = 263 K (**H**), T = 273 K (**J**), T = 283 K (**L**) and T = 293 K (**N**), respectively.

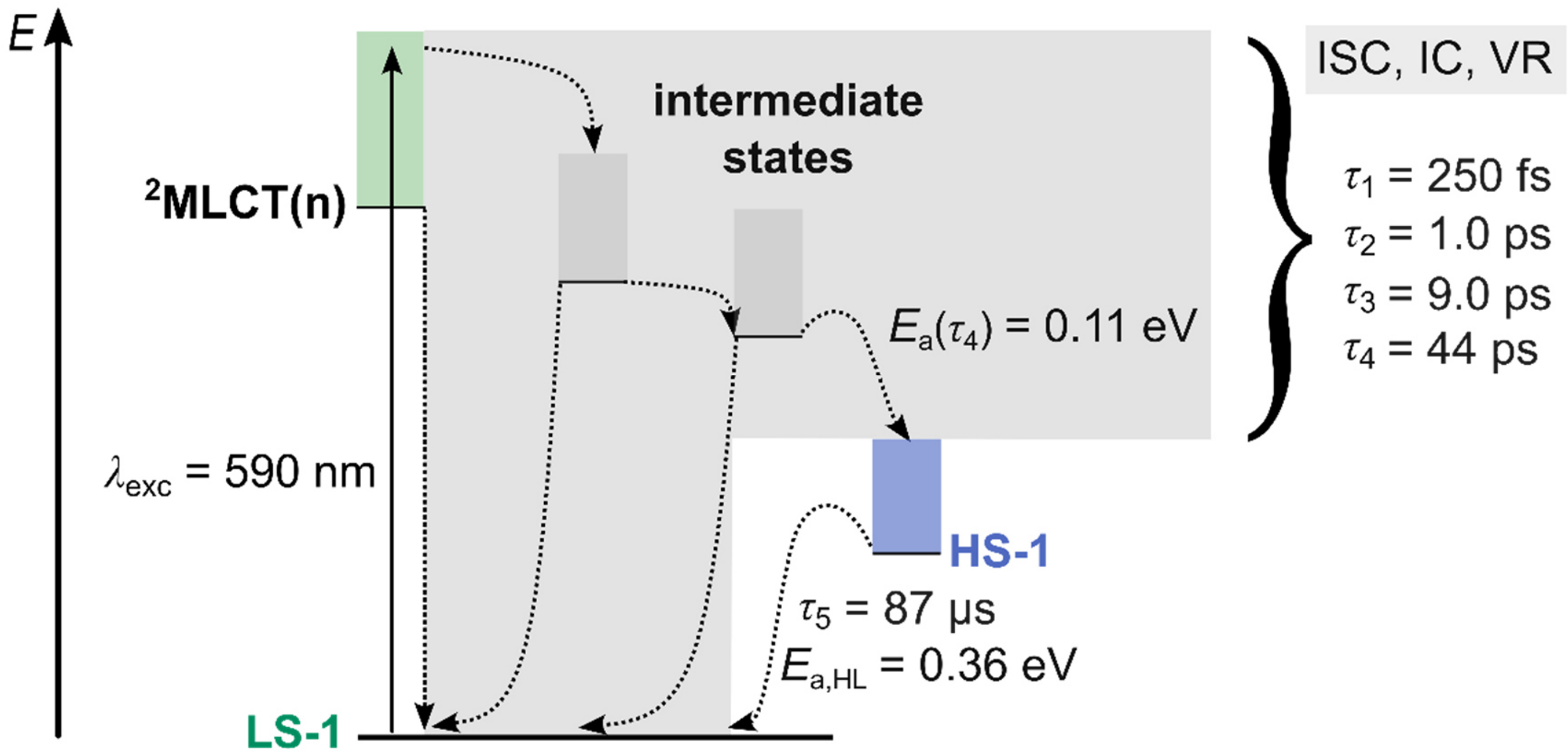

Supplementary Figure 21. Schematic energy diagram of **1**. Excitation with λ_{exc} = 590 nm light at T = 293 K leads to a spin-allowed electronic transition from **LS-1** to ^{2}MLCT(n) states. These can either relax non-radiatively via vibrational relaxation (VR) back to **LS-1** or can undergo internal conversion (IC) or intersystem crossing (ISC) to respective intermediate states. Again, non-radiative decay back to **LS-1** can occur also from these intemediate states. The component τ_4 = 44 ps is associated with a process that possesses a barrier of $\Delta E_a(\tau_4)$ = 0.11 eV leading to the **HS-1** state. The maximum quantum yield for the **HS-1** formation is 33%, i.e. 67% losses occur. The **HS-1** state finally decays back to **LS-1** with τ_5 = 87 µs and an associated barrier of $\Delta E_a(\tau_5)$ = 0.36 eV.

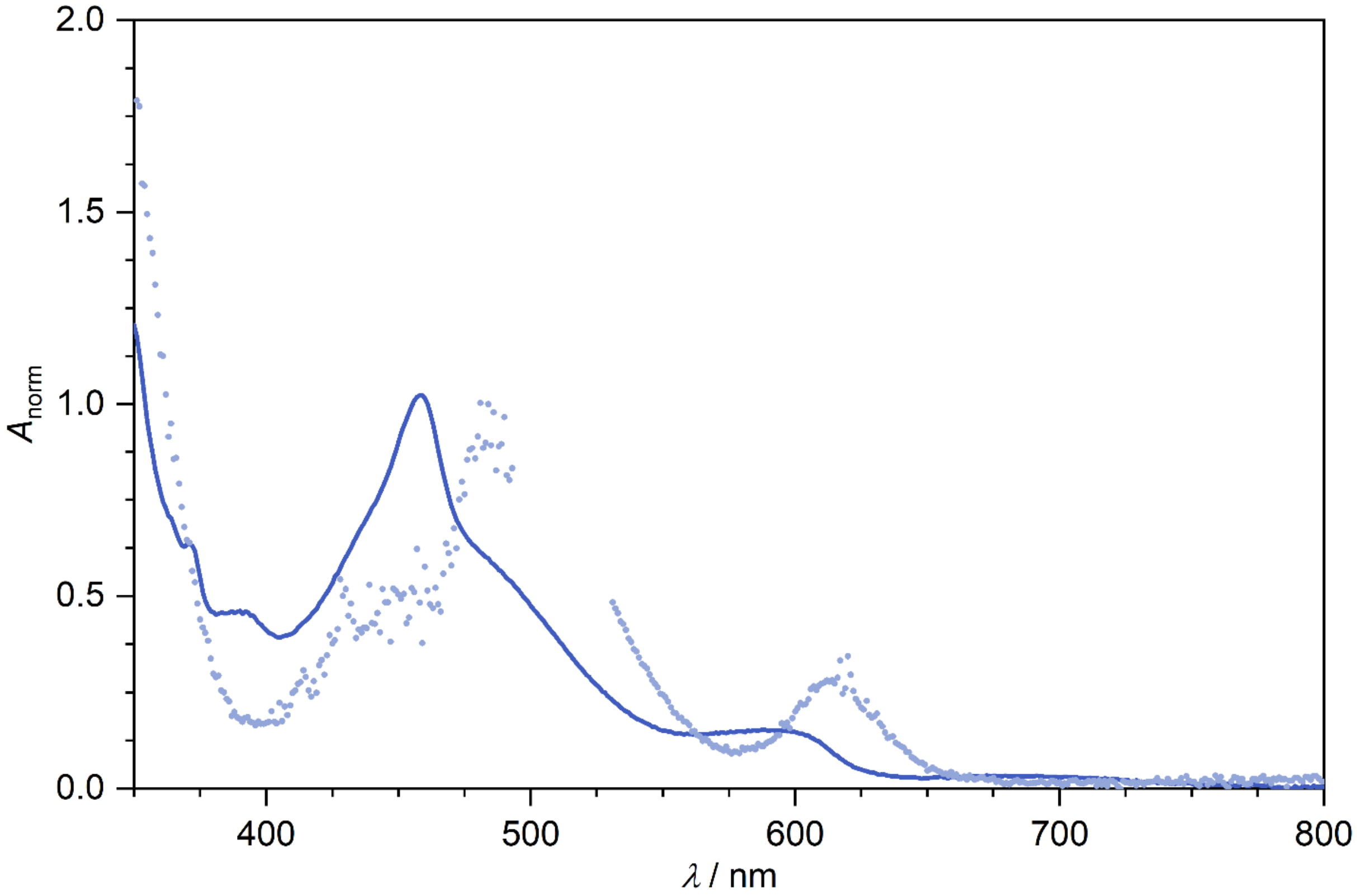

Supplementary Figure 22. UV-vis-NIR absorption spectrum of **HS-1** extracted from fs-TA spectra of **LS-1** in MeCN at λ_{exc} = 430 nm at 293 K by weighted addition of the ground state spectrum of **1** to the fs-TA spectrum (blue dots) and UV-vis-NIR absorption spectrum of **HS-1** obtained by irradiation of **LS-1** in 2-MeTHF/MeCN (29:1, *v*/*v*) at λ_{exc} = 560 nm at 95 K for 2 minutes (dark blue line) for comparison.

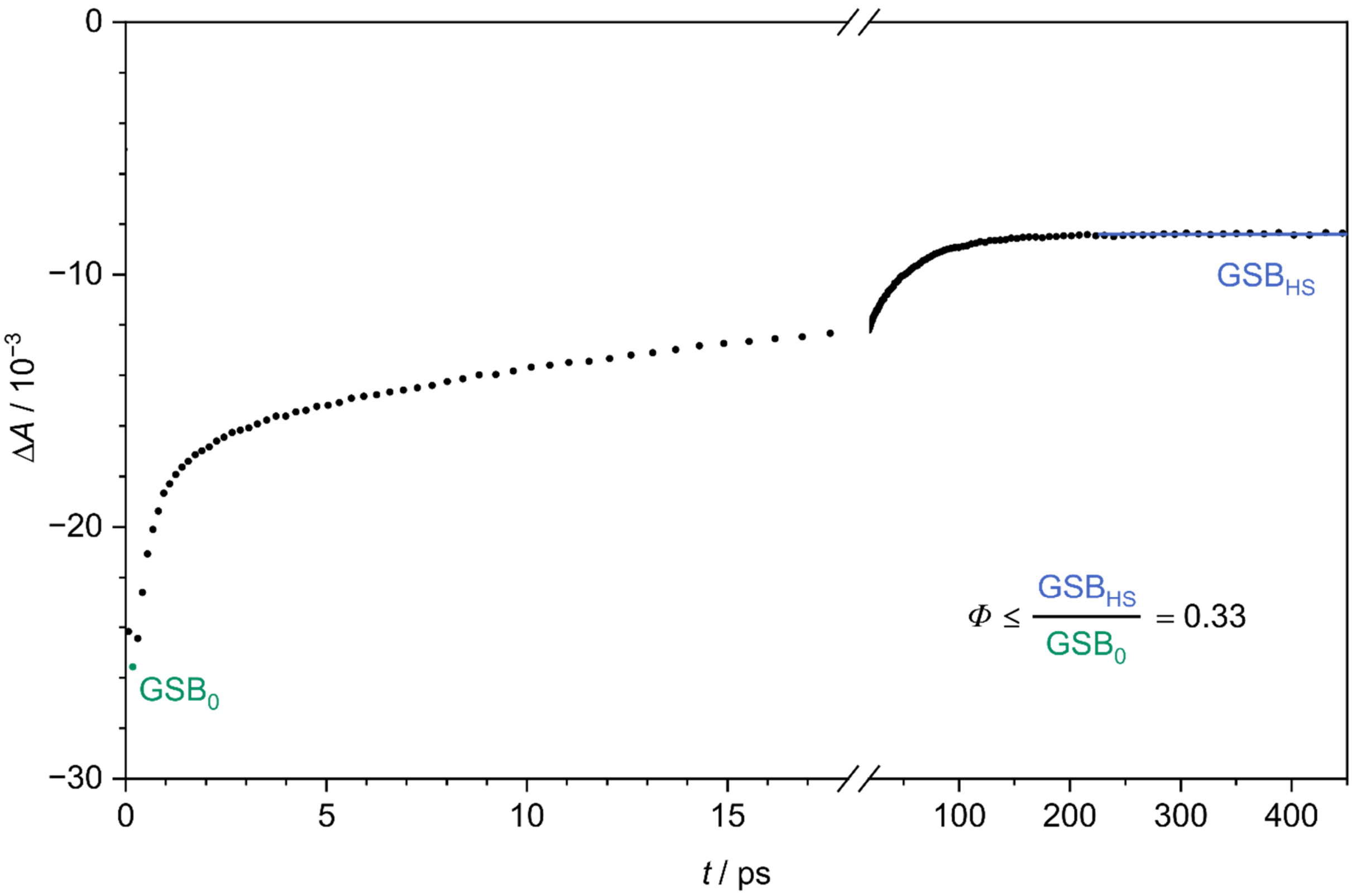

Supplementary Figure 23. Decay trace observed at λ = 570 nm to calculate the maximum **HS-1** quantum yield (Φ) upon excitation of **LS-1** at λ_{exc} = 590 nm in the TA spectroscopy setup.

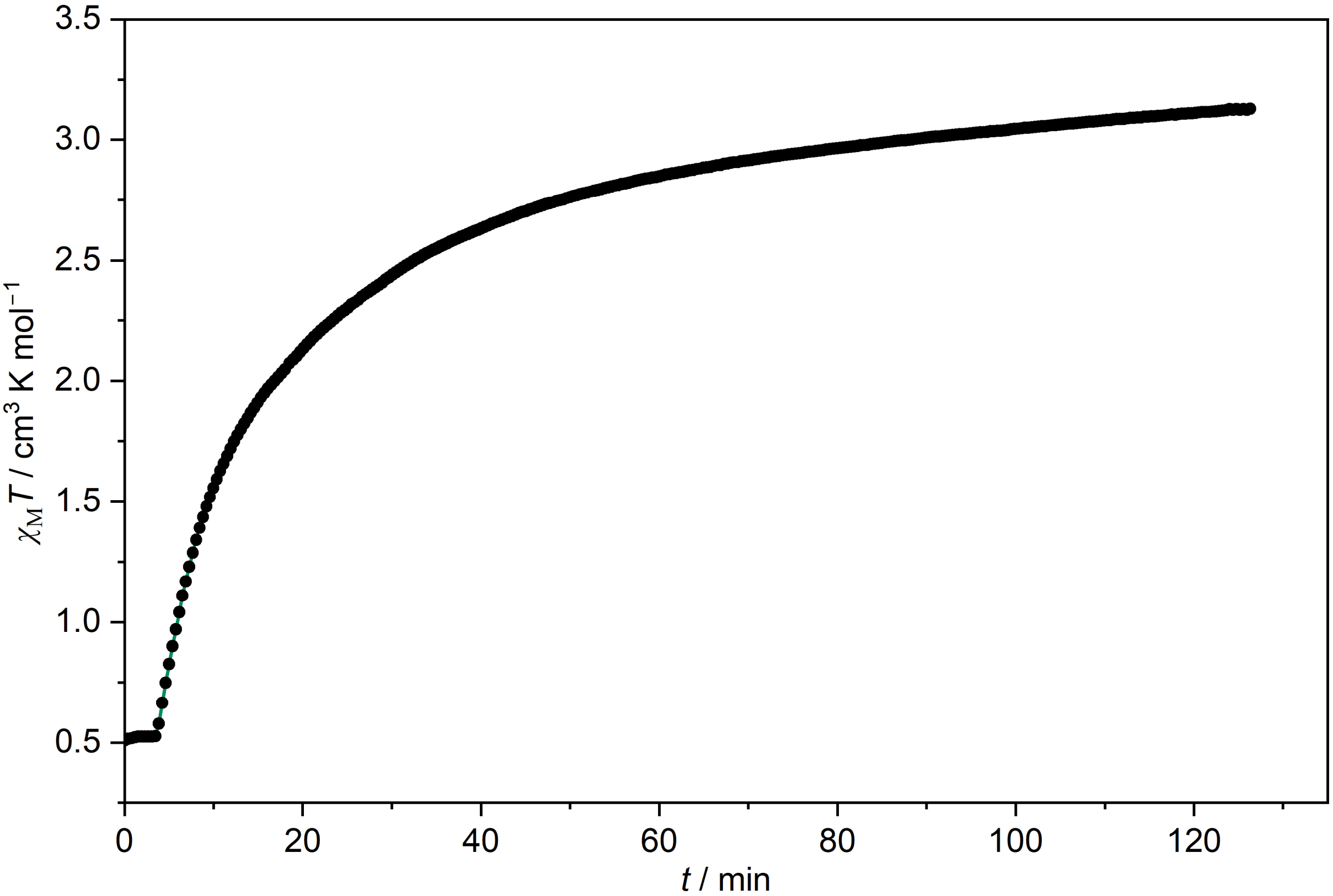

Supplementary Figure 24. Time-dependent profile of the product of the molar susceptibility and temperature $\chi_M T$ of **1** under irradiation at λ_{exc} = 590 nm at 10 K for the T_{LIESST} experiment in **Supplementary Figure 25**.

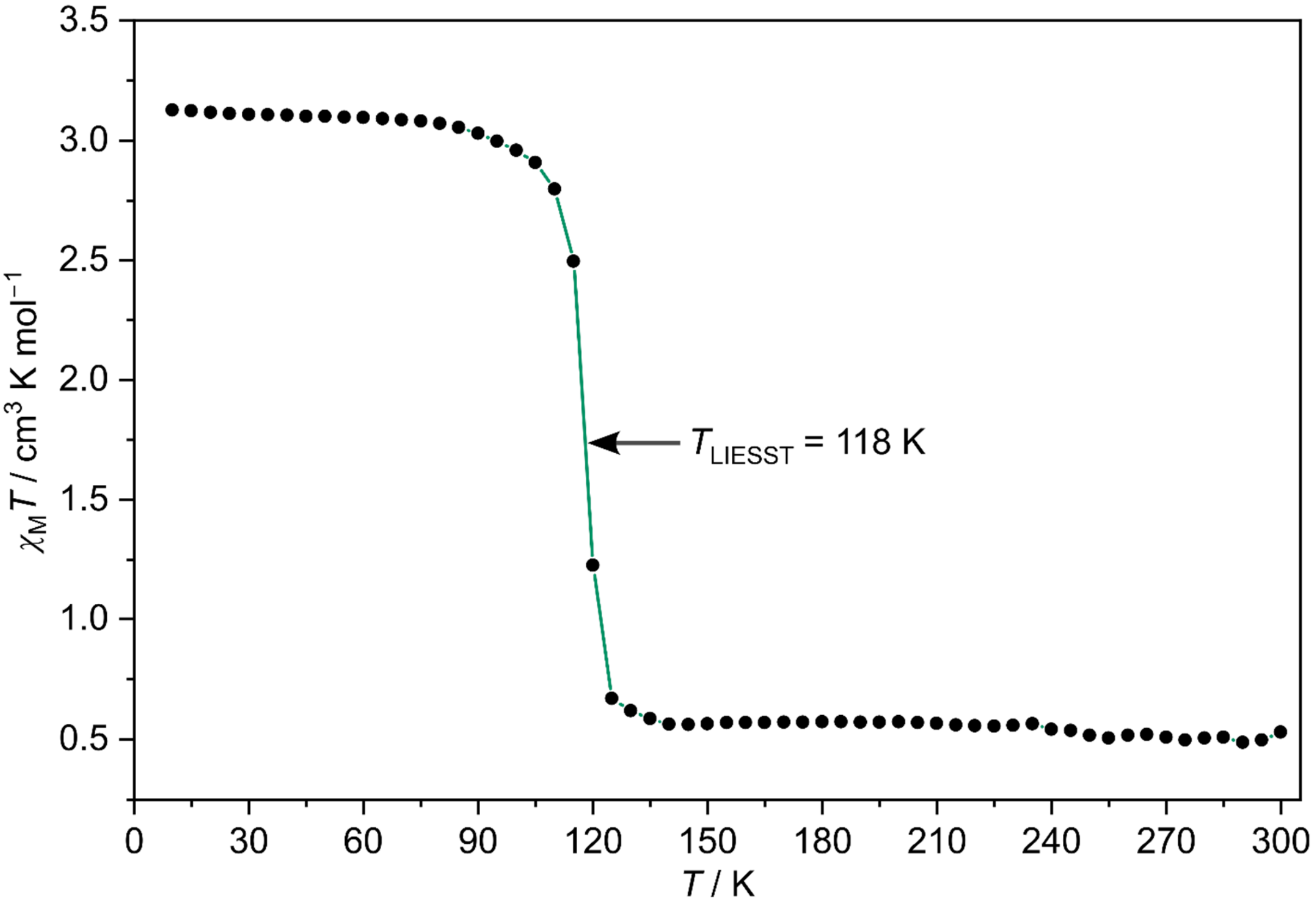

Supplementary Figure 25. T_{LIESST} determined for **1** after the sample was irradiated (Fig. 24) for two hours at λ_{exc} = 590 nm at 10 K, followed by temperature increase to 300 K with 2.5 K min^{-1} in the dark.

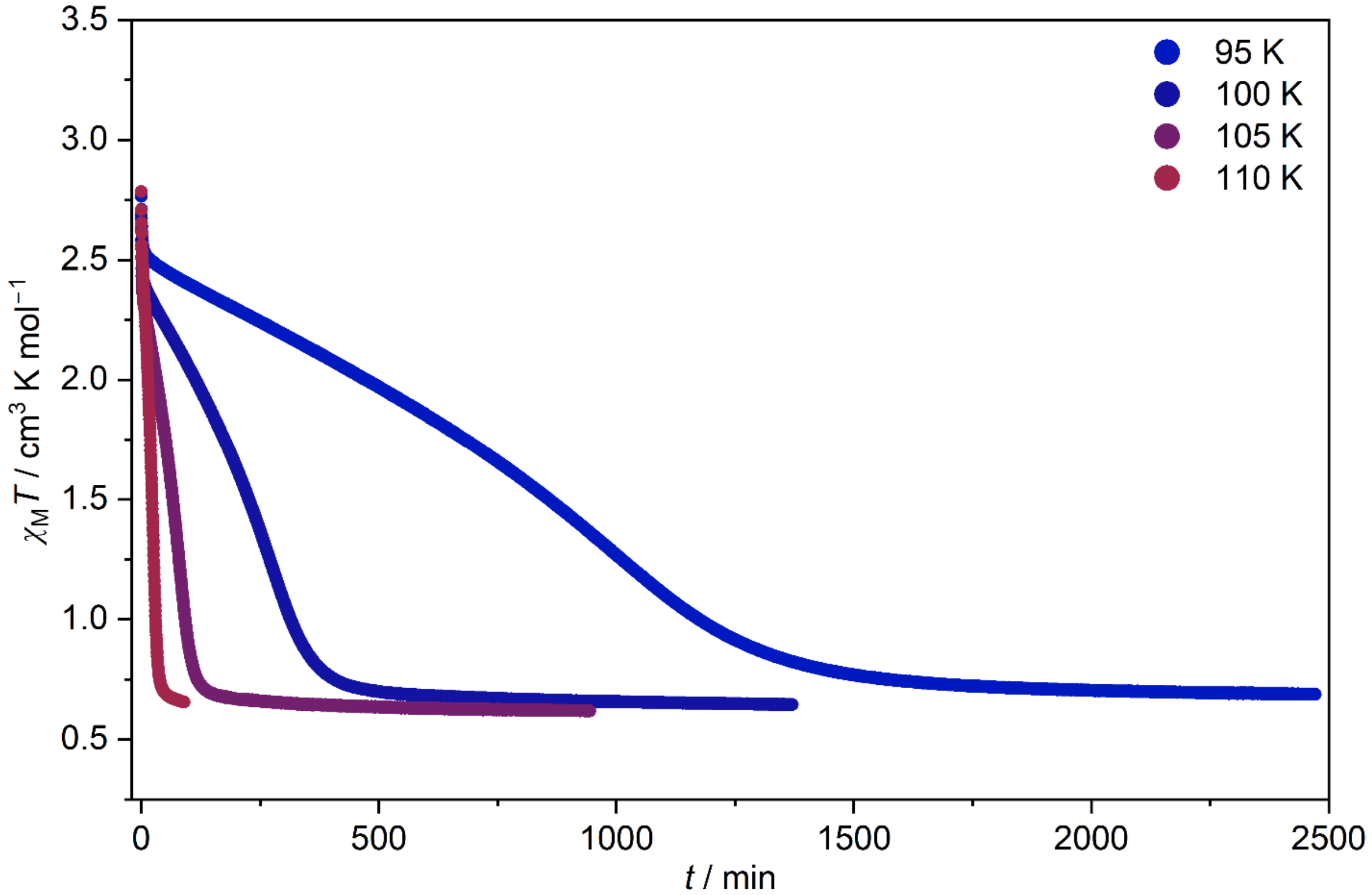

Supplementary Figure 26. HS → LS relaxation curves in the solid state as a function of temperature (95, 100, 105, 110 K) after irradiation of **1** for two hours at λ_{exc} = 590 nm at 10 K.

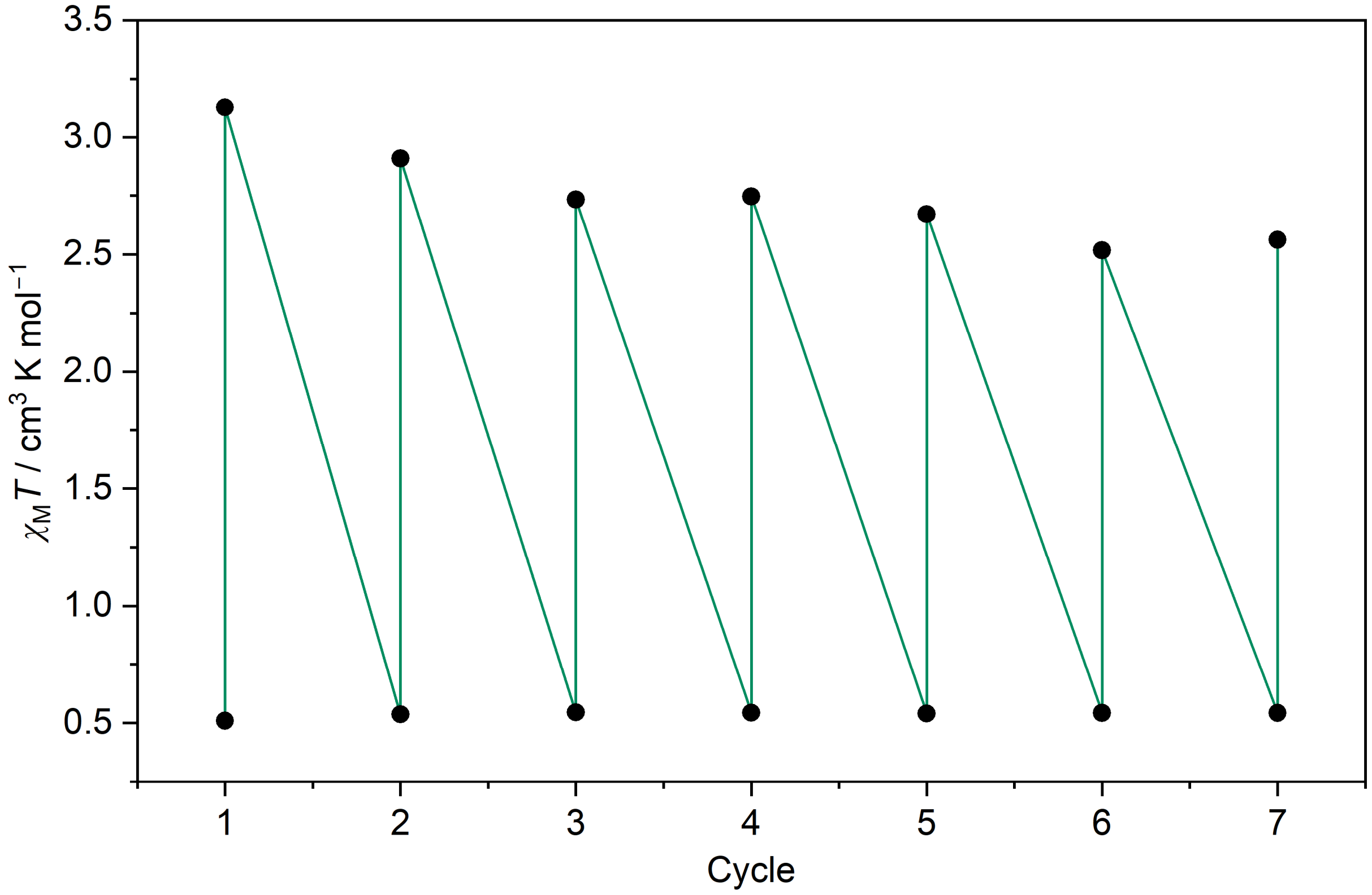

Supplementary Figure 27. $\chi_M T$ values measured at 10 K before and after irradiation of **1** at λ_{exc} = 590 nm for two hours. The temperature was increased to 300 K before starting a new cycle to ensure full recovery of **LS-1**.

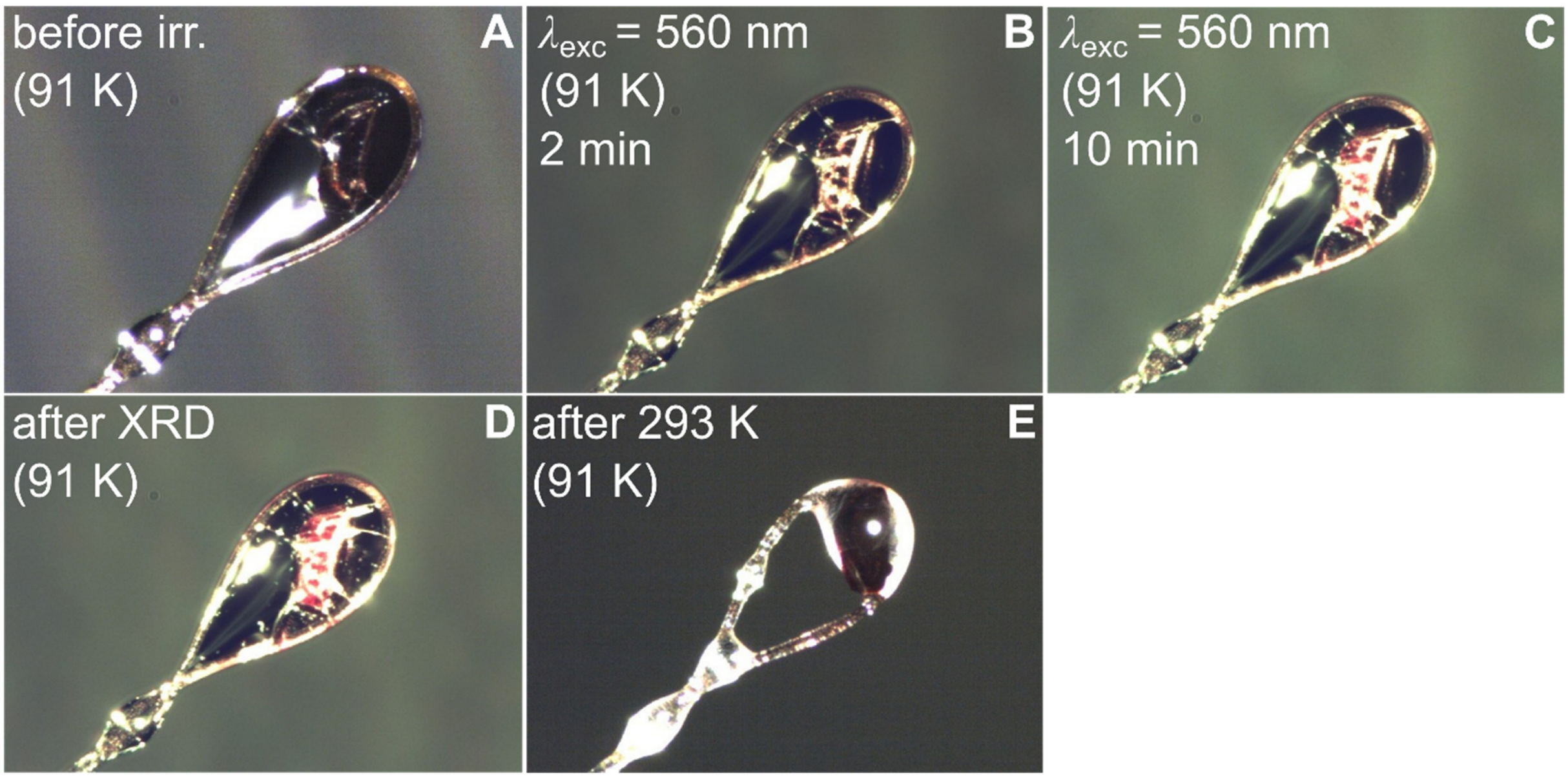

Supplementary Figure 28. Photographs of a single crystal of **1** before the XRD experiment (**A**), after irradiation at λ_{exc} = 560 nm for 2 minutes (**B**) and after 10 minutes (**C**) at 91 K. After the XRD experiment, the light source was switched off and the photograph was taken immediately (**D**), showing the intact orange crystal at 91 K. Subsequent heating of the crystal to 293 K was followed by cooling to 91 K (**E**).
