# Supplementary material for: Covalency control of photomagnetic relaxation in a manganese(II) photoswitch
Source: Nat Chem. 2026 Jun 29;18(10):1729–36. doi: 10.1038/s41557-026-02193-8 (PMC13623647; doi:10.1038/s41557-026-02193-8)
Supplement: Supplementary file 2 [file 41557_2026_2193_MOESM2_ESM.pdf]

nature portfolio

Peer Review File

Covalency control of photomagnetic relaxation in a manganese(II) photoswitch

Corresponding Author: Professor Katja Heinze

This file contains all reviewer reports in order by version, followed by all author rebuttals in order by version.

Attachments originally included by the reviewers as part of their assessment can be found at the end of this file.

Version 0:

Reviewer comments:

Reviewer #1

(Comments for the Author)
In this manuscript Heinze reports the novel observation of long lived photoexcited switching between the spin doublet and spin sextet forms of a Mn(II) complex, including trapping the photoexcited spin sextet form, a first in the spin crossover field and a landmark outcome. The result emerged as part of a comprehensive photophysical investigation of a six-coordinate mononuclear Mn(II) complex with pairs of tridentate carbene ligands. The authors support the experimental findings with a clear theoretical proposal which informs their targeted design of a metal-ligand complex with capacity to shift the normal coordinate Q between low spin and high spin forms of the metal ion. This is well thought out, well explained and provides an excellent theoretical basis to account for the highly unusual photophysical properties.

This result develops well from recent photophysical studies on the low spin d6 Mn(I) analogue of the low spin d5 Mn(II) complex which is the subject of the current submission. Using the same donor set, Heinze et al. demonstrated the electronic versatility of Mn in this C4N6 donor set in their 2025 paper in Nature Communications (https://doi.org/10.1038/s41467-025-63225-4), demonstrating by cyclic voltammetry that four oxidation states of manganese (+I, +II, +III, +IV) were accessible with pairs of pbmi carbene ligands. They demonstrated that long lived phosphorescence (190 ns) from a transient metal-centred triplet state was achievable at room temperature in the low spin Mn(I) complex, thereby opening a route to sustainable photochemical applications with earth abundant metals. What is new in the current work is changing the metal centre from d6 Mn(I) to d5 Mn(II) and trapping the photoexcited metal-centred sextet state at accessible temperatures, a TLIESST of 112.5 K is reported and a change in spin multiplicity of 2. This is a paradigm shifting result for the field – to detect spin state trapping in manganese and at such a high temperature, including collection of structural data on the photo-induced HS electronic form. This, coupled with the expert insights into how the system was designed to achieve the outcome, make this the most interesting work on photo-induced spin state trapping to emerge since Hauser's initial report on LIESST in 1984.

I note that during the review process a work has been accepted in Dalton Trans (DOI: 10.1039/d5dt02990b) reporting emission properties and quantum yield of a structurally related complex. The authors briefly refer to the 2025 preprint of the current Heinze submission, ChemRxiv, 2025, DOI: 10.26434/chemrxiv-2025-lrnqm) when assigning emission bands of their structural analogue (pbmi methyl groups replaced with phenyl), thus indicating their awareness of the Mainz study. The Dalton Trans authors (Swierk, Arias-Rotondo) also had a preprint of their article on the ChemRxiv (https://chemrxiv.org/doi/10.26434/chemrxiv-2024-r2qq0) so there is obviously interest in this area from at least one other group. However, the submission by Heinze goes well beyond what is reported in Dalton Trans, firstly in terms of the outstanding outcome of the first example of spin state trapping in manganese, secondly, the fact that this is achieved at such high temperatures in the Mainz experiment and finally in establishing a firm and clear theoretical rationale for the experimental design and interpretation. As such the work currently under review constitutes observation of a new phenomenon with detailed theoretical explanation of why it is achievable in this structural framework.

In terms of the experimental and theoretical results, the work is at the highest level in terms of depth and breadth of measurements, the detail of the explanation, the quality of the data and presentation. The conclusions are robustly underpinned by clearly presented data which is expertly analysed. Overall this is work at the highest level in every way. Prior work is well cited, the introduction and abstract are exceptionally clear and take the reader on a journey through the literature to the current point and show clearly what is novel here.

Publication of this work is recommended and some suggestions below may be taken into account when preparing a revised manuscript.

1. Consider changing “manganese photoswitch” in title to “manganese(II) photoswitch”

2. Page 2 line 48 – swap manganese(II) and manganese(III) as the references are mis-assigned – refs 11,12 should be to Mn(II) complexes, refs 13,14 to Mn(III) complexes.

3. Page 2 – line 50 – light induced switching between LS and HS forms of Fe(II) was first reported by J. J. McGarvey in a transient experiment at room temperature in solution, J. Chem. Soc., Chem. Commun., 1982, 906-907, DOI https://doi.org/10.1039/C39820000906, before the first low temperature trapping experiment reported by Hauser et al in 1984, current ref 18. The authors may wish to refer to McGarvey’s transient experiment as they set the scene for their own work and define LIESST as opposed to fast processes here.

4. Page 2, line 65 – please provide a reference when citing the Ea,HL value of around 0.09eV for polypyridine iron(II) complexes.

5. Page 3 line 85 - please look again at the discussion on the relationship between T1/2 and T(LIESST) which has been a point of discussion in the community since Letard’s work in 2006 suggesting the opposite, i.e. that high T(LIESST) values require lower T1/2 values due to the inverse linear relationship between the two. Letard proposed that T(LIESST) = T(0) – 0.3T(1/2) in his article in J. Mater. Chem., 2006,16, 2550-2559, https://doi.org/10.1039/B603473J.

6. Page 4, line 98 – explain why higher Mn-C covalency should significantly destabilize the HS state.

Reviewer #2

(Comments for the Author)

Reviewer #3

(Comments for the Author)
review attached

Reviewer #4

(Comments for the Author)
1. The manuscript reports a Mn(II) carbene complex that exhibits LIESST with remarkably slow HS to LS relaxation kinetics. By increasing metal–ligand covalency, the authors propose a strategy to simultaneously enlarge the electronic energy gap and the structural reorganization energy, thereby increasing the activation barrier for relaxation.
2. The work is conceptually original in extending LIESST behavior to a Mn(II) carbene platform and in explicitly using metal–ligand covalency as a design parameter to modulate photomagnetic relaxation. Most prior LIESST studies have focused on Fe(II) or Fe(III) systems. Reports of Mn-based LIESST are rare, and the deliberate manipulation of structural reorganization energy via covalency represents a meaningful conceptual advance.
The proposed covalency control strategy may provide a new direction beyond traditional the activation barrier tuning approaches and is therefore significant for the photo-switching.
3. The approach is appropriate and generally well executed.
The magnetic and spectroscopic data appear of high quality. Structural changes between spin states are clearly supported by both experiment and computation.
Figures are generally clear and logically organized. The conceptual figure is particularly effective.
Pls check following points.
The Arrhenius equation and LIESST misspelling.
4. While activation energies and rate constants are reported, uncertainties are not systematically propagated in the comparison between solution and solid-state barriers and fitted Arrhenius parameters.
5. The qualitative conclusions, namely that increased covalency increases structural reorganization energy and slows relaxation, are robust and well supported by structural and spectroscopic evidence.
6. Authors should re-consider following.
6-1. Arrhenius fits including confidence intervals for extrapolated lifetimes.
6-2. Additional relaxation data at lower temperatures.
6-3. Residual plots or goodness-of-fit metrics for kinetic modeling.
6-4. A clearer kinetic scheme integrating ultrafast time constants.
7. The manuscript appropriately references foundational LIESST and SCO literature.
8. The manuscript reports a conceptually important and likely publishable advance. However, because the central quantitative claim relies on extrapolation and lacks full statistical treatment, additional quantitative rigor is required before acceptance in NC. With these revisions, the work would represent a strong and influential contribution to photo-switching materials.

Version 1:

Reviewer comments:

Reviewer #1

(Comments for the Author)
The authors have addressed all points raised in my original review very satisfactorily and it is clear they have adapted the majority of the suggestions of the other reviewers. This is a very novel work and I hope it will soon be in the public peer-reviewed domain.

Reviewer #2

(Comments for the Author)
The crystallography results have been improved and are suitable for publication

Reviewer #3

(Comments for the Author)
review attached

Reviewer #4

(Comments for the Author)
Summary of the key results
The manuscript reports a manganese(II) photoswitch exhibiting LIESST with exceptionally long-lived high-spin states enabled by enhanced metal–ligand covalency.

Originality and significance
Highly original and significant, extending LIESST to Mn(II) systems and introducing a new covalency-based design concept.

Data & methodology
Methods and data are appropriate, comprehensive, and of high quality.

Statistics and uncertainties
Data analysis and treatment of uncertainties are appropriate.

Conclusions
Well supported and robust.

Suggested improvements
All reviewer comments have been satisfactorily addressed. No further revision is required.

References
Appropriate.

Clarity and context
Clear and well structured.

Overall recommendation
I recommend acceptance in the present form without revision because authors have revised based on reviewers' comments.

Open Access This Peer Review File is licensed under a Creative Commons Attribution 4.0 International License, which permits use, sharing, adaptation, distribution and reproduction in any medium or format, as long as you give appropriate credit to the original author(s) and the source, provide a link to the Creative Commons license, and indicate if changes were made.
In cases where reviewers are anonymous, credit should be given to 'Anonymous Referee' and the source.
The images or other third party material in this Peer Review File are included in the article's Creative Commons license, unless indicated otherwise in a credit line to the material. If material is not included in the article's Creative Commons license and your intended use is not permitted by statutory regulation or exceeds the permitted use, you will need to obtain permission directly from the copyright holder.
To view a copy of this license, visit https://creativecommons.org/licenses/by/4.0/

Reviewer #1 (Comments for the Author):

In this manuscript Heinze reports the novel observation of long lived photoexcited switching between the spin doublet and spin sextet forms of a Mn(II) complex, including trapping the photoexcited spin sextet form, a first in the spin crossover field and a landmark outcome. The result emerged as part of a comprehensive photophysical investigation of a six-coordinate mononuclear Mn(II) complex with pairs of tridentate carbene ligands. The authors support the experimental findings with a clear theoretical proposal which informs their targeted design of a metal-ligand complex with capacity to shift the normal coordinate Q between low spin and high spin forms of the metal ion. This is well thought out, well explained and provides an excellent theoretical basis to account for the highly unusual photophysical properties.

This result develops well from recent photophysical studies on the low spin d6 Mn(I) analogue of the low spin d5 Mn(II) complex which is the subject of the current submission. Using the same donor set, Heinze et al. demonstrated the electronic versatility of Mn in this C4N6 donor set in their 2025 paper in Nature Communications (https://doi.org/10.1038/s41467-025-63225-4), demonstrating by cyclic voltammetry that four oxidation states of manganese (+I, +II, +III, +IV) were accessible with pairs of pbmi carbene ligands. They demonstrated that long lived phosphorescence (190 ns) from a transient metal-centred triplet state was achievable at room temperature in the low spin Mn(I) complex, thereby opening a route to sustainable photochemical applications with earth abundant metals. What is new in the current work is changing the metal centre from d6 Mn(I) to d5 Mn(II) and trapping the photoexcited metal-centred sextet state at accessible temperatures, a TLIESST of 112.5 K is reported and a change in spin multiplicity of 2. This is a paradigm shifting result for the field – to detect spin state trapping in manganese and at such a high temperature, including collection of structural data on the photo-induced HS electronic form. This, coupled with the expert insights into how the system was designed to achieve the outcome, make this the most interesting work on photo-induced spin state trapping to emerge since Hauser's initial report on LIESST in 1984.

I note that during the review process a work has been accepted in Dalton Trans (DOI: 10.1039/d5dt02990b) reporting emission properties and quantum yield of a structurally related complex. The authors briefly refer to the 2025 preprint of the current Heinze submission, ChemRxiv, 2025, DOI: 10.26434/chemrxiv-2025-lrnqm) when assigning emission bands of their structural analogue (pbmi methyl groups replaced with phenyl), thus indicating their awareness of the Mainz study. The Dalton Trans authors (Swierk, Arias-Rotondo) also had a preprint of their article on the ChemRxiv (https://chemrxiv.org/doi/10.26434/chemrxiv-2024-r2qq0) so there is obviously interest in this area from at least one other group. However, the submission by Heinze goes well beyond what is reported in Dalton Trans, firstly in terms of the outstanding outcome of the first example of spin state trapping in manganese, secondly, the fact that this is achieved at such high temperatures in the Mainz experiment and finally in establishing a firm and clear theoretical rationale for the experimental design and interpretation. As such the work currently under review constitutes observation of a new phenomenon with detailed theoretical explanation of why it is achievable in this structural framework. In terms of the experimental and theoretical results, the work is at the highest level in terms of depth and breadth of measurements, the detail of the explanation, the quality of the data and presentation. The conclusions are robustly underpinned by clearly presented data which is expertly analysed. Overall this is work at the highest level in every way. Prior work is well cited, the introduction and abstract are exceptionally clear and take the reader on a journey through the literature to the current point and show clearly what is novel here. Publication of this work is recommended and some suggestions below may be taken into account when preparing a revised manuscript.

We thank this reviewer for the very enthusiastic and encouraging assessment of our study.

1. Consider changing "manganese photoswitch" in title to "manganese(II) photoswitch"

Thank you for this suggestion. We have modified the title to include the oxidation state of the manganese(II) centre: "Covalency control of photomagnetic relaxation in a manganese(II) photoswitch"

2. Page 2 line 48 – swap manganese(II) and manganese(III) as the references are mis-assigned – refs 11,12 should be to Mn(II) complexes, refs 13,14 to Mn(III) complexes.

We thank the reviewer for spotting this mistake. We have corrected the manuscript accordingly: "manganese(II)[11,12], manganese(III)[13,14]"

3. Page 2 – line 50 – light induced switching between LS and HS forms of Fe(II) was first reported by J. J. McGarvey in a transient experiment at room temperature in solution, J. Chem. Soc., Chem. Commun., 1982, 906-907, DOI https://doi.org/10.1039/C39820000906, before the first low temperature trapping experiment reported by Hauser et al in 1984, current ref 18. The authors may wish to refer to McGarvey's transient experiment as they set the scene for their own work and define LIESST as opposed to fast processes here.

This is an excellent suggestion. We have included this in the manuscript as follows (new ref. 18): "This phenomenon was first observed in 1982, when laser excitation of LS iron(II) complexes transiently populates the HS state at room temperature.[18] Only two years later, the photogenerated HS state was trapped at cryogenic temperatures, establishing the phenomenon as light-induced excited spin state trapping (LIESST).[19]"

4. Page 2, line 65 – please provide a reference when citing the Ea,HL value of around 0.09eV for polypyridine iron(II) complexes.

The reference is the same as for the following sentence. We have added it here as well for completeness.

5. Page 3 line 85 - please look again at the discussion on the relationship between T1/2 and T(LIESST) which has been a point of discussion in the community since Letard's work in 2006 suggesting the opposite, i.e. that high T(LIESST) values require lower T1/2 values due to the inverse linear relationship between the two. Letard proposed that T(LIESST) = T(0) − 0.3T(1/2) in his article in J. Mater. Chem., 2006,16, 2550-2559, https://doi.org/10.1039/B603473J.

We are fully aware of Letard's empirical relationship $T_{\mathrm{LIESST}} = \mathrm{T}(0) - 0.3T_{1/2}$ as cited already in the submitted manuscript (new ref. 19). However, a first intrinsic prerequisite for observing LIESST is that the system is at a temperature below $T_{1/2}$, i.e. in the LS state. Hence, T_{LIESST} must always be below $T_{1/2}$. The relationship observed by Letard is a consequence of Marcus-type behaviour: low $T_{1/2}$ [corresponding to a low-energy HS state] leads to a *relatively* high T_{LIESST} value [corresponding to a higher barrier at a constant degree of distortion] (see Fig. 1B). For LIESST being observable around room temperature, for example, $T_{1/2}$ must be around room temperature at least. Consequently, a high $T_{1/2}$ around room temperature [corresponding to a very high-energy HS state] is required but this comes with a low barrier (Fig. 1B). This effect must be compensated by a larger distortion as illustrated in Fig. 1C.

6. Page 4, line 98 – explain why higher Mn-C covalency should significantly destabilize the HS state.

We have included the following to describe the effect: "The higher Mn–C covalency increases the σ-bonding strength, resulting in significant destabilization of the e_g* orbitals relative to the t_{2g} orbitals and consequently leads to a higher-energy HS state." And later: "Hence, our molecular design based on metal-ligand covalency as seen in the molecular orbitals (Supplementary Tables 3 and 4) … "

Reviewer #2 (Crystallography):

1. The HS-1 structure was collected while the crystal was continuously irradiated. Under these conditions, the observed HS-1 state may reflect a combination of HS-1 excitation and thermal effects from heating. The elevated APD could therefore partly originate from temperature effects, which may also influence bond lengths.

The lowest achievable stable temperature for the STOE STADIVARI diffractometer with nitrogen Oxford cooling system is the given experimentally determined temperature of 91 K. At 91 K, thermal **HS-1**→**LS-1** relaxation is slow, yet already noticeable (see Fig. 4B, Supplementary Figures 25/26). Hence, an XRD experiment with continuous LED irradiation was chosen to determine the photoinduced HS structure of **1**. Irradiation of the crystal was performed with a non-focused LED beam of 0.5 W power with an area of ca. 16 mm^2. Assuming homogenous power distribution, the light power density on the crystal is estimated to 0.31 mW mm^{-2}. The power acting on the largest face of the crystal with an area of ca. 0.031 mm^2, assuming a rectangle, is with 0.0096 mW very small. Consequently, only very weak to negligible heating of the crystal occurs due to efficient heat dissipation to the environmental oil (see also 10.1107/S0021889805009891). The elevated anisotropic displacement parameters (ADPs) stem from a disordered ligand, which could be resolved. The structural parameters of the HS structure are well reproduced by the optimized structure from DFT calculations, which clearly supports the structure representing essentially the HS state of **1** (see Supplementary Table 1). Precedence for the determination of photoinduced HS structures under continuous irradiation can be found in the literature (e.g. 10.1103/PhysRevB.78.134112). Precedence exists for pulsed continuous irradiation during XRD measurements with laser irradiation before each frame (e.g. 10.1002/chem.200802279).

Notably, significant residual electron densities (up to 0.67 $e^- Å^{-3}$) are present around atoms C32–C33–C34–C35–N14–C36–C37–N15–C38, indicating that the current structural model may require improvement.

We thank reviewer 2 for this valuable advice. We added a split atom model for the ligand to describe the disorder. This resulted in a significant improvement. The revised structures **LS-1** and **HS-1** are deposited in the CCDC.

For the HS-1 dataset, the data were cut down at 2theta 54°. However, both the I/σ(I) vs resolution and R_merge vs resolution plots suggest declining data quality at high angles. The authors should consider cutting down the data at 2theta 50°, consistent with IUCr recommendations, or collecting a higher-quality dataset.

We thank reviewer 2 for this advice and cut the data of **HS-1** at 2theta 50°. Unfortunately, no higher quality single crystals could be obtained to further improve the structure model.

2. *The authors state that after switching off the light source, the crystal retained the characteristic orange color of the HS-1 state. If so, it would be highly valuable to collect an additional HS-1 dataset with the light turned off to verify the stability and purity of this state.*

As mentioned above, the relaxation of photoinduced **HS-1** in the solid state slowly occurs already at 91 K (Supplementary Figures 25/26), the lowest possible temperature for XRD measurements in our setup. To avoid partial recovery of **LS-1** during the data collection for **HS-1**, the structure determination of **HS-1** had to be performed under constant irradiation. We clarified the cooling/irradiation/heating sequence in Supplementary Figure 28 as follows: "**Fig. S28.** Photographs of a single crystal of **1** before the XRD experiment (**A**), after irradiation at λ_{exc} = 560 nm for 2 minutes (**B**) and after 10 minutes (**C**) at 91 K. After the XRD experiment, the light source was switched off and the photograph was taken immediately (**D**), showing the intact orange crystal at 91 K. Subsequent heating of the crystal to 293 K was followed by cooling to 91 K (**E**)."

The authors should consider collecting an LS-1 dataset prior to the photocrystallography experiment. In the current LS-1 structure, atoms C32–C33–C34–C35–N14–C36–C37–N15–C38 exhibit elongated ellipsoids, suggesting unresolved disorder. This feature may reflect structural changes induced by photo- or thermally driven transformations rather than intrinsic LS-1 behavior.

Two independent single crystals were investigated to determine the structures of **LS-1** and **HS-1**. The structure of **LS-1** was determined without any irradiation, neither before or during the measurement. Hence, the disorder is an intrinsic characteristic and not induced by light irradiation. The elongated ellipsoids were due to unresolved disorder of one ligand, which is now considered in the revised structure model. We clarified this in the main text (line 153) and the Supplementary Material (Supplementary Table 1).

Minor suggestion: *All crystallographic geometric parameters discussed in the text, figures, and tables should include standard uncertainties (s.u.s) to allow proper assessment of data quality and statistical significance.*

The s.u.'s were already summarized in Supplementary Table 1. As suggested, we added the s.u.'s in the main text and to Fig. 2C.

Reviewer #3 (Comments for the Author):

This manuscript reports the preparation of a highly original tetracarbene Mn(II) complex of the pbmi ligand and the characterization of its spectrocopic and photomagnetic properties, in solution and in the solid, with state of the art techniques and setups. It follows the report by the same group of its Mn(I) analogue (10.1038/s41467-025-63225-4), here used as a precursor. The light-induced excited spin state trapping property of the Mn(II) molecular complex occurs in solution at a remarkably high temperature TLIESST=141 K, setting a new record. This is associated with a very long lifetime of the high spin state at all temperatures (e.g. microseconds at RT), incomparable with isoelectronic Fe(III) complexes, which makes these results of particular significance and impact. Rationalization of these properties relies on PES schematic representations followed by DFT modelling. The work is rigorous and thorough, as is the writing and the bibliography.

We thank this reviewer for the very enthusiastic and encouraging assessment of our study.

However a few aspects should be completed and clarified to consolidate the authors' conclusions. Questions:

1) One aspect that has not been considered as a handle to extend the HS lifetime in Fig. 1 is the steepness of the PES of the HS state, e.g the attached Fig. 1E: It is very helpful to decompose the effects of separate factors on the HS -> LS energy barrier, such as ΔE^0HL (Fig. 1C) and ΔQHL (Fig. 1D), but I think at this early stage of the manuscript, it would be interesting to consider an additional figure comparing a reference complex (e.g. $Mn^{II}N_6^{2+}$) and a more covalent one ($Mn^{II}N_2C_4^{2+}$). For the latter I would expect steeper PESs due to stiffer vibrational modes. At this stage, I'm not sure one could predict that this particular ligand set would fall in the Fig.1C and/or Fig.1D case (and/or the suggested Fig.1E), and which effect would take over (in 1C the HS -> LS barrier is smaller; in 1D and 1E it is larger). There may be some hints in the literature on Fe(II) analogues (or possibly Fe(III), if they were considered). Otherwise, the comparison with a model ML_6^{2+} Mn(II) compound having the same overall charge, same geometry (e.g. with two terdentate 2,6-bis(1,2,3-triazolyl)pyridine ligands) but an N6 coordination sphere instead of N2C4 should be very informative. In particular the comparison of the MO diagrams (and the Mn-L elongations in the HS state) should provide quantitative arguments for high covalence, which are currently lacking. Besides, the composition of the ds orbitals in terms of % of ligand contribution, 2nd the force constants for Mn-N and Mn-C bonds in LS1 and HS1, as compared to Mn-N force constants in the model MN_6^{2+} complex, should also be meaningful.*

We thank the reviewer for this thoughtful suggestion to consider the steepness of the HS and LS potential wells and the resulting effect on the activation barrier. We now address the rigidity of the five-membered ring chelating C^N^C ligand, that leads to narrower and stiffer potential wells (as compared to monodentate ligands, for example). However, it is difficult to quantitatively predict the effect on the force constants of the respective LS and HS PESs. We include this suggestion as follows in the main text: "These rigid ligands should also lead to steeper potential wells, i.e. larger force constants in the LS and HS states."

We have considered the suggestion of adding reference compounds to Fig. 1. However, unlike iron(II/III) SCO systems, there is only very scarce literature of LS manganese(II) complexes. To our knowledge no $Mn^{II}N_2C_4^{2+}$ complex has been reported to date. Furthermore, SCO has only been observed for manganocenes which possess a very different coordination environment. In addition, we would like to keep Fig. 1 as concept for our design rather than provide specific examples for manganese complexes (which do not yet exist). This conceptual illustration (Fig. 1) has also been appreciated by reviewer 4. Hence, we did not add specific complexes to the figure.

As suggested, we have now displayed the molecular orbitals of **LS-1** and **HS-1** in the new Supplementary Tables 3 and 4 to provide information on the Mn–L bond covalency in **1**. Significant ligand contributions are clearly visible in the singly occupied "dσ*" (e_g*) orbitals of **HS-1** (Supplementary Table 4).

2) Figure 5 displays photos of writing / erasing with light on a sample that was deposited on paper. Why is the green color of LS1 not apparent at the start of the experiment? Could it have been spontaneously reduced to the Mn(I) starting material, which is purple? In this experiment, could you be performing singlet-quintet Mn(I) light-induced SCO instead of doublet-sextet Mn(II) SCO? Do you think the paper sample would change color if it was exposed to oxidizing vapors? This question came back on the occasion of the single crystal experiment in Fig. S28-A and S28-E (see also line 549 "A color change from dark red to orange") (see also line 285). Could dark red be Mn(I)? Does this particular crystalline sample change color when exposed to oxidizing vapors? Could the solution phase favor the Mn(II) oxidation state, while the solid phase (powder or crystal) favors the Mn(I) oxidation state? Or could the conditions of the solution phase experiments favor Mn(II), while the conditions of the solid phase experiments favor Mn(I)? I'm confused because the stoichiometry in the cif files says MnII (1Mn:2PF6), and the photos in Fig.2D and 3A are green indeed.

We are sorry for the confusion that arises from the colour perception of **1** (by the human eye) at various temperatures. Indeed, the colour of the manganese(II) complex on paper in Fig. 5 appears dark red at 77 K. We have observed this colour change in solution as well (see Fig 3A) when cooling the complex solution to 130 K. This is reflected in slight band shifts and band narrowing at 95 K, which is a normal temperature effect (Supplementary Fig 14). There is no change of the overall absorption band pattern (that would hint for a reduction to manganese(I) or formation of other species), but simply band shifting and band narrowing that plays a trick to the human eye. We have clarified this thermochromic effect in Fig. 3/Fig. 5 and Supplementary Fig. 14 /Fig. 28. "**LS-1** appears brownish at low temperatures, which is a combination of slight band shifts and band narrowing at lower temperature (Supplementary Fig. 14) that leads to this color perception by the human eye."

Additional information that should be extracted from the available computational data :

1) Energies of all states (table or Jablonski diagram)

For completeness, we have added the energies of the geometry-optimized states to the new Supplementary Table 2. However, we refrain from further discussing the relative energies of the states in the manuscript since the energies of different spin states are often poorly predicted by DFT and highly dependent on the amount of considered exchange (see e.g. 10.1039/B419105F, p.220 ff).

2) S^2 values for LS1, HS1, IS1 (^{4}MC), ^{4}MLCT and TD-DFT-optimized excited states (^{2}MLCT, ^{6}MLCT)

For completeness, we have also added the spin expectation values S^2 to the new Supplementary Table 2. However, these are values are poorly predicted via DFT and we refrain from further discussion. This is also noted in the ORCA output: "*Warning: in a DFT calculation there is little theoretical justification to calculate <S**2> as in Hartree-Fock theory. We will do it anyways but you should keep in mind that the values have only limited relevance.*"

Suggested modifications / clarifications :

- Fig 1A and 1B could be merged

Thank you for this valuable suggestion. We have merged 1A/1B.

- In Fig 3 it seems the photos are reversed (LS1 is green, HS1 is pale orange)

The photos are correct, the color of **LS-1** changes upon cooling to red-brown (see discussion above). We clarify this in the manuscript to avoid confusion.

- line 19 : high metal-ligand covalence, instead of strong

We thank the reviewer for his suggestion. We have changed this in the manuscript abstract.

- line 28 : specify "switched by light from singlet closed-shell to diradicaloid states"?

We have added the spin multiplicities for the respective states as follows: "singlet closed shell (S = 0) to diradicaloid states (S = 1)".

- line 40 : I find that the sentence "Fig. 1A depicts... for the d5 case" interrupts the reasoning. I would delete it and simply quote Fig. 1A together with Fig. 1B in line 55.

We thank the reviewer for this advice. The sentence was deleted, hoping that the reasoning is now more fluent to the reader.

- line 44 : the HS state doesn't have "more degrees of freedom" but it has softer degrees of freedom

The reviewer is absolutely correct. We have corrected this in the manuscript.

- line 89 : "smaller and narrower barrier" Ea,HL' (prime is missing)

We thank the reviewer for spotting this. We added the prime to $E_{a,HL}$ and to ΔE^0_{HL}.

- line 102 : "we hypothesized that manganese (II) ions" could also form

"can" was changed to "could".

- line 107 : "large nuclear reorganization energy" lHL' (prime is missing)

We added the prime to the reorganization energy λ_{HL}.

- line 112 : this sentence sounds like this particular Mn(II) complex provides the favorable ΔS=2 situation, but isn't a LS -> HS transition by definition associated with ΔS=2?

Only octahedral d^5 and d^6 metal complexes deliver a ΔS=2 spin change between LS and HS states. Octahedral d^4 and d^7 metal complexes (e.g. chromium(II) or cobalt(II)) deliver only ΔS=1. Hence, we would like to leave this sentence in the manuscript.

- line 115 : LS-HS energy gaps are highly *functional-dependent* (I wouldn't say they are not reliably predicted by DFT methods)

We have modified this statement as suggested: "Although energy differences of states with different multiplicity are highly functional-dependent[34-37], …"

- line 133 : bright state 5 should be state 8 (cf table S4)

We thank the reviewer for checking the manuscript so thoroughly and spotting this mistake. We have corrected the number of the bright state in the figure caption to 8.

- line 149 : the strong σ-donating character? (instead of σ-bonding character) of the carbenes

We changed it to "σ-donating" as suggested.

- lines 179-180 and 212 : yr is the conventional abbreviation for year

(https://www.cas.org/training/documentation/references/cas-standard-abbreviations#abbrlisting-

We thank the reviewer for this suggestion. However, we didn't want to use the abbreviation for year but rather the unit. In the *IUPAC Compendium of Chemical Terminology* we found that the unit for year is "a".

IUPAC Compendium of Chemical Terminology, 5th ed. International Union of Pure and Applied Chemistry; 2025. Online version 5.0.0, 2025. https://doi.org/10.1351/goldbook.Y06723

- In Fig 3 it seems the photos are reversed (LS1 is green, HS1 is pale orange)

The photos are correct, the color of **LS-1** changes upon cooling to red-brown (see discussion above). We clarify this in the manuscript to avoid confusion: "The photographs show frozen solutions of **1** in 2-MeTHF/MeCN (29:1 *v*/*v*) after (**HS-1**, pale orange-yellow) and before (**LS-1**, red) steady-state irradiation (2 min) with a 560 nm LED at 130 K."

- In Fig. 3A and 3D the green-to-blue color gradient is probably the default but using the actual green/orange compound colors would seem more didactic to me (also on Fig. S14 and S16, Fig. 4B and S25)

We thank the reviewer for this didactic suggestion. In fact, we had considered this color code for the LS and HS states as well. However, we wanted to take color blind (green/red) readers into account and decided for the blue/green color code instead. This is also one of the nature manuscript guidelines. (see: https://research-figure-guide.nature.com/figures/preparing-figures-our-specifications/#colour-space)

- Aren't all excitation wavelengths equal to 560 nm (and observation wavelengths equal to 590 nm)? Cf caption Fig 3B, 3C

The laser excitation wavelength for the TA experiments was 590 nm at the absorption maximum. The excitation wavelength for the low-temperature absorption spectra was 560 nm from a UHP-LED which has a non-tunable emission energy. The spectral evolution (Fig 3A, inset) is shown at the absorption maximum (590 nm).

- line 222 : you could recall the conditions 293 K (fluid solution), 130 K (solution?), 77 K (frozen medium)

Good suggestion. We have added this information for the 293 K and 130 K temperatures, as suggested: "at 293 K (fluid solution), 130 K (frozen solution)". For the extrapolated value at 77 K, we cannot give a medium, since the very long lifetime (> 10^6 a) impedes a meaningful measurement.

- line 230 : the sentence "a kinetic model with two excited states [...] is insufficient" (the dynamics are fitted with four time constants) should be rephrased. If some of these timescales refer to vibrational relaxation within the same electronic state, maybe 2 excited states are sufficient. I wouldn't directly relate the number of time constants with the number of excited states involved.

The reviewer is certainly correct by stating that the number of time constants does not necessarily relate to the number of involved states as cooling might give extra time constants. However, we know that we have at least 3 discernible intermediate excited states. The EADS from the fs-TA experiments reveal an early excited state with strong absorption in the visible region, which then converts to another excited state with a very different spectrum followed by a thermally activated conversion to the HS state with a barrier of 0.11 eV (τ_4). Thus at least three intermediate excited states can be spectrally and kinetically distinguished. We have included a schematic diagram (Supplementary Fig. 21) to illustrate our model. Yet, an assignment of the states' character and energy is not possible with the present data.

- line 251 : "losses occur" (if I understand correctly, you could refer to ISC' and ISC" on Fig. 1)

We have considered *all* loss channels from (intermediate) excited states in Supplementary Fig. 21, not only from a single intermediate state (ISC', ISC'') as shown in the simplistic diagram in Fig. 1. From the TA spectra intensity loss occurs already at early stages. Hence, we deduce that losses can occur during the entire dynamics to the HS state.

- line 261 : "in the solid state" : meaning amorphous powder or microcrystalline solid, as opposed to frozen solution?

Thank you for this remark. We have specified that we used a microcrystalline solid sample.

- line 268 : "is lower than that determined in solution" (recall the value of 141 K?)

Excellent suggestion. We have added this information.

- line 311 : LIESST instead of LIEEST

Thank you for spotting this typo. Corrected.

- line 314 : "In solution [...] the TLIESST value amounts to 141 K and the lifetime [...] to over one million years at 77 K". I find it a little confusing to read in the same sentence a room-temperature property in solution and a low temperature property in frozen medium. Maybe you could rephrase.

We have rephased this part as follows (3 sentences): "In solution, the high-spin state forms quantitatively under visible light irradiation. The extrapolated lifetime of the high-spin state at 77 K amounts to over one million years. The T_{LIESST} value of 141 K exceeds previously reported T_{LIESST} values of iron complexes."

- line 475 the Methods section appears in the middle of the bibliography

We combined the two reference lists.

- line 620 : *after* excitation (not upon)

This was corrected in the manuscript.

- line 636 : "as described for the RT measurements" : does this refer to line 600 or line 629?

It refers to both measurements, because we performed two independent temperature-dependent measurements. One for the short timescales (0.1 eV barrier) and one for the long timescale (0.36 eV barrier). We clarified this in the text.

- line 637 : for all TA spectroscopy experiments

We thank the reviewer for this hint. We clarified this in the text.

- line 638, DFT calculations :

• I suppose the lowest ^{2}MLCT state is the 3rd excited state at the LS1 geometry (hence iroot3).

We clarified this as follows: "The excited doublet and sextet MLCT states ^{2}MLCT(3) and ^{6}MLCT(1) (keywords *iroot 3* and *iroot 1*, respectively) were geometry optimized using the number of roots keyword *nroots 7* (^{2}MLCT(3)) and *nroots 5* (^{6}MLCT(1)) respectively, following the root (keyword *followiroot true*) in combination with the same configuration as above (keywords *tightopt*, *tightscf*, *RIJCOSX*, *CPCM*(acetonitrile), *ZORA* and *D3BJ*)."

How did you access the ^{6}MLCT state by TD-DFT? Did you run a sextet single point calculation at the LS1 geometry and TD-DFT optimized from there, or did you start from the HS1 geometry? This should be specified

We clarified this as follows: "The ^{6}MLCT(1) geometry optimization was based on TDDFT calculations from the **HS-1** geometry, whereas the ^{2}MLCT(3) geometry optimization was based on the TDDFT calculations from the **LS-1** geometry."

• "Geometry optimizations were performed Atom-pairwise dispersion correction was performed"?

Thank you for spotting this mistake. Correction done.

• why was the def2-SVP basis set used in the specific case of ^{4}MLCT optimization? Or was it used for all TD-DFT MLCT optimizations? This should be clarified

The def2-SVP basis set was only used for the ^{4}MLCT state. When using the larger def2-TZVP basis set as used for all other states, the geometry converged to the ^{4}MC state. We added this as follows: "Increasing the basis set to *def2-TZVP* followed by optimization of the *def2-SVP*-optimized geometry converged to the ^{4}MC state, so that the ^{4}MLCT state was not accessible with the larger basis set, even with reduced step sizes."

• why were the calculated transitions shifted by 0.4 eV for LS1 and 0.1 eV for HS1? Was it to match the experimental band shapes?

Yes, the energy shifts were done to match the experimental data. We have added this to the manuscript: "The reported calculated transition energies are shifted by 0.4 eV and 0.1 eV to lower energies to match the experimental absorption spectra for **LS-1** and **HS-1**, respectively (Tables S7 and S8)."

• I suppose the TDDFT calculations were TDA-TDDFT. If so, it should be specified

Yes, TDA-TDDFT was used as it is set as a default in the ORCA package. https://www.faccts.de/docs/orca/5.0/tutorials/spec/UVVis.html. We have included this as follows: "TDDFT calculations were performed using the TDA approximation which is set as a default in ORCA 5.0.3.."

- refs 78, 79, 80 are not used in this work (basis sets for heavy elements)

In the ORCA output file of the calculation all these references are listed (although not used). We have removed these three references. We now use ref. 81 (10.1039/B515623H) for the auxiliary basis sets.

- ref 86 should probably be https://doi.org/10.1063/1.5143076

We have additionally added this reference (ref. 88) for the Theodore program.

- Captions of Table 1 and Fig 21: "obtained from DFT" *and TDA-TDDFT* calculations

Thank you for spotting this. We have added "TD(DFT)" to the respective tables.

- last column of Tables S4, S5 : the "dominant character" of the transitions could be enhanced by setting a threshold, e.g. 10% (as a side remark, I find it surprising that the MC character of states 1 and 2, representing the triply degenerate ground state, is only about 50%, and that the character of state 4, purely red on the metal and purely blue on the ligands, is not more than 60% MLCT).

Thank you for this consideration. We have double-checked all the contributions, which are correct. We suppose that the covalent M-L bonds challenge a clear distinction between CT and MC states which would be formally ionic descriptions. We have highlighted the dominant character in the respective tables in bold as suggested.

Reviewer #4 (Comments for the Author):

1. The manuscript reports a Mn(II) carbene complex that exhibits LIESST with remarkably slow HS to LS relaxation kinetics. By increasing metal–ligand covalency, the authors propose a strategy to simultaneously enlarge the electronic energy gap and the structural reorganization energy, thereby increasing the activation barrier for relaxation.

2. The work is conceptually original in extending LIESST behavior to a Mn(II) carbene platform and in explicitly using metal–ligand covalency as a design parameter to modulate photomagnetic relaxation. Most prior LIESST studies have focused on Fe(II) or Fe(III) systems. Reports of Mn-based LIESST are rare, and the deliberate manipulation of structural reorganization energy via covalency represents a meaningful conceptual advance.
The proposed covalency control strategy may provide a new direction beyond traditional the activation barrier tuning approaches and is therefore significant for the photo-switching.
3. The approach is appropriate and generally well executed.
The magnetic and spectroscopic data appear of high quality. Structural changes between spin states are clearly supported by both experiment and computation.
Figures are generally clear and logically organized. The conceptual figure is particularly effective.
We very much appreciate the very positive assessment.

Pls check following points.

The Arrhenius equation and LIESST misspelling.

Thank you very much for spotting this mistake and typo. We have corrected eq. 1 and the typo in the manuscript.

4. While activation energies and rate constants are reported, uncertainties are not systematically propagated in the comparison between solution and solid-state barriers and fitted Arrhenius parameters.

We address this below.

5. The qualitative conclusions, namely that increased covalency increases structural reorganization energy and slows relaxation, are robust and well supported by structural and spectroscopic evidence.

6. Authors should re-consider following.

6-1. Arrhenius fits including confidence intervals for extrapolated lifetimes.

Thank you for this suggestion. We have calculated the 95%-prediction interval of the HS lifetime extrapolated with the Arrhenius fit to 77 K. We included the experimental value from low temperature absorption spectroscopy (at 130 K) to the data set of the Arrhenius fit to have an experimental value closer to the extrapolated value at 77 K. This data point perfectly fits to the linear regression (Supplementary Fig. 13). The 95% prediction interval was then calculated according to equation 4 (Methods section) to $\tau_{77K,min}$ = 2.8×10^6 a and $\tau_{77K,max}$ = 4.0×10^6 a. In the main text, we simply state “over one million years”, as the exact number and prediction interval do not really matter in this case.

The following was added to the methods section

Calculation of prediction intervals. The 95%-prediction interval of the HS lifetime extrapolated with the Arrhenius fit to 77 K (Supplementary Fig. 13) was calculated using eq. 4 with $q_{0.975}^{t_{n-2}}$ and n being the quantile of the t-distribution and the number of data points, respectively.

$$PI_{0.95} = \hat{\alpha} + \hat{\beta} x_0 \pm \hat{\sigma} \cdot q_{0.975}^{t_{n-2}} \sqrt{1 + \frac{1}{n} + \frac{(x_0 - \bar{x})^2}{\sum_{i=1}^{n}(x_i - \bar{x})^2}} \quad \text{(eq. 4)}$$

$\hat{\alpha}$ and $\hat{\beta}$ denote the intercept and the slope of the linear fit function, respectively. x_i, x_0 and $\bar{x}$ are the position of individual data points, the position of the prediction and the mean value of the positions of the data set.

6-2. Additional relaxation data at lower temperatures.

This reviewer is perfectly right, that more data points would be desirable. However, physical constraints limit the measurable temperature / time windows: Lifetime measurements by TA spectroscopy below 273 K are impeded as no decay would be observable during the available time window. Measuring of lifetimes by absorption spectroscopy (as shown with the data point at 130 K) for example at 140 K or 120 K is impeded as the decay is already too fast and too slow, respectively. 130 K is indeed a “sweet spot” for this technique in this particular system. The drastic change in lifetimes with temperature is of course a result of the barrier of 0.36 eV, which results in a very steep slope of the Arrhenius curve. Yet, the excellent match of the linear relation for the two techniques at 130 K and 273 – 308 K temperature ranges and the small prediction interval provides already strong and robust arguments.

6-3. Residual plots or goodness-of-fit metrics for kinetic modeling.

Thank you for this suggestion. We have added the coefficient of determination values R^2 for the respective fits in the Supplementary Material (Fig. 17–Fig. 20). In all cases, $R^2 > 0.999$ confirming reliable fits.

6-4. A clearer kinetic scheme integrating ultrafast time constants.

We have added a qualitative kinetic scheme (Supplementary Fig. 21) with qualitative energy levels to provide a clearer picture of the ultrafast kinetics. Assignment of the time constants to individual processes between states of defined character is not possible based on the TA data alone. Yet, we known that at least three intermediate states are populated, that a small barrier exists before reaching the HS state (τ_4) and that losses can occur during the entire dynamics.

7. The manuscript appropriately references foundational LIESST and SCO literature.

Thank you for this assessment. As suggested by reviewer 1, we have added the classical McGarvey experiment (new ref. 18). Furthermore, we would like to add a reference to an iron(II) complex with a 0.33 eV HS→LS barrier for comparison (new ref. 44).

8. The manuscript reports a conceptually important and likely publishable advance. However, because the central quantitative claim relies on extrapolation and lacks full statistical treatment, additional quantitative rigor is required before acceptance in NC. With these revisions, the work would represent a strong and influential contribution to photo-switching materials.

In our view, the central statements of the manuscript are:

1) LIESST by a manganese(II) complex based on strong M-L covalency
2) high T_{LIESST} values
3) high HS→LS barrier
4) large distortion of the HS state
5) very high HS lifetimes at all temperatures (µs at 293 K; hours at 130 K; above one million years at 77 K).

All statements are backed up by experiments and calculations. The 95% prediction interval, as suggested by the reviewer, is very small, so that even the extrapolated lifetime can be safely assumed to be very high (above one million years).

We thank reviewer 3 for the suggestions, which we have considered as follows.

1. *The covalency of the Mn-C bonds: line 128, "as seen in the molecular orbitals"*
*Visual inspection of the LUMO+5 and LUMO+12 of **LS1** (Suppl. Table 3) (or HOMO/HOMO-1) of **HS1**, Supp. Table 4) does not allow to conclude on this matter. Mn-N bonds are much more ionic than Mn-C bonds, yet their contribution to the dσ* orbitals looks exactly the same. On the other hand, for LS1, HOMO-2 as well as HOMO-8 (currently not shown) highlight significant mixing between metallic and carbon-based orbitals (σ and π-bonding). You could be more specific in the text, and add HOMO-8 in Supplementary Table 3.)*
We thank the reviewer for her/his thorough thoughts regarding the bond covalency in **1**. In our opinion, we can conclude this from the molecular orbitals' inspection. With an orbital overlap argument, σ-bonding effects in covalency are more pronounced compared to π-bonding effects, especially for rather pure σ-donors, such as N-heterocyclic carbenes. Hence, we consider only the σ orbitals. The energy of the d_{x2-y2} orbital (LUMO+12), which is located in the MnC_4 plane, is higher by 0.83 eV compared to the energy of the d_{z2} orbital (LUMO+5) pointing to the axial nitrogen donors. This suggests that the Mn–C covalency is significantly higher than the Mn–N covalency. We have clarified the sentence as follows: "…as seen in the energies of the d_{x2-y2} and d_{z2} molecular orbitals (Supplementary Tables 3 and 4)…". As suggested by the reviewer, we have extended the Supplementary Tables 3 and 4 and added HOMO-8 and HOMO-13/14, respectively.

2. *Line 101, "destabilization of the e_g* orbitals relative to the t_{2g} orbitals"*
*Out of curiosity, I optimized one MnN_6^{2+} complex in its LS and HS states (cartesian coordinates at the end of this document). Given that the computational protocols used by the authors correctly predicts a low spin ground state for complex **1** despite the known HS overstabiliziation by B3LYP, I assumed state ordering would also be predicted correctly for the model complex. In this case, as expected from the weaker ligand field, a high spin ground state is obtained. The orbitals of **LS1** and LS-tz can then be compared.*

	LS1 (ground state)	LS-tz
Gibbs free energy	-2717.08549364 Eh	-2624.18170363
HOMO	-5.53 eV	-6.17
e_g1	-0.03 eV (LUMO+5; MnN_2)	-0.52 (LUMO+8; MnN_4)
e_g2	+0.80 eV (LUMO+12; MnC_4)	-0.12 (LUMO+9; MnN_2)
HOMO-e_g1	5.50 eV	5.65
HOMO-e_g2	6.33 eV	6.05
e_g1-e_g2	0.83 eV	0.40

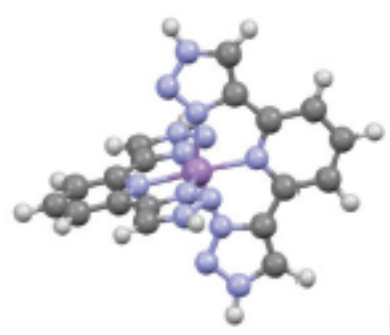
LS-tz

*It appears that the HOMO-e_g1 gap is actually slightly smaller in **LS1** than in LS-tz, but that the HOMO-e_g2 gap (involving the carbon atoms) is significantly larger. Both being singly occupied in the HS state, the destabilization of HS1 seems to be due to the larger HOMO-e_g2 gap in LS1. Maybe you could be more specific in the text. (and possibly use the MN_6^{2+} model compound)*
We truly appreciate the effort made by the reviewer and thank her/him for the additional DFT calculations of the proposed MnN_6^{2+} model complex. These calculations further substantiate our design strategy with the strong Mn-C covalency due to strong σ-(anti)bonding, resulting in the higher energy of the σ-antibonding d_{x2-y2} orbital. Yet, we believe that including this model complex would shift the primary focus of the manuscript and might distract the reader. Yet, we encourage the reviewer to analyse the present system in comparison with other suitable Mn systems by quantum chemical calculations. We suggest that such an analysis of the bonding situation is worth a separate full publication. Yet, we have tried to be more specific about the effects caused by the Mn-C covalency and made the following addition to the manuscript: "In **LS-1**, the d_{x2-y2}-derived e_g* orbital lying in the MnC_4 plane is even higher in energy than the d_{z2} orbital pointing along the N–Mn–N axis by 0.83 eV so that population of this orbital in the HS state would imply a large energy penalty."

3. *Line 129 "higher displacements Q_{HL}; Bond elongations between LS and HS forms were compared for HS1 and HS-tz. Elongations in complex 1 indeed surpass the ones in MnN62+ complex. As already noted by the authors by comparing with literature data on iron complexes (because there is*

no comparable Mn^{II} complexes in the literature.), the +0.4 Å Mn-N elongation is outstanding:

	HS1	HS-tz (ground state)
Gibbs free energy	-2717.08140976 Eh	-2624.22303696 Eh
Mn-N	+0.400	+0.256; +0.265; +0.228
Mn-C	+0.278	-

Again, we appreciate the analysis of the reviewer. The bond elongations (especially the Mn–N bonds) in the present system are truly outstanding, and we thank the reviewer for the comparison with the HS-tz model complex. This again highlights the stronger distortion upon introduction of carbenes, which form covalent Mn–C bonds. In our opinion, further elongation of Mn–C bonds might be hindered by the rigid 5-membered chelate ring of the pincer-type ligand, resulting in a smaller Mn–C bond elongation as compared to Mn–N. A smaller displacement of the terminal donor atoms in the HS state has also been calculated for $[Fe(tpy)_2]^{2+}$ possibly due to the ring strain (10.1016/j.ccr.2005.12.006). Yet, these observations must be computationally and experimentally verified in the future with suitable systems.

4. *Wrong compund color in line 218-219*

   The thank the reviewer for spotting the mistake. This has been corrected "… red to faint orange …".

5. *If I understand correctly, HS-1 is pale orange at all temperatures (bleach in Fig. 3 and Supp. Fig. 14) discoloration in Figure 5, orange crystals in Supp. Fig. 28. For clarity, it may be worth saying it explicitly.*

   We have observed the color of HS-1 by eye only at low temperatures as its lifetime at room temperature is too low. Yet, we were able to extract the spectrum of **HS-1** at room temperature from TA data (Supplementary Figure 22). This is shifted relative to the spectrum at low temperature so a weak thermochromic (and/or solvatochromic) effect might be operative. But we cannot tell the color of HS-1 at room temperature that is perceived by the human eye.

6. *Finally, photoswitching is complete after 2 min of irradiation in solution at 95-130 K (Figure 3) and on paper at 77 K (Figure 5). However, a longer irradiation time is required to photoswitch the single crystals at 91 K, as shown by the comparison between photos B (2min) and C (10 min) in Supp. Fig. 28. Maybe this can be mentioned around line 296, as it echoes the discussion on cooperative effects in the microcrystalline solid used in the SQUID measurement (line 283)*

   We are grateful for this observation on the different irradiation times. In all cases, we irradiated longer than necessary to ensure complete conversion, and we used different light intensities (light source – sample distances) so that a meaningful (semiquantitative) comparison cannot be made it this point. But yes, it appears faster in solution than in the solid, which might be due to cooperatively in the solid. But without more quantitative data we would like to avoid an overinterpretation of the data.

Although the CIF files pass CheckCIF without level A/B alerts, if these structures play a central role in the manuscript, the authors should refine the models more carefully or collect higher-quality datasets by redesigning their photocrystallography experiment.

Photocrystallography experiment (Page 16, lines 547–556):

1 The HS-1 structure was collected while the crystal was continuously irradiated. Under these conditions, the observed HS-1 state may reflect a combination of HS-1 excitation and thermal effects from heating. The elevated APD could therefore partly originate from temperature effects, which may also influence bond lengths. Notably, significant residual electron densities (up to 0.67 e^{-} $Å^{-3}$) are present around atoms C32–C33–C34–C35–N14–C36–C37–N15–C38, indicating that the current structural model may require improvement.

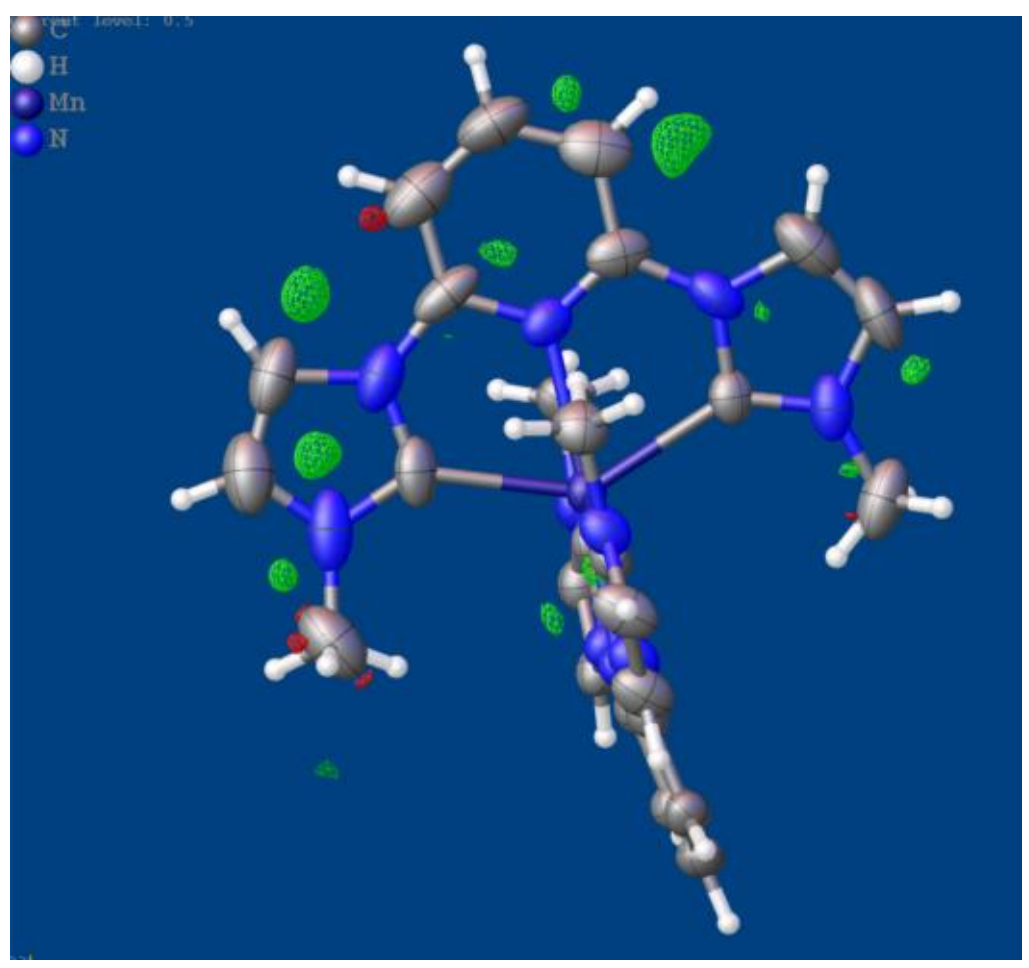

For the HS-1 dataset, the data were cut down at 2theta 54°. However, both the I/σ(I) *vs* resolution and R_merge *vs* resolution plots suggest declining data quality at high angles. The authors should consider cutting down the data at 2theta 50°, consistent with IUCr recommendations, or collecting a higher-quality dataset.

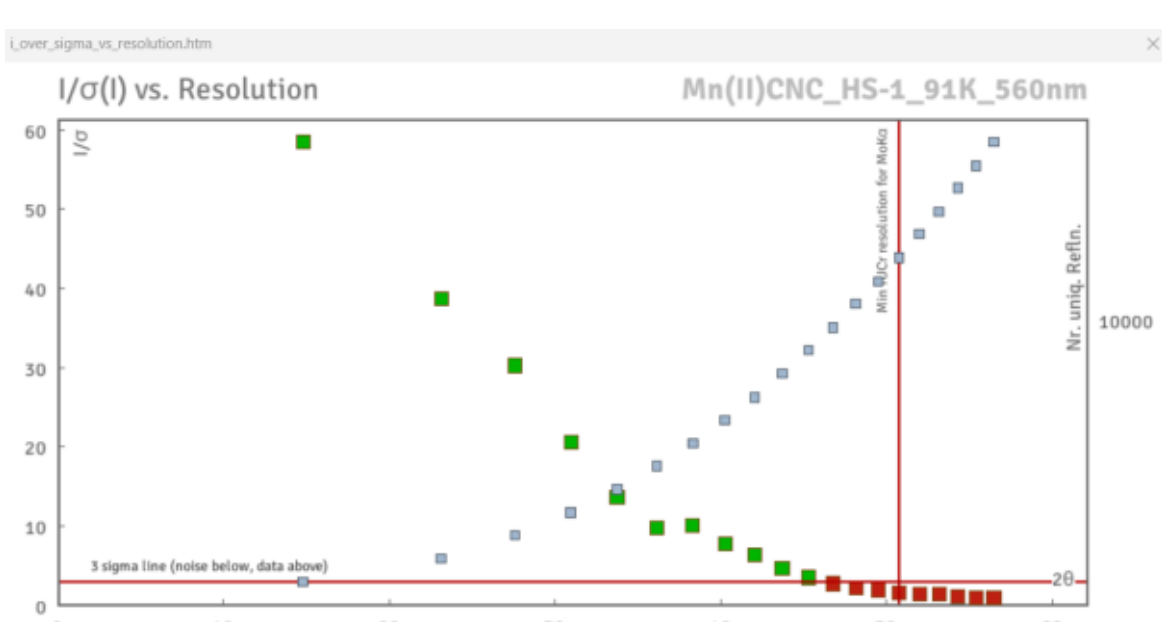

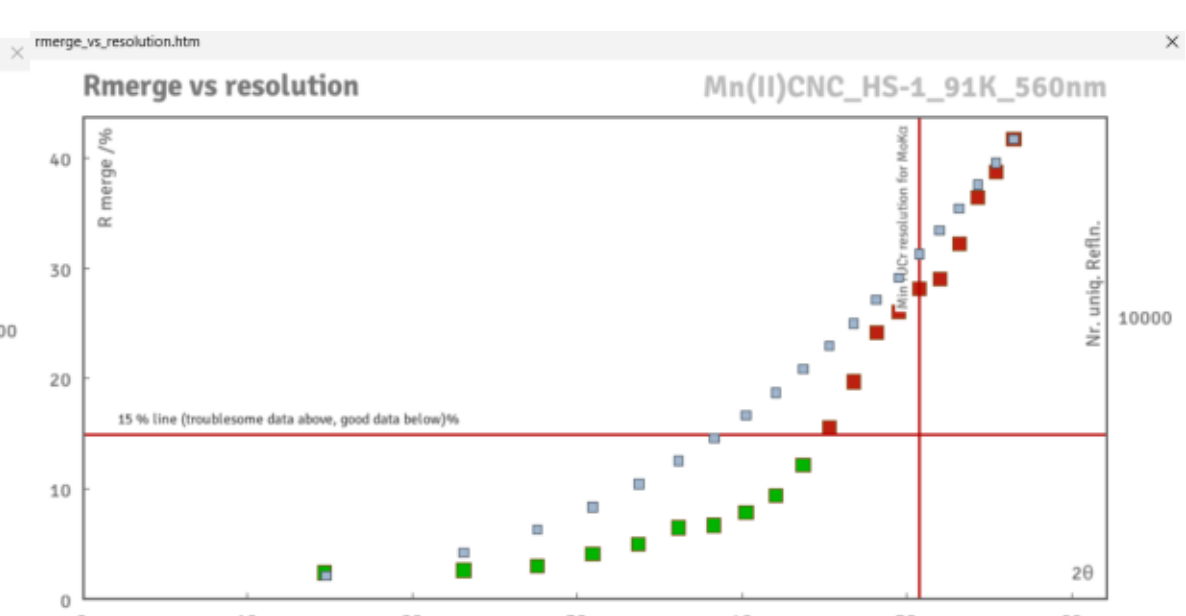

2 The authors state that after switching off the light source, the crystal retained the characteristic orange color of the HS-1 state. If so, it would be highly valuable to collect an additional HS-1 dataset with the light turned off to verify the stability and purity of this state.

3 The authors should consider collecting an LS-1 dataset prior to the photocrystallography experiment. In the current LS-1 structure, atoms C32–C33–C34–C35–N14–C36–C37–N15–C38 exhibit elongated ellipsoids, suggesting unresolved disorder. This feature may reflect structural changes induced by photo- or thermally driven transformations rather than intrinsic LS-1 behavior.

Minor suggestion:

All crystallographic geometric parameters discussed in the text, figures, and tables should include standard uncertainties (s.u.s) to allow proper assessment of data quality and statistical significance.

Review on manuscript NCHEM-26010166-T by Kronenberger et al.
"**Covalency control of photomagnetic relaxation in a manganese photoswitch**"

This manuscript reports the preparation of a highly original tetracarbene Mn(II) complex of the pbmi ligand and the characterization of its spectrocopic and photomagnetic properties, in solution and in the solid, with state of the art techniques and setups. It follows the report by the same group of its Mn(I) analogue (10.1038/s41467-025-63225-4), here used as a precursor.
The light-induced excited spin state trapping property of the Mn(II) molecular complex occurs in solution at a remarkably high temperature T_{LIESST}=141 K, setting a new record. This is associated with a very long lifetime of the high spin state at all temperatures (e.g. microseconds at RT), incomparable with isoelectronic Fe(III) complexes, which makes these results of particular significance and impact. Rationalization of these properties relies on PES schematic representations followed by DFT modelling. The work is rigorous and thorough, as is the writing and the bibliography. However a few aspects should be completed and clarified to consolidate the authors' conclusions.

Questions :

1) One aspect that has not been considered as a handle to extend the HS lifetime in Fig. 1 is the steepness of the PES of the HS state, e.g the attached Fig. 1E :

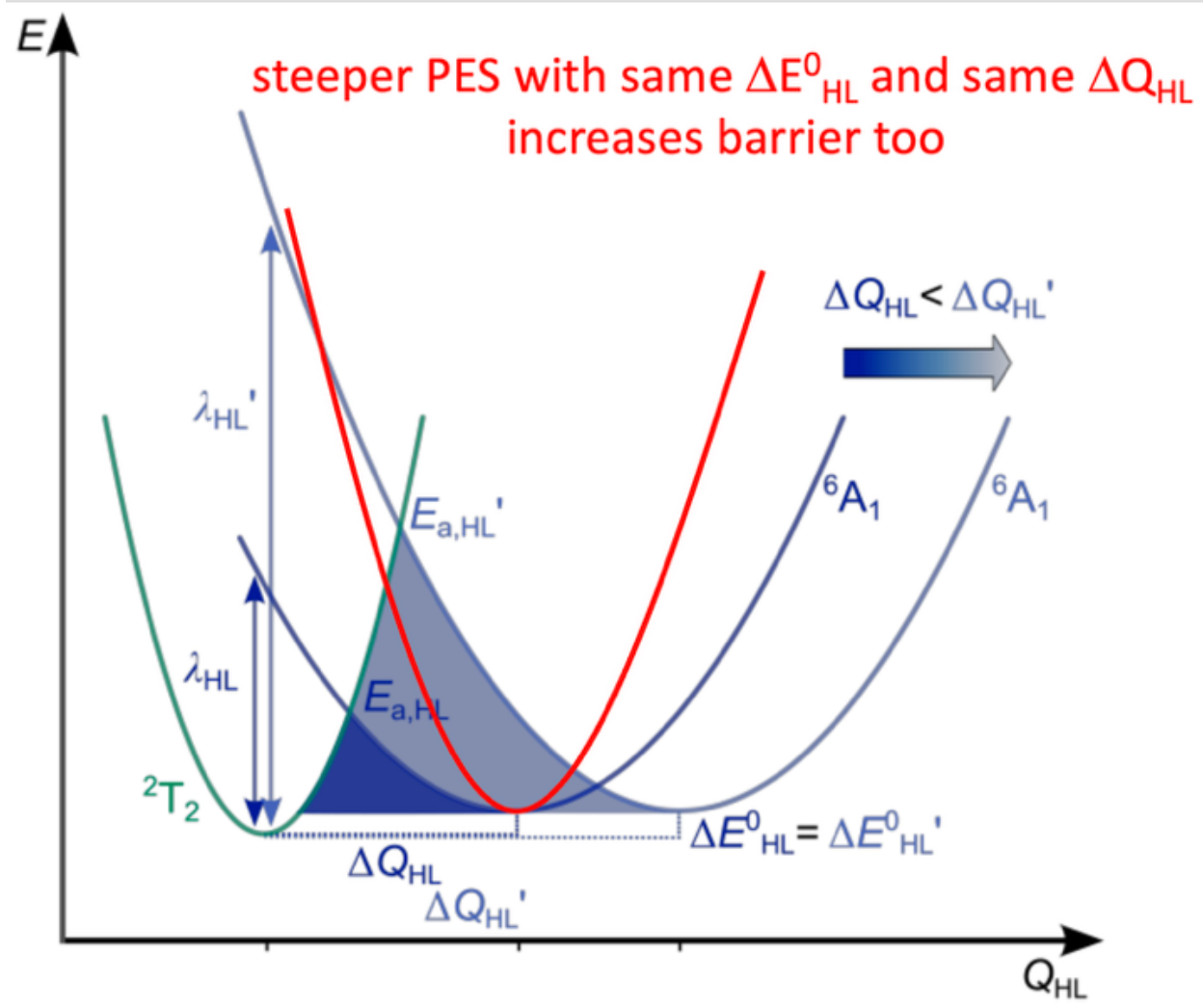

It is very helpful to decompose the effects of separate factors on the HS -> LS energy barrier, such as ΔE^0_{HL} (Fig. 1C) and ΔQ_{HL} (Fig. 1D), but I think at this early stage of the manuscript, it would be interesting to consider an additional figure comparing a reference complex (e.g. $Mn^{II}N_6^{2+}$) and a more covalent one ($Mn^{II}N_2C_4^{2+}$). For the latter I would expect steeper PESs due to stiffer vibrational modes. At this stage, I'm not sure one could predict that this particular ligand set would fall in the Fig.1C and/or Fig.1D case (and/or the suggested Fig.1E), and which effect would take over (in 1C the HS -> LS barrier is smaller; in 1D and 1E it is larger). There may be some hints in the literature on Fe(II) analogues (or possibly Fe(III), if they were considered). Otherwise, the comparison with a model ML_6^{2+} Mn(II) compound having the same overall charge, same geometry (e.g. with two terdentate 2,6-bis(1,2,3-triazolyl)pyridine ligands) but an N_6 coordination sphere instead of N_2C_4 should be very informative. In particular the comparison of the MO diagrams (and the Mn-L elongations in the HS state) should provide quantitative arguments for high covalence, which are currently lacking. Besides, the composition of the dσ* orbitals in terms of % of ligand contribution,

and the force constants for Mn-N and Mn-C bonds in LS1 and HS1, as compared to Mn-N force constants in the model $Mn^{II}N_6^{2+}$ complex, should also be meaningful.

2) Figure 5 displays photos of writing / erasing with light on a sample that was deposited on paper. Why is the green color of LS1 not apparent at the start of the experiment? Could it have been spontaneously reduced to the Mn(I) starting material, which is purple? In this experiment, could you be performing singlet-quintet Mn(I) light-induced SCO instead of doublet-sextet Mn(II) SCO? Do you think the paper sample would change color if it was exposed to oxidizing vapors?
This question came back on the occasion of the single crystal experiment in Fig. S28-A and S28-E (see also line 549 "A color change from dark red to orange") (see also line 285). Could dark red be Mn(I)? Does this particular crystalline sample change color when exposed to oxidizing vapors?
Could the solution phase favor the Mn(II) oxidation state, while the solid phase (powder or crystal) favors the Mn(I) oxidation state? Or could the conditions of the solution phase experiments favor Mn(II), while the conditions of the solid phase experiments favor Mn(I)?
I'm confused because the stoichiometry in the cif files says Mn^{II} (1Mn:2PF_6), and the photos in Fig.2D and 3A are green indeed.

Additional information that should be extracted from the available computational data :
1) Energies of all states (table or Jablonski diagram)
2) S^2 values for LS1, HS1, IS1 (^{4}MC), ^{4}MLCT and TD-DFT-optimized excited states (^{2}MLCT, ^{6}MLCT)

Suggested modifications / clarifications :
- Fig 1A and 1B could be merged
- $\Delta E_{HL}{}^0$ should read $\Delta E^0{}_{HL}$ throughout (as in Fig. 1)
- line 19 : *high* metal-ligand covalence, instead of strong
- line 28 : specify "switched by light from singlet *closed-shell* to diradicaloid states"?
- line 40 : I find that the sentence "Fig. 1A depicts... for the d^5 case" interrupts the reasoning. I would delete it and simply quote Fig. 1A together with Fig. 1B in line 55.
- line 44 : the HS state doesn't have "more degrees of freedom" but it has *softer* degrees of freedom
- line 89 : "smaller and narrower barrier" $E_{a,HL}$' (*prime* is missing)
- line 102 : "we hypothesized that manganese (II) ions" *could* also form
- line 107 : "large nuclear reorganization energy" λ_{HL}' (*prime* is missing)
- line 112 : this sentence sounds like this particular Mn(II) complex provides the favorable $\Delta S=2$ situation, but isn't a LS -> HS transition by definition associated with $\Delta S=2$?
- line 115 : LS-HS energy gaps are highly *functional-dependent* (I wouldn't say they are not reliably predicted by DFT methods)
- line 133 : bright state 5 should be state *8* (cf table S4)
- line 149 : the strong σ-*donating* character? (instead of σ -bonding character) of the carbenes
- lines 179-180 and 212 : *yr* is the conventional abbreviation for year (https://www.cas.org/training/documentation/references/cas-standard-abbreviations#abbr-listing-a)
- In Fig 3 it seems the photos are reversed (LS1 is green, HS1 is pale orange)

- In Fig. 3A and 3D the green-to-blue color gradient is probably the default but using the actual green/orange compound colors would seem more didactic to me (also on Fig. S14 and S16, Fig. 4B and S25)
- Aren't all excitation wavelengths equal to 560 nm (and observation wavelengths equal to 590 nm)? Cf caption Fig 3B, 3C
- line 222 : you could recall the conditions 293 K (fluid solution), 130 K (solution?), 77 K (frozen medium)
- line 230 : the sentence "a kinetic model with two excited states [...] is insufficient" (the dynamics are fitted with four time constants) should be rephrased. If some of these timescales refer to vibrational relaxation within the same electronic state, maybe 2 excited states are sufficient. I wouldn't directly relate the number of time constants with the number of excited states involved.
- line 251 : "losses occur" (if I understand correctly, you could refer to ISC' and ISC" on Fig. 1)
- line 261 : "in the solid state" : meaning amorphous powder or microcrystalline solid, as opposed to frozen solution?
- line 268 : "is lower than that determined in solution" (recall the value of 141 K?)
- line 311 : *LIESST* instead of LIEEST
- line 314 : "In solution [...] the T_{LIESST} value amounts to 141 K and the lifetime [...] to over one million years at 77 K". I find it a little confusing to read in the same sentence a room-temperature property in solution and a low temperature property in frozen medium. Maybe you could rephrase.
- line 475 the Methods section appears in the middle of the bibliography
- line 620 : *after* excitation (not upon)
- line 636 : "as described for the RT measurements" : does this refer to line 600 or line 629?
- line 637 : for all *TA* spectroscopy experiments
- line 638, DFT calculations :
    - I suppose the lowest ^{2}MLCT state is the 3rd excited state *at the LS1 geometry* (hence iroot 3). How did you access the ^{6}MLCT state by TD-DFT? Did you run a sextet single point calculation at the LS1 geometry and TD-DFT optimized *from there*, or did you start *from the HS1 geometry*? This should be specified
    - "Geometry optimizations were performed Atom-pairwise dispersion correction was performed"?
    - why was the def2-SVP basis set used in the specific case of ^{4}MLCT optimization? Or was it used for all TD-DFT MLCT optimizations? This should be clarified
    - why were the calculated transitions shifted by 0.4 eV for LS1 and 0.1 eV for HS1? Was it to match the experimental band shapes?
    - I suppose the TDDFT calculations were TDA-TDDFT. If so, it should be specified
- refs 78, 79, 80 are not used in this work (basis sets for heavy elements)
- ref 86 should probably be https://doi.org/10.1063/1.5143076
- Captions of Table 1 and Fig 21: "obtained from DFT" *and TDA-TDDFT* calculations
- last column of Tables S4, S5 : the "dominant character" of the transitions could be enhanced by setting a threshold, e.g. 10% (as a side remark, I find it surprising that the MC character of states 1 and 2, representing the triply degenerate ground state, is only about 50%, and that the character of state 4, purely red on the metal and purely blue on the ligands, is not more than 60% MLCT).

Review on manuscript NCHEM-26010166A by Kronenberger et al.
"**Covalency control of photomagnetic relaxation in a manganese(II) photoswitch**"

I would like to thank the authors for taking into consideration all my questions and suggestions.
Only two specific items require further attention:

<u>- The covalency of the Mn-C bonds :</u>

- line 128, "as seen in the molecular orbitals"

Visual inspection of the LUMO+5 and LUMO+12 of **LS1** (Suppl. Table 3) (or HOMO/HOMO-1 of **HS1**, Supp. Table 4) does not allow to conclude on this matter. Mn-N bonds are much more ionic than Mn-C bonds, yet their contribution to the dσ* orbitals looks exactly the same. On the other hand, for **LS1**, HOMO-2 as well as HOMO-8 (currently not shown) highlight significant mixing between metallic and carbon-based orbitals (σ and π-bonding). You could be more specific in the text, and add HOMO-8 in Supplementary Table 3 (HOMO-13/HOMO-14 in Supp. Table 4 for **HS1**).

- line 101, "destabilization of the e_g* orbitals relative to the t_{2g} orbitals"

Out of curiosity, I optimized one MnN_6^{2+} complex in its LS and HS states (cartesian coordinates at the end of this document). Given that the computational protocol used by the authors correctly predicts a low spin ground state for complex **1** despite the known HS overstabilization by B3LYP, I assumed state ordering would also be correctly predicted for the model complex. In this case, as expected from the weaker ligand field, a high spin ground state is obtained. The orbitals of **LS1** and LS-tz can then be compared.

	**LS1 (ground state)**	LS-tz
Gibbs free energy	-2717.08549364 Eh	-2624.18170363
HOMO	-5.53 eV	-6.17
e_g1	-0.03 eV (LUMO+5; MnN_2)	-0.52 (LUMO+8; MnN_4)
e_g2	+0.80 eV (LUMO+12; MnC_4)	-0.12 (LUMO+9; MnN_2)
HOMO-e_g1	5.50 eV	5.65
HOMO-e_g2	6.33 eV	6.05
e_g1-e_g2	0.83 eV	0.40

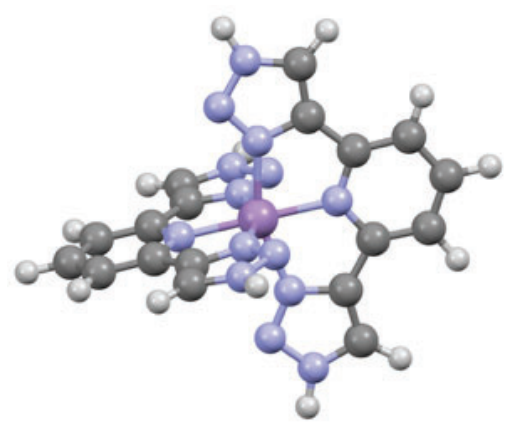
LS-tz

It appears that the HOMO-e_g1 gap is actually slightly smaller in **LS1** than in LS-tz, but that the HOMO-e_g2 gap (involving the carbon atoms) is significantly larger. Both e_g1 and e_g2 being singly populated in the HS state, the destabilization of **HS1** seems to be due to the larger HOMO-e_g2 gap in **LS1**. Maybe you could be more specific in the text (and possibly use the MnN_6^{2+} model compound).

- line 129 "higher displacements Q_{HL}"

Bond elongations between LS and HS forms were compared for **HS1** and HS-tz. Elongations in complex **1** indeed surpass the ones in the MnN_6^{2+} complex. As already noted by the authors by comparing with literature data on iron complexes (because there are no comparable Mn^{II} complexes in the literature), the +0.4 Å Mn-N elongation is outstanding.

	**HS1**	HS-tz (ground state)
Gibbs free energy	-2717.08140976 Eh	-2624.22303696 Eh
Mn-N	+0.400	+0.256; +0.265; +0.228
Mn-C	+0.278	-

- line 110, "larger force constants"

Regarding the steepness of the potential wells, the comparison between rigid terdentate ligands and monodentate ligands, as used by the authors in their response, is convincing enough. There is no need to invoke force constants, which I am also unable to guess.

We thank the reviewer for this thoughtful suggestion to consider the steepness of the HS and LS potential wells and the resulting effect on the activation barrier. We now address the rigidity of the five-membered ring chelating C^N^C ligand, that leads to narrower and stiffer potential wells (as compared to monodentate ligands, for example). However, it is difficult to quantitatively predict the effect on the force constants of the respective LS and HS PESs. We include this suggestion as follows in the main text: "These rigid ligands should also lead to steeper potential wells, i.e. larger force constants in the LS and HS states."

- Compound color :

- I now understand that complex **1** is thermochromic, thank you for clarifying this point. **LS1** appears green at room temperature (inset of Fig. 2D, "green solid at 293 K" on line 145, and "the green color of LS1 arises from..." on lines 160-161) but red at low temperature (inset of Fig. 3A, "Cooling [...] to 95 K [...] resulting in a noticeable color change from green to red" on line 216 and "LS1 appears brownish at low T", line 322). However, on lines 218-219 "Irradiating a frozen solution of **LS-1** at 130 K with a 560 nm LED [...] produces a color change from green to faint orange (Fig. 3A)".
  At this temperature, shouldn't it be going from *red* to faint orange?

- if I understand correctly, **HS1** is pale orange at all temperatures (bleach in Fig.3 and Supp. Fig. 14, discoloration in Fig. 5, orange crystals in Supp. Fig. 28). For clarity, it may be worth saying it explicitly.

- The caption of Supp. Fig. 14 could be further clarified by saying "Irradiation of **LS-1** at λ_{exc} = 560 nm (2 minutes) at 95 K ~~(blue)~~, results in **LS-1** to **HS-1** conversion (blue)."

- Finally, photoswitching is complete after 2 min of irradiation in solution at 95-130 K (Fig. 3) and on paper at 77 K (Fig. 5). However, a longer irradiation time is required to photoswitch the single crystals at 91 K, as shown by the comparison between photos B (2 min) and C (10 min) in Supp. Fig. 28. Maybe this can be mentioned around line 296, as it echoes the discussion on cooperative effects in the microcrystalline solid used in the SQUID measurements (line 283).

Cartesian coordinates optimized in the same conditions as complex **1**

LS-tz

47

Coordinates from ORCA-job LS_triazolyl_opt

Mn	-2.90510343557489	14.27351816813464	4.60285839785563
N	-3.14102090625879	12.31340378961099	4.48924239749540
C	-4.37040985013504	11.78657381126040	4.64219671218548
C	-4.57925078222257	10.41990653770753	4.54458422429175
H	-5.57031518984838	10.00758705877767	4.67105816938971
C	-3.48423316907841	9.60089468083494	4.27769836919144
H	-3.62155623134560	8.53141109990311	4.19403868723263
C	-2.21838035448827	10.15028683456175	4.11927919808412
H	-1.35793051112463	9.52931796717953	3.91344198381782
C	-2.07878986782206	11.52828160658534	4.23486647745856
C	-5.36174606912890	12.82117283437525	4.89591360025798
N	-4.90518294851847	14.11406504614463	4.94133660670750
N	-5.88554041464329	14.93931855322010	5.15474209465050
N	-6.97787383766237	14.18818415088653	5.24717680383329

H -7.87276064372157 14.63019754311190 5.41525263420503
C -6.71911625808886 12.87317198090263 5.09955064432353
H -7.48316689654419 12.11776958220168 5.14635045283319
C -0.86958579230366 12.31715219237774 4.11932419714697
N -1.01706230912066 13.67216970911275 4.28844905873425
N 0.12868454669180 14.27532114269371 4.17367642871739
N 1.01986547516043 13.31522298401307 3.92862344625176
H 1.99225166870129 13.56252756311837 3.79857508027695
C 0.46566567118745 12.08921495685642 3.88472967873630
H 1.03433876656152 11.19560878644705 3.69810402470204
N -2.73815498902459 16.24497223276355 4.64084379893309
C -2.48366327492506 16.87041618561146 5.80385474278089
C -2.36357459722080 18.25416264366224 5.86087387463705
H -2.15762559357255 18.74579071780395 6.80113715157924
C -2.50910374091530 18.97689652418819 4.68397243023727
H -2.41810317180210 20.05448248198543 4.69929175254426
C -2.76718379449851 18.32115349648645 3.48202288133315
H -2.87735819388272 18.86924446701574 2.55705535286526
C -2.87768058659456 16.93975090819115 3.49643849936353
C -2.35810265423893 15.91578222266078 6.88614434884140
N -2.50107202573490 14.59344603479505 6.54186572578083
N -2.35947954672586 13.83141737795910 7.58542991768906
N -2.12688763953808 14.65534033970786 8.60688811430239
H -1.98676472095223 14.27171760434327 9.53251542086984
C -2.11217681416524 15.94981911995946 8.23844472453062
H -1.93816192961640 16.75398853163128 8.93114215543016
C -3.14277637366262 16.05741817272932 2.36932236851017
N -3.19100086247273 14.71313895675840 2.63805495761221
N -3.44482345208937 14.03759117173531 1.55812403946582
N -3.56289646583533 14.93767624346927 0.58754544016416
H -3.76502566524652 14.62846310055679 -0.35465572649574
C -3.38424617595296 16.20080706715831 1.02471892085674
H -3.44278439200443 17.05824581880939 0.37814073978919

HS-tz

47

Coordinates from ORCA-job HS_triazolyl_opt

Mn -2.91336083308435 14.27861000253855 4.51692394130019
N -3.17456603167951 12.04899694529980 4.49132521807730
C -4.38958968301084 11.52030172720388 4.67924482049027
C -4.58965375744836 10.14425965922890 4.67050046902214
H -5.57627216582445 9.73032048734009 4.82408237547605
C -3.48969318177620 9.32249528062679 4.45981572020284
H -3.61420001844987 8.24784836780686 4.44712905352859
C -2.23058645819178 9.87623190330280 4.26588912641807
H -1.36397652168893 9.25155835621217 4.10092694128183
C -2.11162735139246 11.26132466197801 4.28928491239408
C -5.43666520302541 12.51502680398100 4.88465371430309
N -5.10516158273858 13.83959414338301 4.86986072321332
N -6.16061397208854 14.57228897545741 5.06659752680279
N -7.17732427114571 13.72667651839747 5.20924987594573

H	-8.10896001332313	14.08681808287289	5.37370896396271
C	-6.79271418939663	12.44057333713729	5.10721569050644
H	-7.47804401213655	11.61631390859300	5.19636976695138
C	-0.86411013529246	11.99425732001862	4.10523384217621
N	-0.88301368088745	13.35917881994193	4.14064950076774
N	0.31486362966180	13.83291870973904	3.96438160881705
N	1.11147875518986	12.77853271970004	3.81394086950674
H	2.10269547821744	12.91668216890572	3.66122893941785
C	0.44086818027510	11.61376826226074	3.89135896025791
H	0.92009484235706	10.65569033863012	3.79442485190408
N	-2.70354405644324	16.50930385690682	4.60725946022062
C	-2.44825163140436	17.10459011685766	5.77855067787656
C	-2.32531546079052	18.48606861869888	5.87694230546138
H	-2.11940584941431	18.95398452721914	6.82940348801909
C	-2.47462475986355	19.24258911171042	4.72165250911812
H	-2.38424878541147	20.31976417948134	4.76683748003015
C	-2.73937963141905	18.61997608590561	3.50854371612327
H	-2.85778085118012	19.19378174269345	2.60021674070084
C	-2.84792746247418	17.23376177410438	3.49117065884648
C	-2.32039632426279	16.17380778673337	6.89474229202662
N	-2.46988708830767	14.83659359139466	6.66317726568835
N	-2.32252573931990	14.16663528167990	7.76705563133987
N	-2.07764860376083	15.06591475268279	8.71610604886559
H	-1.92914119246910	14.75921095003235	9.66927622577008
C	-2.06341360547818	16.32446904432834	8.23853740583822
H	-1.88092945728710	17.18321180132246	8.86013371190338
C	-3.12264788348324	16.43343684016733	2.30269774313571
N	-3.20396081033691	15.07540841852194	2.42027171329978
N	-3.45445786304093	14.53533549529930	1.26479868814360
N	-3.53564520300762	15.53945204410344	0.39630000723310
H	-3.72805335222471	15.34497172267420	-0.57841186626836
C	-3.33943474816830	16.73738104217204	0.97817073195521
H	-3.36811346357179	17.66408371475343	0.43281095194773
